# filoVision – using deep learning and tip markers to automate filopodia analysis

**DOI:** 10.1242/jcs.261274

**Published:** 2024-02-27

**Authors:** Casey Eddington, Jessica K. Schwartz, Margaret A. Titus

**Affiliations:** ^1^ Department of Genetics, Cell Biology, and Development, University of Minnesota, Minneapolis, MN 55455, USA; ^2^ Graduate Program in Biochemistry, Molecular Biology, and Biophysics, University of Minnesota, Minneapolis, MN 55455, USA

**Keywords:** Filopodia, Deep learning, Computer vision, Image analysis pipeline, filoVision

## Abstract

Filopodia are slender, actin-filled membrane projections used by various cell types for environment exploration. Analyzing filopodia often involves visualizing them using actin, filopodia tip or membrane markers. Due to the diversity of cell types that extend filopodia, from amoeboid to mammalian, it can be challenging for some to find a reliable filopodia analysis workflow suited for their cell type and preferred visualization method. The lack of an automated workflow capable of analyzing amoeboid filopodia with only a filopodia tip label prompted the development of filoVision. filoVision is an adaptable deep learning platform featuring the tools filoTips and filoSkeleton. filoTips labels filopodia tips and the cytosol using a single tip marker, allowing information extraction without actin or membrane markers. In contrast, filoSkeleton combines tip marker signals with actin labeling for a more comprehensive analysis of filopodia shafts in addition to tip protein analysis. The ZeroCostDL4Mic deep learning framework facilitates accessibility and customization for different datasets and cell types, making filoVision a flexible tool for automated analysis of tip-marked filopodia across various cell types and user data.

## Teaser

**Summary:** filoVision is a filopodia analysis platform that uses deep learning and tip markers to measure filopodia, prioritizing automation and flexibility for user data.

## INTRODUCTION

Filopodia are thin, actin-rich membrane projections cells use to explore and interact with their environment (Eilken and Adams, 2010; Mattila and Lappalainen, 2008; Mortimer et al., 2008). They are dynamic structures typically made up of 10–30 parallel, bundled actin filaments that can vary in length, from 1 to 10 µm (Mattila and Lappalainen, 2008; Medalia et al., 2007; Mellor, 2010). Filopodia are initiated from the actin-rich cortex where parallel bundles of actin grow out perpendicular to the plasma membrane. These actin bundles are typically nucleated by the actin polymerases VASP or formin and are cross-linked by actin-binding proteins such as fascin (Jacquemet et al., 2015; Mattila and Lappalainen, 2008). Their formation often requires the action of a MyTH4-FERM (myosin tail homology 4-band 4.1, ezrin, radixin, moesin) myosin, such as the mammalian filopodial myosin 10 (Myo10) or the amoeboid filopodial myosin [*Dictyostelium discoideum* (Dd)Myo7], that collaborates with actin polymerases to initiate and extend filopodia. Filopodial myosins, along with VASP and formin, are robustly localized to the filopodia tip during elongation (Bohil et al., 2006; Petersen et al., 2016; Tuxworth et al., 2001). Increased expression of filopodial proteins such as Myo10 and the actin cross-linker fascin is often associated with greater metastatic potential (Arjonen et al., 2014; Cao et al., 2014; Vignjevic and Montagnac, 2008) and, indeed, metastatic cells make increased numbers of filopodia that are used to move in three dimensions, adhering to and aligning extracellular matrix fibrils (Arjonen et al., 2014; Cao et al., 2014; Jacquemet et al., 2015, 2016; Shibue et al., 2012; Summerbell et al., 2020).

Studies addressing filopodial functions or the mechanism of initiation and extension of filopodia rely on visualizing and measuring filopodia. There are at least three methods for visualizing filopodia: labeling the cell membrane, labeling the actin cytoskeleton and marking the tips of filopodia. The two most common methods of visualizing filopodia are labeling the actin cytoskeleton using the actin-specific probe phalloidin or a marker enriched in filopodia tips that is also present in the cytosol (see, for example, Jacquemet et al., 2019b; Petersen et al., 2016). Filopodial myosins, such as Myo10 and DdMyo7, and the actin polymerases VASP and formin are commonly used to mark the ends of filopodia tips (Dobramysl et al., 2021; Jacquemet et al., 2019b; Kerber and Cheney, 2011; Petersen et al., 2016; Young et al., 2018). This is similar to using a microtubule tip protein such as EB1 to label and track microtubule growth (Vaughan, 2005). Tip markers and actin labels both present certain advantages and disadvantages. Tip proteins clearly mark the tips of filopodia, providing precise identification of their distal end. They have a strong signal-to-noise ratio, allowing for easy detection, and can provide clear signal separation between the cell body and filopodia tips, enabling a well-defined cell edge. However, if filopodia shaft lengths are needed and the cell type is known to have long, curled filopodia, using tip markers alone might not be suitable. If the shaft lengths of long, curled filopodia are needed, an actin label might be more appropriate. However, it can be difficult to define the cell edge without clear separation of the actin-labeled cell body and filopodia stalks, which can cause challenges for identifying the base of filopodia. Thus, although curved filopodia shaft lengths could be more reliably measured with an actin label, the tips and base of filopodia can be better defined using a tip marker.

Many tools have been developed to measure filopodia, demonstrating that there is high demand for workflows that quantify filopodia production. These tools are typically targeted towards a certain cell type and/or filopodia visualization method, and each have their own strengths and limitations (Table S1) (Barry et al., 2015; Driscoll et al., 2019; Jacquemet et al., 2017; Mousavi et al., 2020; Nilufar et al., 2013; Tsygankov et al., 2014; Urbančič et al., 2017). For example, FiloQuant and Filopodyan each have different visualization targets (Jacquemet et al., 2017; Urbančič et al., 2017) – FiloQuant uses an actin label to identify and measure filopodia stalks protruding from a cell body, whereas Filopodyan uses a membrane marker with the option of including a tip marker to measure filopodia and their dynamics over time. To the best of our knowledge, a tool specifically designed to measure filopodia using a tip marker alone or in combination with an actin label does not exist, yet many researchers use tip markers in their filopodia analysis workflow (Petersen et al., 2016; Jacquemet et al., 2019a,b). Furthermore, it can be difficult to find tools adaptable for diverse cell types such as amoeba, likely due to most tools being designed for working with mammalian cells. Thus, filoVision was developed to address the lack of an automated workflow for measuring tip-marked filopodia in diverse cell types such as amoeba.

## RESULTS

### Overview of filoVision – a flexible automated filopodia analysis platform

The filoVision platform contains two notebooks, filoTips and filoSkeleton. filoTips uses deep learning and tip marker signals alone to measure the cell body and filopodia, whereas filoSkeleton uses tip marker signals in combination with an actin label to extract more precise filopodia shaft length information from long, curled filopodia, such as those formed by HeLa cells. The U-net convolutional neural network architecture was chosen because it enables successful model training on very few images compared to other architectures, partially due to its training strategy involving data augmentation (Ronneberger et al., 2015). This has played a large role in the U-net architecture being widely adopted by the biological and medical communities for image segmentation applications. Briefly, the default filoVision U-net models were trained with the ZeroCostDL4Mic platform (von Chamier et al., 2021). This platform enables training models with a graphical user interface, allowing easy training and, more importantly, easy transfer learning capabilities for users to finetune our models to their unique datasets code-free in a timeframe of 1–3 days, which then provides tailored automation for future analyses. After the models classify pixels, the OpenCV library (Bradski, 2000) is used to identify cell bodies and filopodia as objects, allowing their quantification (see Materials and Methods).

### filoTips – filopodia analysis using deep learning and tip markers

**Fig. 1.**
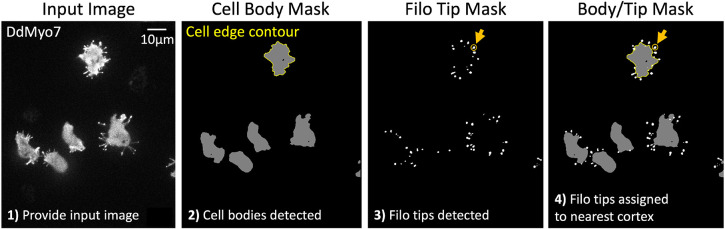
**Filopodia detection and cell assignment by filoTips.** Briefly: (1) An input image of *Dictystelium discoideum* cells expressing eGFP–DdMyo7 is given to filoTips and (2) a mask representing pixels belonging to the background, cell bodies and filopodia tips is generated and cell body objects are identified. (3) Filopodia tip objects are identified. (4) Filopodia are assigned to cell bodies based on distance from the cortex. The objects are indicated by black (background), gray (cell body), yellow (cortex outline) and white (filopodia tips) pixels. Orange arrows and circles indicate a representative filopodia tip.

**Fig. 2.**
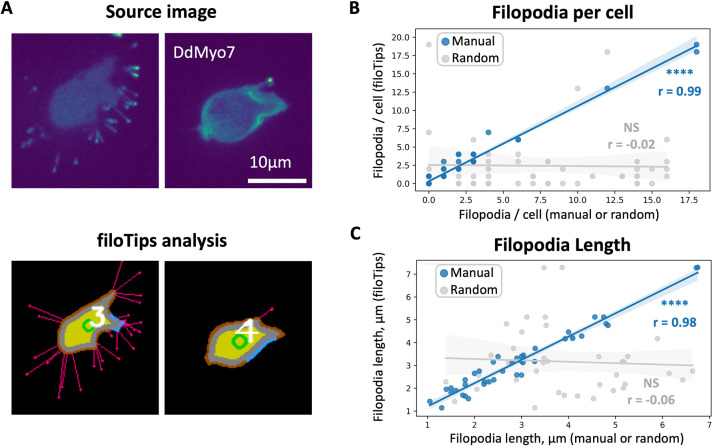
**Automated measurements by filoTips compared to manual measurements.** (A) Representative *D. discoideum* cells making either a high or low number of filopodia and the filoTips analysis annotation for each. Annotation colors: yellow, body; orange, cortex; pink, filopodia; blue, area of highest signal intensity in the cortex; gray, separation between the cortex and body; green, centroid; and white, cell number in image. (B) Correlation of filopodia per cell measurements between filoTips and either manual measurements [Number of imaging experiments (*N*)=6, Number of cells (*n*)=54, r=0.99, ****P=1.37×10^−41^, blue] or a random number control array [*n*=54, r=−0.02, *P*=0.88 (not significant, NS), gray] using two-sided Pearson correlation coefficient (PCC). (C) Correlation of filopodia length measurements (PCC) between filoTips and either manual measurements for 40 random filopodia from 20 random cells (*n*=40, r=0.98, *P*=1.87×10^−26^, blue) or a random number control array (*n*=40, r=−0.06, *P*=0.73, gray).

filoTips was developed to analyze filopodia identified by a tip marker such as Myo10, DdMyo7, VASP or formin. It uses a U-net deep learning model to classify pixels as background, body or filopodia tip. The default model was trained on a dataset of 385 images of live vegetative *D. discoideum* cells expressing DdMyo7 (see Materials and Methods; Table S2). After pixels are classified, OpenCV is used to detect multiple body and tip objects within an image and assigns the filopodia tips to cells based on Euclidean distance (Fig. 1). This enables automated measurements of cell area, perimeter, aspect ratio and filopodia number per cell. It also measures filopodia length, calculated as the linear distance between the cell cortex and filopodia tip, and fluorescence intensity of the tip marker protein within the tip, body and cortex. To establish filoTips as a reliable analysis tool, its output was compared to manual analyses in ImageJ (Schneider et al., 2012) using 54 out-of-sample (independent test dataset previously unseen by model) *D. discoideum* cells expressing mNeonGreen–DdMyo7 (Fig. 2A; Materials and Methods; Table S2). A random number array was used for each correlation calculation as a non-correlated control. There was a strong correlation in filopodia number per cell measurements between filoTips and manual counting, with a Pearson's correlation coefficient of 0.99 (Fig. 2B). Measurements of the lengths of 40 randomly selected filopodia were also strongly correlated between filoTips and manual counting, with a correlation coefficient of 0.98 (Fig. 2C). The length measurement works well when analyzing filopodia that protrude directly out from the cortex, which is common for many cell types. However, cell types such as HeLa can sometimes have long, curled filopodia that, if analyzed with filoTips, could result in incorrect lengths being recorded. For this scenario, filoSkeleton (see below) might be more appropriate because it uses an actin stain to visualize filopodia shafts for length extraction.

Filopodia tip proteins are also present in a visible cytosolic pool (Petersen et al., 2016; Jacquemet et al., 2019b) and this enables filoTips to obtain additional information about their distribution and determine several cell parameters such as cell area, perimeter and aspect ratio. filoTips uses the cytosolic signal to outline the cell body to calculate area and perimeter. It draws a minimum area bounding box for each cell to define its cell body aspect ratio, calculated by dividing the shorter axis by the longer axis, where a score of 1 equals a perfect square. The distribution of tip marker protein in the cell body, cortex and filopodia tips can be determined by calculating the mean fluorescence intensity of each and calculating their ratios. For example, a cortex/body intensity ratio of 1.2 indicates a 1.2-fold enrichment of the tip protein at the cortex compared to that at body. Similar to what was found for filopodia numbers and length, a strong correlation was seen between values obtained from filoTips and manual measurements of cell area and body aspect ratio (Fig. S1A).

### Fine tuning filoTips to diverse cell types

A primary objective of filoTips is to prioritize flexibility so that users can finetune the tool to their own unique datasets and cell types. filoTips models are publicly available (see GitHub repository, https://github.com/eddin022/filoVision); in fact, filoTips will ask the user if they want to use the default model and, if so, users will automatically have instant access without additional steps. However, users can also tune the default model trained on *D. discoideum* cells (see GitHub repository for model links) to their own data by taking advantage of transfer learning capabilities in the ZeroCostDL4Mic 2D multi-label U-net notebook. For example, a laboratory member with no prior deep learning knowledge was able to generate 141 ground truth annotations and finetune the default filoTips model within 48 h with minimal guidance. This initial time investment is worthwhile if the user plans on routinely analyzing hundreds or thousands of filopodia in the future and wants to take advantage of automation.

**Fig. 3.**
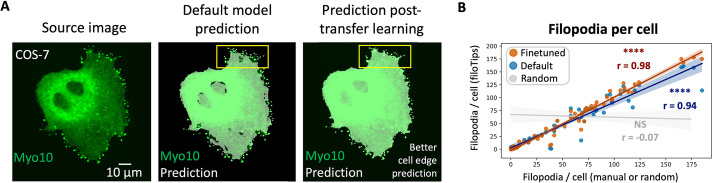
**Tuning filoTips for U2-OS and COS-7 data.** (A) Segmentation predictions before and after transfer learning. Left: source image of a representative COS-7 cell ectopically expressing eGFP–Myo10. Middle: prediction made by the default filoTips model. Right: prediction made after performing transfer learning on U2-OS and COS-7 data. The yellow rectangle highlights an example of improved cell edge detection after transfer learning. (B) Filopodia per cell measurement correlation (PCC) between filoTips using the default model and manual measurements (*n*=56, r=0.94, blue) for the U2-OS and COS-7 finetuned model and either manual measurements (*N*=6, *n*=56, r=0.98, ****P=2.03×10^−39^, orange) or a random number control array (*n*=56, r=−0.08, *P*=0.56, gray).

The adaptability of filoTips was demonstrated using transfer learning. This was conducted using 89 images of U2-OS and COS-7 cells (containing 50 U2-OS cells and 50 COS-7 cells) expressing eGFP–Myo10 or mCherry–Myo10 (70% for training and 30% for validation) that differed from those used in default training dataset acquisition (Materials and Methods; Table S2). The data augmentations shift, zoom, shearing, flip and rotation were used to increase the size of the training data (Ronneberger et al., 2015). A model unseen testing dataset consisting of 52 total U2-OS and COS-7 images (containing 26 U2-OS cells and 30 COS-7 cells) was used to evaluate model pixel predictions (Tables S2 and S3). It was found that the default filoTips model trained on amoeboid cells was able to reliably predict U2-OS and COS-7 filopodia tips. Filopodia counts using the default filoTips model (prior to finetuning) were strongly correlated with manual counts in ImageJ (r=0.94; Table S6). However, cell edge prediction accuracy dropped, perhaps due to a less defined cell edge provided by the Myo10 signal compared to that provided by DdMyo7 expressed in amoeboid cells (see Fig. 3A for an example of pre- and post-transfer learning segmentations for Myo10). By starting with the weights from the default filoTips model trained on amoeboid cells expressing DdMyo7 and using the U2-OS and COS-7 datasets for further training and testing, the model that underwent transfer learning was able to better predict U2-OS and COS-7 cell edges marked by Myo10 (Fig. 3A). To ensure filopodia detection accuracy after transfer learning, filopodia number measurements acquired by filoTips using the finetuned model (see GitHub repository for model link) were compared to manual measurements of 56 out-of-sample cells (26 U2-OS and 30 COS-7 split) expressing eGFP–Myo10 or mCherry–Myo10. Measurements of filopodia number per cell strongly correlated between filoTips and manual counts, with a correlation coefficient of 0.98 (Fig. 3B; Table S6), demonstrating accurate filopodia tip detection. The example presented here shows that filoTips can be successfully tuned for different cell types and user data in a relatively short period.

### Relationship between Myo10 and DdMyo7 expression and filopodia formation

**Fig. 4.**
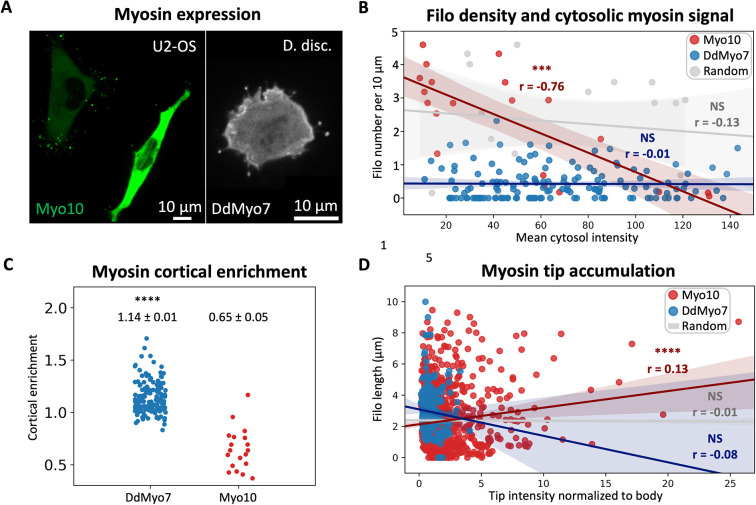
**Correlation of ectopic Myo10 and DdMyo7 expression levels with filopodia formation.** (A) Representative images of U2-OS cells transiently expressing eGFP–Myo10 and a *D. discoideum* cell expressing GFP-DdMyo7. (B) Correlation of filopodia density and cytosolic myosin signal (PCC) for Myo10 (*N*=2, *n*=20, r=−0.76, ***P=0.00011, red), DdMyo7 (*N*=3, *n*=153, r=−0.01, *P*=0.91, blue), and a random number control array (*n*=20, r=−0.13, *P*=0.58, gray). (C) Cortical enrichment measurements for DdMyo7 (*N*=3, *n*=153, mean=1.14, s.e.m.=0.01, blue) and Myo10 (*N*=2, *n*=20, mean=0.65, s.e.m.=0.05, red). Statistical analyses were performed by two-sided two-tailed unpaired Student's *t*-test, ****P<10^−9^. (D) Correlation between myosin tip signal normalized to body and filopodia length (PCC) for Myo10 (*N*=2, *n*=1008, r=0.13, ****P=6.1×10^−05^, red), DdMyo7 (*N*=3, *n*=280, r=−0.08, *P*= 0.21, blue) and a random number control array (*n*=1008, r=−0.01, *P*= 0.71, gray).

Measurements of filopodia and localization or levels of filopodia proteins can provide insight into the mechanism of their formation. The MyTH4-FERM myosins DdMyo7 and Myo10, from amoeba and mammalian cells, respectively, are both essential for filopodia formation (Bohil et al., 2006; Petersen et al., 2016; Tuxworth et al., 2001). However, it is unclear whether they promote filopodia formation via a similar mechanism. Therefore, U2-OS cells expressing eGFP–Myo10 and *D. discoideum* cells expressing eGFP–DdMyo7 were used to directly observe and compare the relationship between myosin expression levels and filopodia numbers (Fig. 4A). filoTips was used to analyze 1008 filopodia over 20 U2-OS cells transiently expressing eGFP–Myo10 or mCherry–Myo10 and 280 filopodia over 153 *D. discoideum* DdMyo7-null cells expressing GFP-DdMyo7 in less than an hour, which otherwise could not be done using existing methods, further highlighting the advantage of using filoVision. The relationship between cell size, or cell perimeter (µm), on filopodia number per cell was investigated first. As expected, a strong correlation was observed for the perimeter of U2-OS cells expressing Myo10 and filopodia number (correlation coefficient of 0.85), with larger cells forming more filopodia (Fig. S2A). A weaker correlation between cell perimeter and filopodia number was observed for *D. discoideum* cells expressing DdMyo7 (correlation coefficient of 0.25), suggesting that cell size has a stronger impact on filopodia number in U2-OS cells than in *D. discoideum*, perhaps because of the inherently smaller size of *D. discoideum*. Because cell size impacts filopodia number, it could be argued that filopodia density or filopodia number normalized to cell perimeter (number of filopodia per micrometer) is a more useful metric than the raw filopodia number per cell. Thus, filoTips provides the filopodia number per 10 µm measurement in addition to raw filopodia number per cell to account for cell size and measure filopodia density.

Filopodia numbers are reduced when either Myo10 or DdMyo7 protein levels are depleted in mammalian or amoeboid cells, respectively (Bohil et al., 2006; Petersen et al., 2016). Conversely, higher Myo10 expression levels are associated with increased numbers of filopodia in mammalian cells (Bohil et al., 2006; Jacquemet et al., 2017); however, it is unclear whether overexpression of DdMyo7 relates to amoeboid filopodia number. Therefore, the relationship between cytosolic DdMyo7 and Myo10 protein levels and filopodia density was determined. No correlation was observed between the mean cytosolic intensity of DdMyo7 and filopodia density (Fig. 4B), revealing that DdMyo7 overexpression does not strongly impact filopodia density in amoeboid cells. Because previous reports showed that overexpression of Myo10 promotes increased filopodia number (Bohil et al., 2006; Jacquemet et al., 2017), it was expected that the mean cytosolic Myo10 signal would have a strong positive correlation with filopodia density; however, surprisingly, the opposite result was observed. A strong negative correlation between cytosolic Myo10 and U2-OS filopodia density was measured, with a correlation coefficient of −0.76 (Fig. 4B). This suggested that as ectopically expressed cytosolic Myo10 decreased, filopodia density actually increased. The low cytosolic Myo10 signal observed for cells making relatively high filopodia could be due to Myo10 localizing from the cytosol to filopodia tips. Therefore, the total fluorescence intensity of Myo10 (the sum of Myo10 signal in the cytosol and all filopodia tips) was calculated for each cell and a correlation coefficient of −0.47 was measured when filopodia tip signal was included, demonstrating a weaker yet also negative correlation, even with the Myo10 tip signal included (Fig. S2B). This suggests that increased levels of cytosolic Myo10 did not correspond to increased filopodia as expected, and it is possible that under certain conditions or in certain cell types, highly expressed Myo10 is maintained in an autoinhibited state. This could, perhaps, be due to lower phosphatidylinositol (3,4,5)-trisphosphate production required for relief of Myo10 autoinhibition (Umeki et al., 2011), maintaining Myo10 in a cytosolic pool.

DdMyo7 is targeted to the cortex, particularly during cell migration, and a 1.2-fold cortical enrichment is typically observed (Arthur et al., 2019, 2021). filoTips was used to compare DdMyo7 cortical enrichment in *D. discoideum* to Myo10 cortical enrichment in U2-OS cells. A cortical enrichment value of 1.14±0.01 (mean±s.e.m.) was observed for DdMyo7, similar to previous reports (Arthur et al., 2019, 2021). However, a low cortical enrichment value of 0.65±0.05 was observed for Myo10 in U2-OS cells, suggesting that the mean Myo10 signal is actually stronger in the cell body compared to that in the cortex or cell edge (Fig. 4C). Myo10 has been shown to be enriched in membrane ruffles (Berg et al., 2000); however, under conditions typical for imaging U2-OS cells making filopodia, Myo10 cortical enrichment was not observed. This might be due to the relatively limited migration of these cells compared to that of *D. discoideum*.

Myo10 is a relatively fast motor thought to have a role in transporting the actin polymerase VASP to filopodia tips (Kerber et al., 2009; Ropars et al., 2016; Tokuo and Ikebe, 2004). Due to the fast velocity of Myo10 (∼600 nm/s; Kerber et al., 2009; Ropars et al., 2016) outpacing the much slower rate of mammalian filopodia extension (variable depending on cell type, but example report of 55 nm/s in the mouse root ganglion growth cone; Brown and Bridgman, 2003), Myo10 can be seen accumulating in the tips of filopodia as they elongate during extension. This reportedly results in an enriched Myo10 signal in the tips of relatively longer filopodia compared to tips belonging to shorter filopodia (Fitz et al., 2023). In contrast, previous reports suggest that the DdMyo7 signal in filopodia tips remains constant throughout extension (Arthur et al., 2019). To more directly compare the tip accumulation of the two myosins, filoTips was used to quantify filopodia length and tip signal intensity of Myo10 and DdMyo7 (normalized to body signal). Similar to previous reports (Fitz et al., 2023; Tokuo and Ikebe, 2004), there was a weak but significant positive correlation (r=0.13) between Myo10 signal in filopodia tips and filopodia length, with longer filopodia more likely to have a stronger Myo10 tip signal (Fig. 4D). However, unlike Myo10, no correlation was detected between the DdMyo7 signal in filopodia tips and filopodia length (Fig. 4D), consistent with DdMyo7 not accumulating in filopodia tips over time during extension, unlike what has been observed for Myo10 (Fitz et al., 2023; Arthur et al., 2019). Interestingly, this suggests that once a *D. discoideum* filopodium extends, a constant level of DdMyo7 is maintained at the tip and elongation does not require continued increases in DdMyo7 levels, unlike what is seen for filopodia formed by Myo10. Collectively, these observations show that although Myo10 and DdMyo7 have some common features (Petersen et al., 2016; Arthur et al., 2019), there are aspects of their role in filopodia elongation that differ.

### filoSkeleton – filopodia analysis using tip markers coupled with actin labeling

Characterization of filopodia is often carried out on cells stained for or expressing both actin and tip proteins. filoSkeleton was developed to use a tip marker in combination with an actin label that allows for detection of cell bodies, filopodia stalks or shafts, and the far ends of filopodia. Briefly, it uses two deep learning models and OpenCV (Bradski, 2000) to segment and detect cell bodies, filopodia stalks and filopodia tips within an image. filoSkeleton checks each filopodia tip to see whether the tip overlaps with or neighbors a stalk (maximum distance of 3 pixels) and, if so, identifies the tip as a filopodium (Fig. S3). Starting at the tip, filoSkeleton finds the direction of the nearest cortex and, in that direction, moves 5 pixels at a time along the stalk until it reaches the cortex/filopodia base interface.

**Fig. 5.**
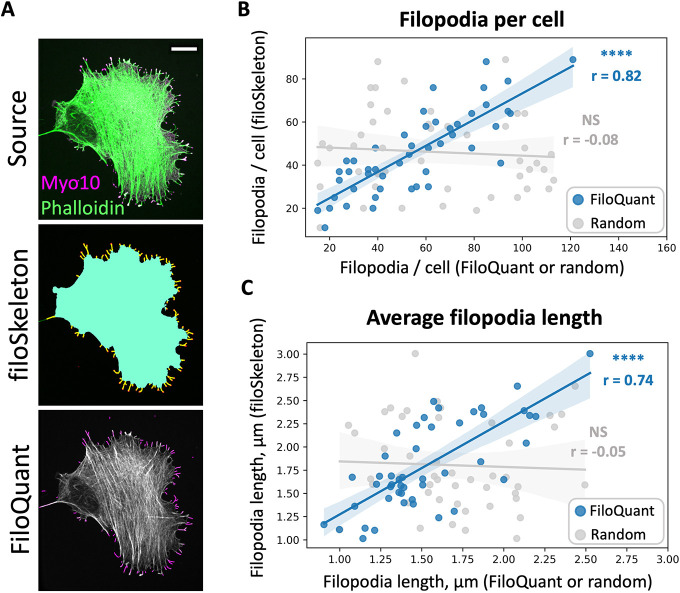
**Comparison of filoSkeleton measurements with FiloQuant analysis.** (A) Representative HeLa cell stained with Alexa Fluor 488–phalloidin (green) to visualize the actin cytoskeleton and anti-Myo10 antibodies (magenta) showing the filoSkeleton analysis annotation. filoSkeleton annotation: aqua, body; yellow, filopodia stalks; red, filopodia tips. FiloQuant annotation: purple, filopodia stalks. Scale bar: 10 µm. (B) Correlation of filopodia per cell measurements (PCC) between filoSkeleton and either FiloQuant (*N*=5, *n*=47, r=0.82, ****P=3.07×10^−12^, blue) or a random control array (*n*=47, r=−0.08, *P*=0.60, gray). (C) Correlation of average filopodia length measurement (PCC) between filoSkeleton and either FiloQuant (*N*=5, *n*=47, r=0.74, ****P=3.0×10^−09^, blue) or a random control array (*n*=47, r=−0.05, *P*=0.76, gray).

filoSkeleton provides cell body measurements that include cell area, aspect ratio and circularity, in addition to perimeter and average filopodia per micrometer records. Tip marker signal intensity in the cell body and filopodia tips are also recorded to observe tip marker protein distribution. The performance of filoSkeleton was assessed by comparing its output to manual measurements of the same dataset. As seen for filoTips, filoSkeleton performed well, with a Pearson's correlation coefficient of 0.997 for cell area and 0.791 for body aspect ratio (Fig. S1B). There was also high correlation for average pixel intensity measurements of tip proteins. Measurement of filopodia number and length were made using filoSkeleton or FiloQuant to compare the performance of the two tools. FiloQuant (Jacquemet et al., 2017, 2019a) was chosen for the filopodia number and length comparison because it uses an actin stain to analyze filopodia, similar to filoSkeleton. A total of 47 out-of-sample cells (24 U2-OS and 23 HeLa) stained for actin with fluorescent phalloidin and immunostained for Myo10 were analyzed (Fig. 5A). For FiloQuant analysis, cells were manually cropped, and parameters (cell edge and filopodia) optimized for all cells individually to obtain the most accurate readings possible. A strong correlation between filoSkeleton and FiloQuant filopodia number measurements was observed, with a correlation coefficient of 0.82 (combined U2-OS and HeLa, r=0.82; U2-OS alone, r=0.81; HeLa alone, r=0.83; Fig. 5B), demonstrating similar filopodia number measurements for both filoSkeleton and FiloQuant. Average filopodia lengths were also compared and a strong correlation between filoSkeleton and FiloQuant was seen, with a correlation coefficient of 0.74 (Fig. 5C).

## DISCUSSION

filoVision is a new deep learning filopodia analysis platform that accurately measures filopodia number, length and tip intensity using filopodia tip markers. It enables automated, highly reproducible and highly adaptable analysis of filopodia using either a tip marker alone or in combination with an actin label to extract information about cell bodies, filopodia and tip protein measurements in two-dimensional images. It does not rely on manual parameter tuning to identify filopodia, removing user bias and increasing reproducibility. If different laboratory members use the same models, they will consistently get the same results.

The ability of filoVision to analyze multiple cells simultaneously within the same image allows the user to save time spent cropping images or manually tuning parameters to each cell, which can cause filopodia analysis to become tedious. filoTips was shown to be able to analyze hundreds of U2-OS or *D. discoideum* filopodia in less than an hour, which would not be possible using existing methods. It should be noted that the default filoTips model trained on amoeboid cells was surprisingly able to reliably detect Myo10-marked U2-OS filopodia tips, with only relatively minor underestimates of cell edges needing finetuning. However, the option for transfer learning allows filoVision to be finetuned to different cell types or imaging conditions (Fig. 3B). Lastly, both notebooks can also be run and edited locally with Python, which does not require proprietary software and is designed for biologists who might not have experience setting up a coding environment. The option of running filoVision in a cloud-based, pre-initialized Google Colab environment is available with no software or hardware requirements (see GitHub repository). Ultimately, this results in flexible, automated and rapid tip-marked filopodia analyses with high reproducibility and low user bias that can be run in a cloud environment or locally.

### Limitations and other considerations

There are some limitations to filoVision that users should keep in mind. As with all image analysis tools, the quality of the image is paramount. There should be a strong signal for the tips, shaft or cell body, which should also be noticeably above that of the background. A faint tip signal, in particular, can lead to an underestimation of filopodia number. As with other filopodia analysis workflows, it is necessary to avoid densely plated samples to prevent overlap between filopodia and neighboring cells. This can result in obscured filopodia and an underrepresentation of filopodia number or assignment of filopodia to the incorrect cell. filoTips uses Euclidean distance to assign filopodia tips to cell bodies. Therefore, cells should be plated at a density at which filopodia tips are closer to the bodies that they belong to as opposed to neighboring cell bodies. The exact density will depend on the cell type and their average filopodia length. This limitation is minor considering most users will plate cells at a low density regardless of analysis method to prevent occlusion of filopodia by neighboring cells and their filopodia. Occasionally, while tracking a filopodia shaft, filoSkeleton can confuse the correct shaft with one that partly overlapped and begin tracking that shaft back to the cortex. In our experience, the error associated with this was usually minor as the relatively rare crossing events were more likely to occur near the cortex and had similar remaining shaft lengths from the crossing event to the cortex. However, in some cases, it will cause incorrect length measurements. The annotations make it so that users can see if an event like this occurred, and, if so, all other measurements including filopodia number per cell remain valid, but the shaft length of that event will be inaccurate.

The current filoVision models are tuned for our *D. discoideum*, U2-OS, COS-7 or HeLa cell images. The default model trained on *D. discoideum* cells was found to perform surprisingly well at predicting filopodia tips for U2-OS and COS-7 cells, considering that it had not been exposed to these cell types, but required some adjustment to better predict the relatively dim edge of these cells. After performing transfer learning with the default filoTips model weights and 89 images of 100 U2-OS and COS-7 cells, the finetuned model did a substantially better job predicting the cell edge. Although using the filoVision models provided is likely to result in reasonable annotations of cells and filopodia tips, especially for users working with cell types matching our model training data, model performance would be at its highest by tuning the existing filoVision models by performing transfer learning with a user's images of the cell type and typical imaging conditions. This can easily be accomplished with the 2D U-net ZeroCostDL4Mic notebooks. The initial time investment in transfer learning is likely to be an acceptable trade-off for future, efficient automation if the user plans on analyzing hundreds or thousands of filopodia, especially if the user commonly uses a lone tip marker to analyze filopodia and wants to take advantage of an automated workflow.

Many filopodia analysis tools have been developed with a specific purpose in mind; therefore, it is crucial to consider the analysis goals of both the user and the tool. filoVision is an excellent choice if the potential user routinely analyzes hundreds or even thousands of tip-marked filopodia. Its advantage becomes even more apparent when additional cell or tip protein signal information is required, or if speed, reproducibility and automation are top priorities. However, there are also scenarios in which filoVision might not be the ideal choice. For those with smaller datasets or infrequent filopodia analysis needs, manual methods or a tool such as FiloQuant might be more efficient (Jacquemet et al., 2017). Also, if the user lacks tip-labeled data, FiloQuant or Filopodyan (Urbančič et al., 2017) is a better fit depending on the cell marker. If three-dimensional image analysis is required, U-shape 3D (Driscoll et al., 2019) should be considered instead.

### Conclusion

Filopodia tools have been primarily developed for the analysis of actin- or membrane-labeled mammalian cells that are relatively large (30–100 µm in diameter) and, as such, many are not optimized for smaller, less common cell types such as amoeba (10 µm in diameter). Although labeling filopodia tips is common (for examples, see Jacquemet et al., 2019b; Kerber and Cheney, 2011; Petersen et al., 2016), to our knowledge, an automated tool that uses tip labeling alone to quantify filopodia does not currently exist. These were the core limitations that motivated the development of filoVision. To address the lack of flexibility for user data or cell type, filoVision takes advantage of the transfer learning capabilities of the accessible ZeroCostDL4Mic platform. It also enables rapid live cell analysis with a lone tip marker when the cytosolic signal is sufficiently high, and this cytosolic fraction can be used to detect and measure the cell body (filoTips). Alternatively, the tip marker can be combined with actin staining to obtain similar measurements, with the benefit of extracting additional filopodia shaft information (filoSkeleton). Currently, filoVision is being expanded to include tracking filopodia tips over time in live-cell experiments and will include the ability to use more than one tip marker for extracting co-localization information about different tip marker proteins, providing the ability to gain even more insight into the role of different filopodia tip proteins and their collaboration during filopodia formation.

## MATERIALS AND METHODS

### filoTips

#### Default model training

##### Overview

filoTips takes advantage of the U-net neural network architecture to generate image segmentations predicting pixel classes as either background, cell body or filopodia tip, represented by values 0, 1 and 2, respectively. ZeroCostDL4Mic was selected for the training workflow because the framework enables easy, graphics user interface-based model training and transfer learning (von Chamier et al., 2021). Training was performed using the U-net 2D multi-label notebook. After generating pixel predictions, filoTips then leverages OpenCV to identify and measure cell bodies and their corresponding filopodia by converting the segmentation information into measurable objects.

##### Training data description

The training dataset consisted of 385 images of live vegetative *Dictyostelium discoideum* cells. Wild-type and filopodia-mutant amoeba (*myo7* or *vasp* null; Tuxworth et al., 2001; Han et al., 2002) expressing DdMyo7 tagged with GFP, mCherry or mNeonGreen were included in the training dataset (see Table S2 for complete breakdown) (Arthur et al., 2019, 2021; Petersen et al., 2016).

##### Ground truth generation

The source images were paired with target annotations, or ground truths, that describe the images, demonstrating the correct class (background, cell body or filopodia tip) each pixel should be assigned to (Fig. S4A). Generating ground truths for training can be a tedious task; thus, tools such as Amazon SageMaker (https://aws.amazon.com/pm/sagemaker/), V7 labs (https://www.v7labs.com/), Labelbox (https://labelbox.com/) and ilastik (Berg et al., 2019) can be used to expedite the image labeling process for deep learning applications. ilastik is free and is designed for the biomedical community (Berg et al., 2019); thus, its pixel classification mode was chosen to generate ground truths for the default filoTips model (Fig. S5A). ilastik was provided with batch sizes of 20 images, all features were used and set to the highest settings, and three labels were selected (0, background; 1, cell body; and 2, filopodia tips). Using ‘paintbrush’ for each label, ilastik was shown the correct assignment by the user and began to auto-assign pixels. This process was repeated until all pixels in the 20-image batch had been assigned the correct label. The ground truths were exported as simple segmentation .tiff files and a new project was started for the next batch of images until ground truths were generated for all data.

##### Training parameters

The source images and ground truths, along with the total number of labels (three), were provided for default filoTips model training. The data augmentations shift, zoom, shearing, flip and rotation were used to artificially increase the size of the training data (Ronneberger et al., 2015). The following training parameters were used: epoch number, 300; image patches, 279; patch size, (512,512); batch_size, 4; number of steps, 0; pooling steps, 2; percentage validation, 20; initial learning rate, 0.0003; patch dimensions, 512×512; minimum fraction, 2%; and loss function, acategorical_crossentrop (see Fig. S4B for training and validation loss).

##### Model evaluation

To test the ability of the model to generate accurate predictions, segmentation predictions for 76 out-of-sample test images were compared to ground truths and scored using metrics such as intersection-over-union (IoU), F1 and panoptic scores (Table S3, Fig. S4A). IoU measures the overlap between predicted and ground truth segmentation masks using the ratio of their intersection to their union and is provided by ZeroCostDL4Mic after model evaluation. The F1 score combines precision and recall into a single value to assess the accuracy of the segmentation. filoVision uses semantic, not instance, segmentation (U-net, Ronneberger et al., 2015), but panoptic quality, which is typically used to evaluate predictions that involve the combination of semantic and instance segmentation for categorizing objects, was also calculated as part of the comprehensive evaluation process.

#### Filopodia detection and extraction of measurements

##### Detection and measurement of cell bodies

filoTips uses OpenCV (Bradski, 2000) contours to convert model segmentation predictions into individual cell body and filopodia tip objects (Fig. 1). Pixels belonging to the body class in the segmentations are extracted. Contour detection then searches for connecting body pixels and provides the contour or outline of the connected pixels. This allows cell body object assignment with a numerical identifier and provides body object coordinate information. If multiple cell contours are detected, the cell body contours are measured iteratively using OpenCV image moments such as contourArea and arcLength to extract pixel measurements, which are converted to micrometer measurements. Image moments within the OpenCV library such as those above enable easy calculations of metrics such as area, perimeter, aspect ratio, centroid and circularity using the contours as a reference point. The cortex is identified by increasing the thickness of the contour edge (cell edge) outline by 15 pixels and assigning the thin ∼6-pixel-wide inner band that overlaps with the existing cell contour as the cortex, or cell body edge (marked as blue and orange in filoTips annotations). Another ∼15-pixel-wide gray band is introduced to separate the cortex (blue or orange) and cell body (yellow) for more accurate tip protein signal assignment (cortex or body) during extraction (Fig. 2A, bottom). Tip protein signal in the body and cortex is then extracted from the image via pixel assignment (body or cortex object) and recorded coordinates. To enable measurement of the asymmetrical enrichment of DdMyo7 at the cortex, a metric was included that scans for the strongest signal within the cortex, extracts signal from the surrounding ∼50 cortex pixels and labels it the ‘leading edge’ (the blue section of the cortex in filoTips annotations). Tip marker signal ratios (cortex/body, tip/body, etc.) for each section are calculated and included in the final summary table.

##### Detection and measurement of filopodia tips

After all cell bodies have been detected, contour detection is again performed to detect filopodia tips. During cell body analysis, cell outline coordinates are saved and referred to when assigning filopodia tips to cells. Iteratively for each detected tip contour, the tip protein signal is extracted from the contour via recorded pixel coordinates and a tip/body signal ratio is calculated. The Euclidean distances from the tip to all outline coordinates are calculated and the tip is assigned to the closest cell contour. If a cell outline is not within 10 µm of the tip, it is considered an artifact and not recorded, otherwise this linear distance is recorded as the filopodium length (pink, Fig. 2A, bottom). Again, for many cell types including amoeba, this is quite effective for getting accurate lengths. However, if a potential user requires shaft length of long, curled filopodia, filoSkeleton or FiloQuant would be more appropriate. All filopodia analysis methods require separation of cell bodies to avoid body and filopodia overlaps and thus loss of filopodia signal. Because of this spatial separation, it is effective to use Euclidean distance to assign filopodia tips to the nearest cell cortex. Sometimes this results in a filopodia tip being assigned to the wrong cell if neighboring cells are closer to the tip than the cell it belongs to; however, evaluations of filoTips suggest that this is not common. Owing to the nature of filopodia analysis, cells should be plated at low densities to avoid occlusion of filopodia; therefore, this is a minor limitation. A record is kept of the number of filopodia tips assigned to the different cell body objects and, after all filopodia have been detected, the filopodia number per cell metric is calculated for each cell. Lastly, the annotations (Fig. 2A) are exported along with summary tables (see Table S4 for examples) for cell body and filopodia information extraction.

#### Transfer learning

ZeroCostDL4Mic 2D U-net multi-label notebook (von Chamier et al., 2021) was used to perform code-free, low-barrier transfer learning to finetune filoTips for U2-OS and COS-7 cells. A total of 89 images consisting of 100 mammalian cells (50 COS-7 and 50 U2-OS) expressing either mCherry–Myo10 or eGFP–Myo10 were used (see Table S2 for breakdown). Similar to training the default filoTips model, these source images were paired with ground truths and used for transfer learning via the 2D U-net multi-label notebook. Ground truths were generated via the ImageJ macro ‘filoTips Ground Truth Generator’ (GitHub filoVision repository), which allows the user to threshold cell bodies and filopodia tips, then, using a series of erosions and dilutions, removes filopodia tips, leaving a mask of the cell body (Fig. S5B). The resulting cell body mask for each cell was closely inspected and, if necessary, minor corrections were made manually as part of the macro. We tried using ImageJ thresholding methods to acquire masks of filopodia tips and had some success, but we found that the default filoTips model was able to better predict filopodia tips than the thresholding methods used in ImageJ. Thus, the default filoTips prediction of filopodia tips was incorporated into the ImageJ macro to obtain a starting point for filopodia tip ground truths. As with the method for obtaining the cell body mask, minor corrections can be made to filopodia tip annotations if needed.

##### Training parameters

The source images and their annotation pair were provided for the training of the filoTips model finetuned to mammalian cells, undergoing a 70% training/30% validation split. The following training parameters were used: previous model weights were the default filoTips model; labels, 3; epoch number, 800; image patches, 318; patch size, (512,512); batch size, 4; number of steps, 0; pooling steps, 2; percentage validation, 30; initial learning rate, 4.7×10^−6^; patch width and height, 512; minimum fraction, 0.02; and loss function, acategorical_crossentrop. The data augmentations shift, zoom, shearing, flip and rotation were used to artificially increase the size of the training data. 56 out-of-sample cells (30 COS-7 and 26 U2-OS) expressing eGFP–Myo10 or mCherry–Myo10 were analyzed with filoTips using the finetuned model and compared to manual measurements (Table S6).

### filoSkeleton

#### Model training

##### Overview

Unlike filoTips, filoSkeleton uses two U-net neural networks in parallel, one to predict filopodia tips using a tip marker (0, background; 1, filopodia tips) and the other to predict cell body and filopodia stalks using an actin stain (0, background; 1, cell body; 2, filopodia stalk). Each of these models were trained separately using ZeroCostDL4Mic as the framework for the training workflow; models were trained using the U-net 2D notebook and U-net 2D multi-label notebook, respectively (von Chamier et al., 2021). filoSkeleton also leverages OpenCV to identify objects; however, it uses segmentations from both models to identify and measure cell bodies, filopodia stalks and filopodia tips.

##### Ground truth generation and training parameters

###### Cell body and filopodia stalk model

The model for segmenting cell bodies and filopodia stalks was trained on a dataset of 86 phalloidin-stained U2-OS cells (see Table S2). Ground truths for cell bodies and filopodia stalks were generated using the ‘filoSkeleton Body_Stalk Ground Truth Generator’ ImageJ macro (GitHub filoVision repository; Fig. S5C), which masks the cell, shaves off the filopodia stalks through five rounds of erosion and dilution to isolate the cell body pixels, similar to workflows such as ADAPT (Barry et al., 2015), then generates a ground truth segmentation consisting of three labels (0, background; 1, cell body; 2, filopodia stalks) (Fig. S4C). The source images and ground truths, along with the total number of labels (three), were provided for training. The data augmentations shift, zoom, shearing, flip and rotation were used to artificially increase the size of the training data. The following training parameters were used: epoch number, 500; image patches, 174; patch size, (512,256); batch_size, 4; number of steps, 0; pooling steps, 2; percentage validation, 20; initial learning rate, 0.0003; patch dimensions, 512×512; minimum fraction, 2%; and loss function, acategorical_crossentrop (see Fig. S4D for training and validation loss).

###### Filopodia tip model

The model for segmenting filopodia tips was trained on a dataset of 121 images of U2-OS cells ectopically expressing eGFP–Myo10 and immunostained for FMNL3 (see Table S2 for breakdown). Ground truths for filopodia tips were generated with ilastik (see filoTips: ‘Ground truth generation’; two classes: 0, background, and 1, filopodia tips; Fig. S4E). The data augmentations shift, zoom, shearing, flip and rotation were used to artificially increase the size of the training data. The following training parameters were used: epoch number, 600; image patches, 133; patch size, (512,256); batch_size, 4; number of steps, 0; pooling steps, 2; percentage validation, 20; initial learning rate, 0.0003; patch dimensions, 512×512; minimum fraction, 2%; and loss function, acategorical_crossentrop (see Fig. S4F for training and validation loss).

##### Model evaluation (both models)

Cell body and stalk ground truths and filopodia tip ground truths were generated for 45 out-of-sample test images (see Table S2 for breakdown) using the same methods for obtaining training data ground truths. As for filoTips, the ground truths were compared to predictions made by the filoSkeleton models and scored using IoU, F1 and panoptic evaluation metrics (Table S3, Fig. S4C,E).

#### Filopodia detection and extraction of measurements

filoSkeleton uses OpenCV contours (Bradski, 2000) to convert model segmentation predictions into cell body, filopodia stalk and filopodia tip objects (Fig. S3). Similar to filoTips, filoSkeleton uses contour detection to first detect the edges of all cell bodies in an image and assign them as cell body objects (aqua, Fig. 5A). For each cell body object, related measurements are extracted iteratively as for filoTips (see the ‘filoTips – Default model training’ and ‘filoTips – Filopodia detection and extraction of measurements’ sections). After all cell bodies have been detected, contour detection is performed on the stalk segmentation to detect filopodia stalks and record their coordinates. Finally, contour detection is used to locate filopodia tip foci. If a tip focus is within 3 pixels of a filopodia stalk, it is assigned as a filopodium. For each detected tip contour, the tip protein signal is extracted and a tip/body signal ratio is calculated. Starting at the tip, filoSkeleton finds the direction of the nearest cortex and, in that direction, moves 5 pixels at a time along the stalk until it reaches the cortex or filopodia base interface (yellow stalk with a red tip). This provides filopodia shaft length information and allows filoSkeleton to detect broken or disembodied filopodia and ignore them if they are at least 5 pixels from the cortex (yellow stalk with a blue tip). A record is kept of the number of filopodia tips assigned to the different cell body objects and, after all filopodia have been assigned, the filopodia number per cell metric is calculated. Lastly, annotations (Fig. 5A) are exported along with summary tables (see Table S5 for examples).

### Manual quantification of filopodia – analysis comparison and statistics

filoTips measurements were compared to manual measurements in ImageJ using base tools such as line, oval and polygon (Schneider et al., 2012). Cell bodies were outlined manually in ImageJ using the polygon tool and metrics such as cell area and aspect ratio were calculated using the ‘measurements’ tool. Fluorescent protein intensities were measured by outlining the body, cortex and filopodia tips using the polygon and oval tools to obtain the mean fluorescence intensities of each. For filoTips, filopodia number per cell was counted manually and filopodia lengths were measured using the line tool along the filopodia shaft in ImageJ. For filoSkeleton, filopodia number and length measurements by filoSkeleton were compared to those by FiloQuant. Obvious debris that could be called filopodia and multiple cells in the same frame were cropped out for FiloQuant, and single image analysis mode used to adjust the cell edge parameter (ranging from six to 20) for every cell individually to obtain the most accurate possible FiloQuant measurements for comparison with filoSkeleton. Two-sided Pearson correlation coefficients were calculated using the scipy.stats.pearsonr library in Python. Significance was accepted at a *P*-value of less than 0.05.

### Data acquisition

#### filoTips

##### Default model

*D. discoideum* cells were grown at 21°C on plastic dishes in HL5 glucose medium (Formedium) supplemented with 10 kU/ml penicillin G and 10 μg/ml streptomycin sulfate. Cells were transformed by electroporation, then selected and maintained in 10–20 μg/ml G418 (neomycin resistance, Thermo Fisher Scientific) or 50 μg/ml hygromycin B (Gold Biotechnology), depending on the plasmid. Integrating or extrachromosomal expression plasmids (Arthur et al., 2021; Petersen et al., 2016) were used to express the tip marker DdMyo7 tagged with various fluorophores (see Table S2 for plasmid numbers and fluorophores used). Cells were plated in 35-mm, no. 1.5 coverslip imaging dishes (MatTek or CellVis) at a density of ∼10^5^ cells/cm^2^ and allowed to adhere for 10 min. Cells were rinsed twice in phosphate buffer (16.8 mM sodium/potassium phosphate, pH 6.4) and placed in 1–2 ml of phosphate buffer for 45–75 min, the time window for optimal filopodia formation (Petersen et al., 2016). Cells were then imaged with 63× or 100× Plan Apo oil-immersion objectives (NA 1.4) on a Marianas spinning-disk confocal imaging system based on a Zeiss Axiovert microscope equipped with a Yokogawa CSU-X1, a Photometrics Evolve 512 electron-multiplying CCD camera, a Photometrics HQ2 CCD camera, an ASI MS-2000 stage controller, and laser lines at 488 and 561 nm in a SlideBook 6.0 software environment (Intelligent Imaging Innovations).

##### Transfer learning

COS-7 (American Type Culture Collection, CRL-1651) and U2-OS (American Type Culture Collection, HTB-96) cells were grown in Dulbecco's modified Eagle's medium (DMEM; Thermo Fisher Scientific) with 10% fetal bovine syndrome (FBS; Thermo Fisher Scientific) containing 1% penicillin streptomycin at 37°C with 5% CO_2_. Cells were transfected with either an eGFP-tagged human Myo10 expression plasmid (EGFPN3-hMyoX, Addgene plasmid 47609; deposited by Emmanuel Strehler) or mCherry–Myo10 (made by exchanging eGFP in EGFPN3-hMyoX for mCherry by restriction enzyme cloning) via Lipofectamine 2000 (Invitrogen). For plasmid transfection, cells were seeded on day 1, transfected with 0.25–1 µg DNA using Lipofectamine 2000 (Thermo Fisher Scientific) on day 2, and analyzed on day 3. Cells were plated on 24-well glass-bottomed plates with no. 1.5 coverslips (CellVis) that had been pre-coated with 5 µg/ml fibronectin (Sigma-Aldrich, F1411 or FC010). Live-cell images were acquired on a Nikon Ti2-E microscope equipped with a Crest Optics X-Light V3 spinning-disk, 60× Plan Apo oil-immersion objective (NA 1.4), and captured with a Hamamatsu ORCA-Fusion BT CMOS camera. Samples were illuminated with 800 mW lasers (488 or 561 nm) with GFP or DS Red filters (High Signal to Noise BL Series, Nikon) for 488 or 561 nm excitation (final 0.1099 µm pixel size). Five *z*-stacks of 0.3 µm steps were taken with 50–100 ms exposure and 30–60% laser power, and maximum projections were generated for analysis.

#### filoSkeleton model

U2-OS and HeLa cells were grown in DMEM supplemented with 1% GlutaMAX (Thermo Fisher Scientific), 10% FBS and 1% penicillin streptomycin. U2-OS and HeLa cells were plated on 18 mm no. 1.5 glass coverslips pre-coated with 5 μg/ml fibronectin in 1× PBS. Cells either underwent siRNA silencing, followed by DNA transfection with a mCherry–Myo10 expression plasmid, or were directly transfected with a mCherry–Myo10 expression plasmid. The following siRNAs were used for MYO10 human siRNA oligo duplex (OriGene; locus ID, 4651; SR303060: siRNA_A, 5′-GGTCAGGTATTCACTTACAAGCAGA-3′; siRNA_C, 5′-GGAAAAATACGCTCTCTTCACTTAC-3′). For U2-OS or HeLa cells that underwent silencing, cells were seeded and transfected with siRNAs (10 nM) using lullaby reagent (OzBioscience) via a reverse transfection protocol on day 1. This was followed by transfection with a mCherry–Myo10 plasmid using the FuGENE HD Transfection Reagent (Promega) on day 3, then fixation and image analysis on day 4. U2-OS or HeLa cells that did not undergo silencing were seeded on day 1, transfected with a mCherry–Myo10 plasmid using the FuGENE HD Transfection Reagent on day 2, then fixed and imaged on day 3 or 4. For fixation and imaging, cells were fixed with 4% paraformaldehyde and stained with the anti-MyoX primary antibody (Novus Biologicals, NBP1-87748, 1:1000) to detect Myo10 or the anti-FMNL3 primary antibody (Sigma-Aldrich/Atlas Antibodies, HPA002552, 1:500), followed by staining with Alexa Fluor 647-conjugated secondary antibody (Jackson ImmunoResearch, 711-605-152, 1:400) to visualize filopodia tips. The actin cytoskeleton was visualized by staining with either Alexa Fluor 488–phalloidin or rhodamine–phalloidin (Cytoskeleton, PHDG1-A, PHDR1, 1:200). Images were acquired on a spinning-disk system (Gataca Systems) based on an inverted microscope (Ti-E; Nikon) equipped with a sCMOS camera (Prime 95B; Photometrics), a confocal spinning head (X1; Yokogawa), a 100×1.4 NA Plan-Apo objective lens and a super-resolution module (Live-SR; Gataca systems) based on structured illumination with optical reassignment technique and online processing leading to a two-time resolution improvement (Roth and Heintzmann, 2016). Nine *z*-stacks (0.2 μm each) were acquired and max-projected prior to model training and analysis.

### Hardware and software requirements

filoVision can be run locally with Python or within a Google Colab cloud environment that eliminates any specific software or hardware requirements. No proprietary software is required.

### filoVision dependencies

Dependencies associated with ZeroCostDL4Mic are required and imported by filoVision. Additional libraries include: pandas (The pandas development team, 2024), numpy (Harris et al., 2020), glob (https://docs.python.org/3/library/glob.html), shutil (https://docs.python.org/3/library/shutil.html), OpenCV (Bradski, 2000), math (https://docs.python.org/3/library/math.html), scipy (Virtanen et al., 2020), researchpy (https://github.com/researchpy/researchpy), matplotlib (Hunter, 2007) and seaborn (Waskom, 2021).

### Software and source data

The filoVision GitHub repository (https://github.com/eddin022/filoVision) contains links to the filoTips and filoSkeleton Google Colab notebooks, as well as local Python scripts. Source data and training annotations used to train the default filoTips model are publicly available via links in the Github repository. Data for new users to do a test run is also available on the GitHub page. Updates will be provided directly to the Colab notebooks, and updates to the local scripts will be included on the GitHub repository. Users may copy the notebooks or use the local scripts if they want to make personalized edits. The ImageJ macros used for generating filoVision ground truths and the Python script used to calculate segmentation evaluation metrics can be found on the filoVision GitHub repository. Links to filoVision models, data and ground truths can be found on the GitHub repository as well.

## Supplementary Material



10.1242/joces.261274_sup1 Supplementary information

Table S1. Filopodia Tool Comparison. Table listing several prominent filopodia analysis tools along with their analysis goals, strengths, and limitations for comparison with filoVision. Potential users should look at this table to determine the best analysis tool for their specific use case.

Table S2. filoVision Model Train Test Data Description. Description of data used for filoVision model training and evaluation. There are separate sheets for filoTips model train data, filoTips model test data, filoTips transfer learning train data, filoTips transfer learning test data, training data for the filoSkeleton models, and test data for the filoSkeleton models.

Table S3. filoVision Model Prediction Scores. Prediction scores for all filoVision models described in the study.

Table S4. filoTips Output. An example summary table featuring filoTips cell and filopodia analyses split by sheet.

Table S5. filoSkeleton Output. An example summary table featuring filoSkeleton cell and filopodia analyses split by sheet.

Table S6. Comparison between filoTips models and ground truths. Body IoU scores, body F1-scores, and filopodia counts for 56 individual cells (30 COS-7 and 26 U2-OS) in 52 images analyzed by the default filoTips model and the filoTips model fine tuned to U2-OS and COS-7 cells. IoU scores and F1-scores for cell body are each compared to ground truths.
