# Supplementary material for: filoVision – using deep learning and tip markers to automate filopodia analysis
Source: J Cell Sci. 2024 Feb 27;137(4):jcs261274. doi: 10.1242/jcs.261274 (PMC10941656; doi:10.1242/jcs.261274)
Supplement: Supplementary information [file joces-137-261274-s1.pdf]

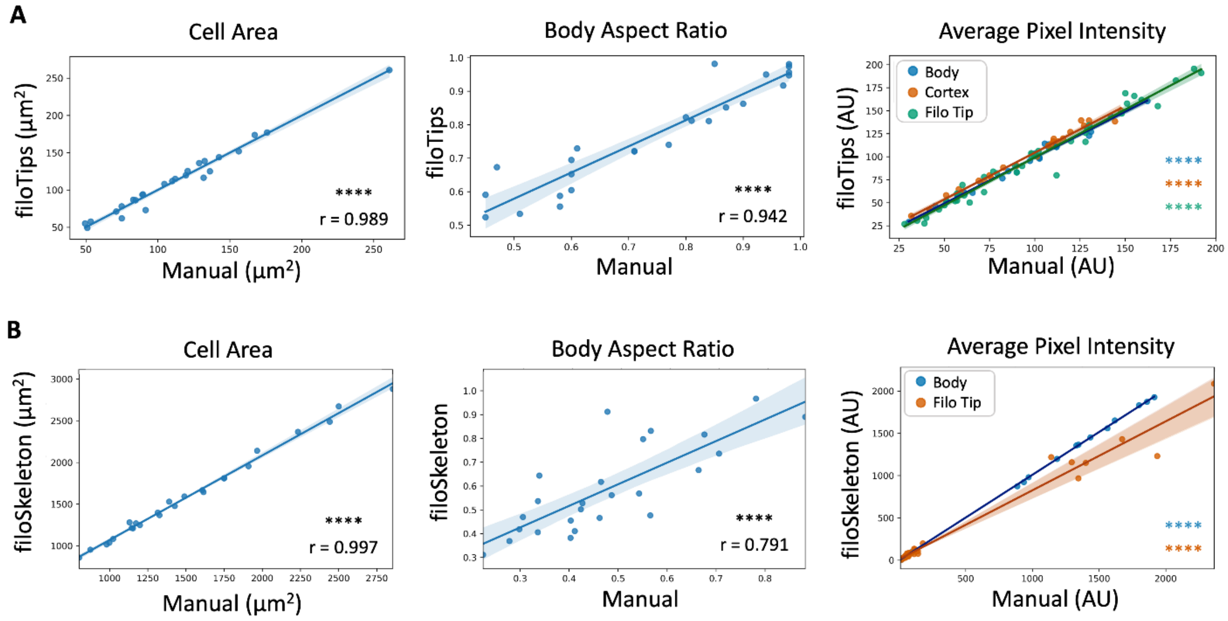

Fig. S1. Comparison of additional measurements between filoTips and filoSkeleton manual analysis. (A) Cell area, body aspect ratio, and average pixel intensity measurements by filoTips and manual analysis. **(B)** Cell area, body aspect ratio, and average pixel intensity measurements by filoSkeleton and manual analysis. Correlation plots for area, aspect ratio, and intensities for body and cortex using 25 random cells. Correlation plots for filopodia tip intensities using 40 random filopodia. Two-sided Pearson correlation, ****: pVal <0.0001.

Journal of Cell Science • Supplementary information

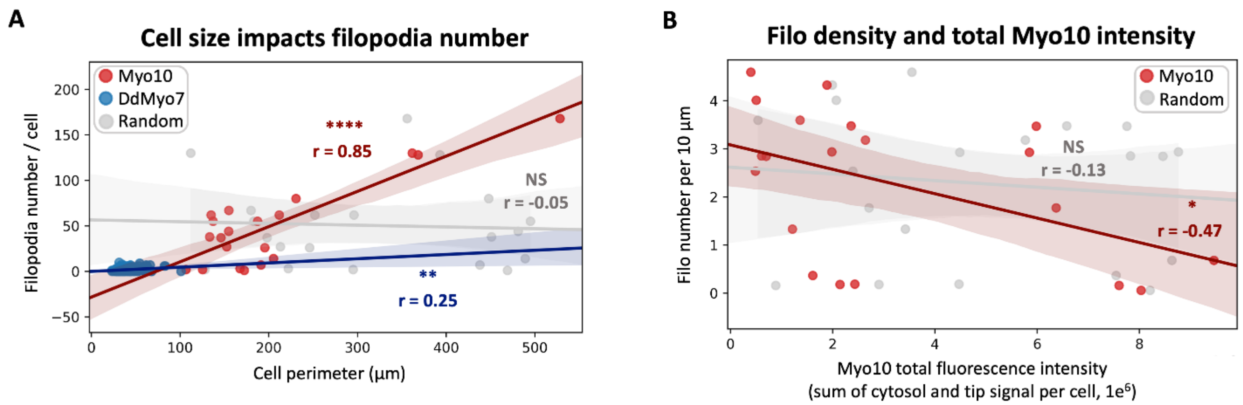

Fig. S2. Correlation between cell size and filopodia number. (A) Correlation of filopodia number / cell and cell perimeter (µm) (PCC) for eGFP-Myo10 expressing U2-OS cells (N:2, n:20, R^2: 0.85, P-Val: $1.97e^{-06}$, red), GFP-DdMyo7 expressing Ddisc cells (N:3, n:153, R^2: 0.25, P-Val: 0.0019, blue), and a random number control array (n:20, R^2: -0.05, P-Val: 0.82, gray). **(B)** Correlation of filopodia density and total (cytosolic and tip) Myo10 intensity (PCC) for eGFP-Myo10 expressing U2-OS cells (N:2, n:20, R^2: -0.47, P-Val: 0.03, red) and a random number control array (n:20, R^2: -0.13, P-Val: 0.59, gray).

Journal of Cell Science • Supplementary information

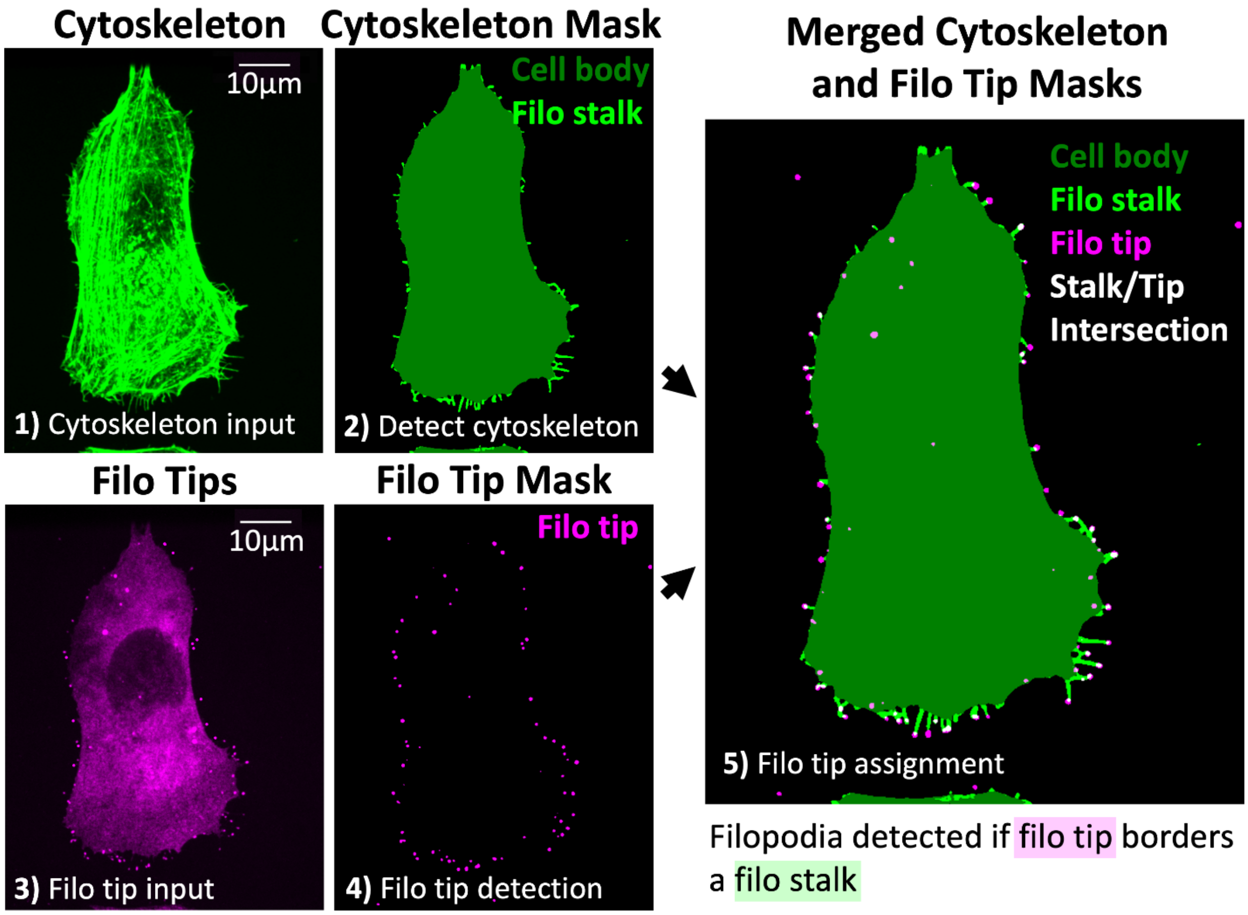

Fig. S3. filoSkeleton filopodia detection and assignment. Image of a fixed U2-OS cell ectopically expressing mCherry-Myo10 (magenta) and stained with Alexa-488 phalloidin (green) is shown. **(1)** The Alexa-488 phalloidin stained image is provided. **(2)** filoSkeleton generates a segmentation of the background (black), cell body (dark green), and filopodia stalks (light green). The cell body and filopodia stalks are identified and recorded. **(3)** Next, the mCherry-Myo10 image is provided to identify filopodia tips. **(4)** filoSkeleton generates a binary segmentation of background and filopodia tips highlighted in black and magenta. **(5)** Filopodia tips are identified if they overlap with, or are within 3 pixels, of a filopodia stalk.

Journal of Cell Science • Supplementary information

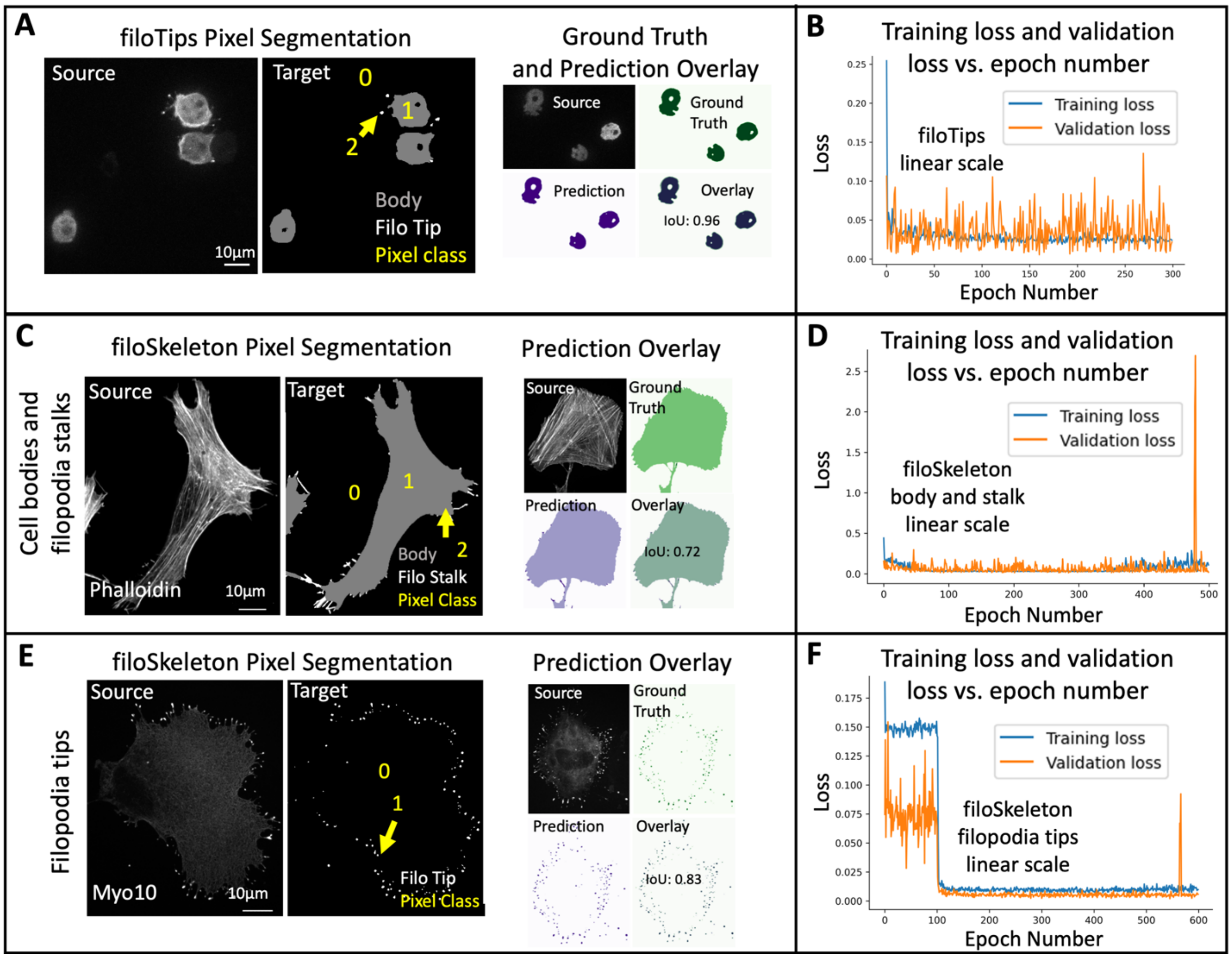

Fig. S4. filoVision model training overview. (A) Representative images of a source and target pair. Source: Live cell images of Ddisc cells expressing GFP-DdMyo7. Target: Segmentation mask of the source image where all pixels have been classified into 3 groups: background-0 (black), body-1 (gray), and filopodia tips-2 (white) assigned pixel class (yellow numbers). The arrow indicates a representative filopodia tip. Representative ground truth and default filoTips model prediction overlay representing a source (white) and ground truth (green) pair along with the trained model prediction (light purple) and an overlay of the model prediction and ground truth (dark blue). In the representative example, the overlay Intersection-over-union (IoU) score was 0.96. **(B)** Plot of training and validation loss by epoch number during training of the filoTips default model. **(C)** Representative images of a labeled cytoskeleton source and target pair. Source: Fixed imaging of a U2-OS cell ectopically expressing eGFP-Myo10 stained with

Journal of Cell Science • Supplementary information

anti-Myo10 antibodies. Target: Segmentation mask of the source image where all pixels have been classified into 3 groups: background-0 (black), body-1 (gray), and filopodia stalks-2 (white) assigned pixel class (yellow numbers). The arrow indicates a representative filopodia stalk. Representative ground truth and filoSkeleton body and stalk model prediction overlays. **(D)** Plot of training and validation loss by epoch number during training of the filoSkeleton body and stalk model. **(E)** Representative images of a labeled filopodia tips source and target pair. Source: Image of a U2-OS cell ectopically expressing eGFP-Myo10 fixed and stained with anti-Myo10 showing labeled filopodia tips. Target: Binary segmentation of the source image where all pixels have been classified into 2 groups: background-0 (black) and filopodia tips-1 (white) and pixel class (yellow numbers). The arrow indicates a representative filopodia tip. Representative ground truth and filoSkeleton filopodia tips model prediction overlays. **(F)** Plot of training and validation loss by epoch number during training of the filoSkeleton filopodia tips model.

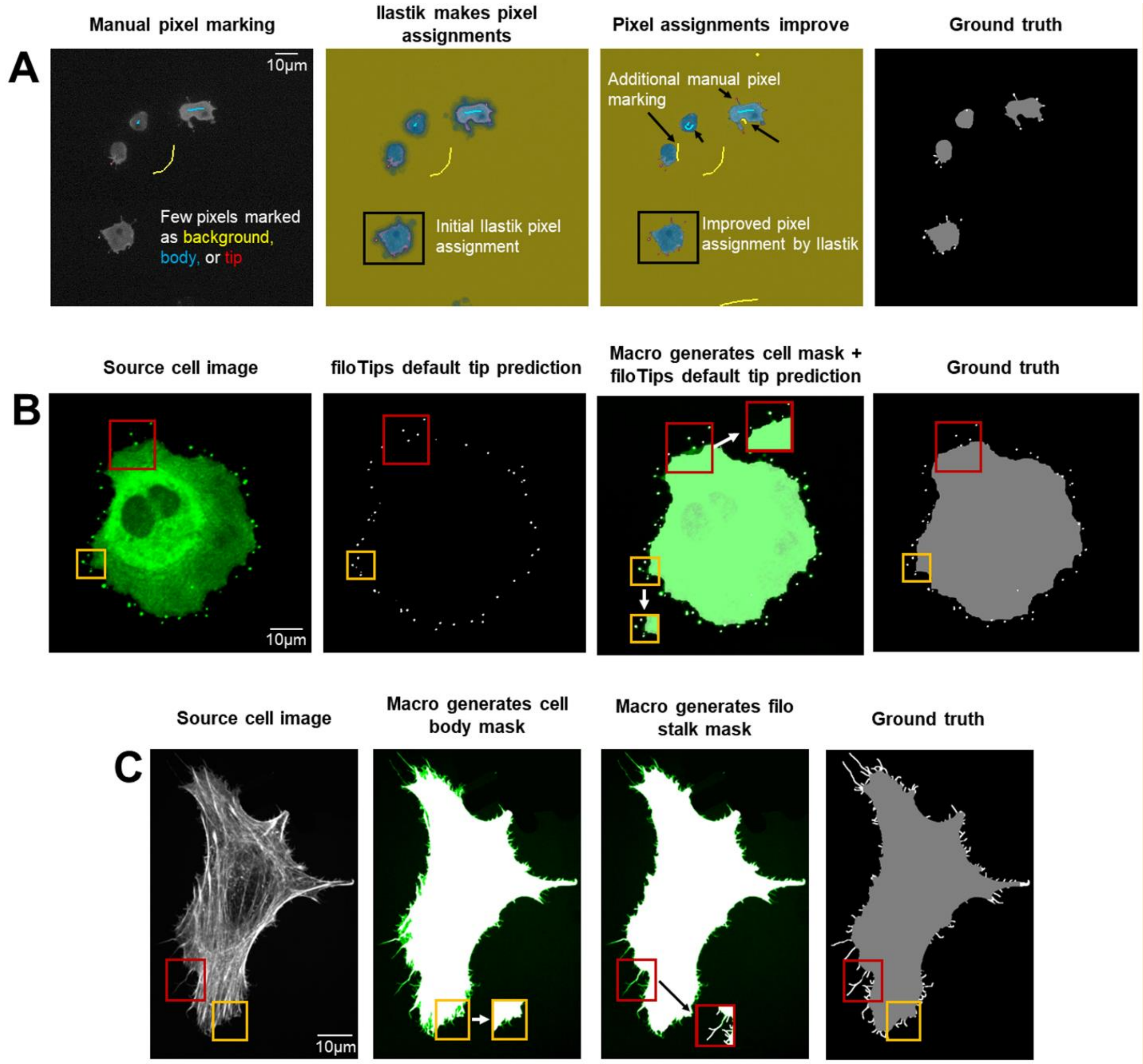

Fig. S5. Ground truth generation methods for filoVision models. (A) Workflow for generation of ground truths using Ilastik. Representative source cells (Ddisc cells expressing DdMyo7) are annotated in Ilastik until pixels are correctly assigned by Ilastik. **(B)** The ImageJ macro "filoTips Ground Truth Generator" generates a mask for the cell body of a representative COS-7 cell expressing Myo10, which is combined with the filoTips default model prediction for filopodia tips to generate ground truths. **(C)** The ImageJ macro "filoSkeleton Body_Stalk Ground Truth generator" generates a mask for the cell body of a representative HeLa cell stained with phalloidin, first for the cell body, then for filopodia stalks. All ground truths generated by these methods were used to train filoVision models.

Journal of Cell Science • Supplementary information

Table S1. Filopodia Tool Comparison. Table listing several prominent filopodia analysis tools along with their analysis goals, strengths, and limitations for comparison with filoVision. Potential users should look at this table to determine the best analysis tool for their specific use case.

Available for download at
https://journals.biologists.com/jcs/article-lookup/doi/10.1242/jcs.261274#supplementary-data

Table S2. filoVision Model Train Test Data Description. Description of data used for filoVision model training and evaluation. There are separate sheets for filoTips model train data, filoTips model test data, filoTips transfer learning train data, filoTips transfer learning test data, training data for the filoSkeleton models, and test data for the filoSkeleton models.

Available for download at
https://journals.biologists.com/jcs/article-lookup/doi/10.1242/jcs.261274#supplementary-data

Table S3. filoVision Model Prediction Scores. Prediction scores for all filoVision models described in the study.

Available for download at
https://journals.biologists.com/jcs/article-lookup/doi/10.1242/jcs.261274#supplementary-data

Table S4. filoTips Output. An example summary table featuring filoTips cell and filopodia analyses split by sheet.

Available for download at
https://journals.biologists.com/jcs/article-lookup/doi/10.1242/jcs.261274#supplementary-data

Journal of Cell Science • Supplementary information

Table S5. filoSkeleton Output. An example summary table featuring filoSkeleton cell and filopodia analyses split by sheet.

Available for download at
https://journals.biologists.com/jcs/article-lookup/doi/10.1242/jcs.261274#supplementary-data

Table S6. Comparison between filoTips models and ground truths. Body IoU scores, body F1-scores, and filopodia counts for 56 individual cells (30 COS-7 and 26 U2-OS) in 52 images analyzed by the default filoTips model and the filoTips model fine tuned to U2-OS and COS-7 cells. IoU scores and F1-scores for cell body are each compared to ground truths.

Available for download at
https://journals.biologists.com/jcs/article-lookup/doi/10.1242/jcs.261274#supplementary-data

Journal of Cell Science • Supplementary information
