# Supplementary material for: filoVision – using deep learning and tip markers to automate filopodia analysis
Source: J Cell Sci. 2024 Feb 27;137(4):jcs261274. doi: 10.1242/jcs.261274 (PMC10941656; doi:10.1242/jcs.261274)
Supplement: Supplementary file 1 [file joces-137-261274_review_history.pdf]

Journal of Cell Science

# filoVision: using deep learning and tip markers to automate filopodia analysis

Casey Eddington, Jessica K. Schwartz and Margaret A. Titus
DOI: 10.1242/jcs.261274

**Editor**: Michael Way

**Review timeline**

| | |
|---|---|
| Original submission: | 26 April 2023 |
| Editorial decision: | 19 May 2023 |
| First revision received: | 8 November 2023 |
| Editorial decision: | 4 December 2023 |
| Second revision received: | 8 January 2024 |
| Accepted: | 9 January 2024 |


---

**Original submission**

First decision letter

MS ID#: JOCES/2023/261274

MS TITLE: filoVision: using deep learning and tip markers to automate filopodia analysis

AUTHORS: Casey Eddington and Margaret A. Titus
ARTICLE TYPE: Tools and Resources

We have now reached a decision on the above manuscript.

To see the reviewers' reports and a copy of this decision letter, please go to: https://submit-jcs.biologists.organd click on the 'Manuscripts with Decisions' queue in the Author Area. (Corresponding author only has access to reviews.)

As you will see, the paper received quite mix reviews and reviewers raise a number of substantial criticisms that prevent me from accepting the paper at this stage. I think they have raised some good points that will definitely improve the study and make the software more user friendly. On the whole they have indicated that a revised version might prove acceptable, if you can address their concerns. If you think that you can deal satisfactorily with the criticisms on revision, I would be pleased to see a revised manuscript. We would then return it to the reviewers.

Please ensure that you clearly highlight all changes made in the revised manuscript. Please avoid using 'Tracked changes' in Word files as these are lost in PDF conversion.

I should be grateful if you would also provide a point-by-point response detailing how you have dealt with the points raised by the reviewers in the 'Response to Reviewers' box. Please attend to all of the reviewers' comments. If you do not agree with any of their criticisms or suggestions please explain clearly why this is so.

Reviewer 1

*Advance summary and potential significance to field*

© 2024. Published by The Company of Biologists under the terms of the Creative Commons Attribution License (https://creativecommons.org/licenses/by/4.0/). 

In this study, Eddington and colleagues describe an image analysis pipeline for automatically counting and measuring filopodia in cells from 2D images. The process requires cells to be labeled with either a filopodia tip marker or both a filopodia tip marker and an actin stain. The pipeline operates on two Jupiter notebooks and uses Google Colab as a backend. The filopodia detection is handled by semantic segmentation Unet models trained using the ZeroCostDL4Mic platform.
This pipeline will likely be helpful for research groups heavily focusing on filopodia studies.

*Comments for the author*

However, before recommending it for publication, the authors should consider the following points.
When should one utilize filoVision?
It would be crucial to illustrate potential scenarios where filoVision proves beneficial.
When is it necessary for users to retrain the models and how generalizable are the models provided by the authors? The authors rightly suggest that users may need to retrain their own models to analyze their specific image sets. However, one may wonder about the duration required to generate these training datasets. Given the number of images the authors use to train their models, it could be more time-efficient to perform the analysis manually or use alternative methods in certain instances. Could transfer learning be employed to fine-tune the models provided by the authors and adapt them to individual image sets, thereby reducing the number of images necessary for training? This approach could leverage existing knowledge and save significant time and resources. A clear understanding of these aspects would enable users to implement filoVision in their research effectively.
Filovision heavily relies on Google Colab, a commercial product outside their control. Can filovision be easily adapted to run on other platforms? Or locally?
Why not use a single multi-label Unet model (with four classes) trained on RGB images in filoSkeleton?

Performance:
I recommend the authors use the F1 and panoptic quality scores to assess their segmentation and not only the Intersection over union metric, especially for the filopodia tip and filopodia shaft classes.
I recommend the authors show the correlation between their manual analyses and their automated analyses, not just the overall measurements (Fig 4, 5, and 8).
How does filoVision perform when filopodia are broken (fragmented actin stain) or crossed? Or when the filopodia density is very high?
The text of the manuscript could be greatly improved.
The introduction could introduce additional tools developed to analyze and detect filopodia (for instance, u-shape 3D, CellGeo). Several pipelines already employ deep learning, and these could also be described.
Many of the technical details regarding how the models were trained could be moved from the results to the method section.
It is significant that the authors provide their analysis pipeline in the form of Jupiter Notebook so that it can easily be used and modified by others. I recommend the author make their training datasets available and findable using, for instance, initiatives such as the Biomage model zoo.

Minor:
The authors wrote: “Fluorescent tip markers allow one to specifically identify filopodia” This is not entirely correct as several tip markers, including MYO10 also decorate stress fibers, especially in cells over-expression of a fluorescently-tagged MYO10 construct.
The multi-labeled Unet notebook used by the authors was released together with the DeepBacs paper (by C Spahn · 2022). The authors could consider citing it.

Reviewer 2

*Advance summary and potential significance to field*

Automated segmentation and analysis of cell images is increasingly common, but identifying and quantifying elongated objects such as filopodia are more difficult with current tools, which may rely on thresholding approaches rather than machine learning-based segmentation. The authors try

© 2024. Published by The Company of Biologists under the terms of the Creative Commons Attribution License (https://creativecommons.org/licenses/by/4.0/).

to address this by developing an accessible and reproducible image analysis pipeline to identify, count and measure filopodia using filopodia tip markers, with or without concurrent cytoskeleton labelling. The pipeline, filoVision, is based on first using deep learning networks available through an existing platform, ZeroCostDL4Mic, to train a deep learning model to segment filopodia tips and cell bodies. FiloVision then uses the model output to assign filopodia tips to cells and quantify filopodia numbers and lengths. The authors demonstrate the effectiveness of the pipeline on two cell types, D. discoideum and U2-OS, with labelled filopodia tips, showing generally accurate identification of filopodia.

*Comments for the author*

Though the tool will likely be useful to many users, the advance of this paper seems limited as the core novel component of the pipeline – training the deep learning model to segment filopodia – will need to be repeated afresh for each user (manual annotation of a training dataset, then using the ZeroCostDL4Mic platform), whereas presenting a 'generalist' model, trained on diverse images of numerous filopodia types, would be a significantly useful tool for the field. Also, the downstream quantification steps are similar to previous filopodia analysis pipelines (as listed in the introduction) and seem not to be set up for tracking filopodia over time in live imaging datasets. The usefulness of the model could have been demonstrated more strongly, for example, by applying it to a wider variety of cell types, or using it to answer a biological question.

The approach of exploiting filopodia tip markers to identify filopodia will be useful, especially as some cytoskeleton and cell membrane probes are known to not reliably mark the far tip, but has its limitations.
Because spots of tip protein are segmented individually then assigned to the nearest cell as filopodia, this approach is vulnerable to errors when tip proteins are dim, appear as spots in the filopodia shaft, or when cells are close together tips may be assigned to the wrong cell (e.g. cells 1-2 in Fig 3A). The authors show that this approach gives accurate measurements for filopodia numbers and length, but more complex morphological parameters (e.g. sweeping motions over time) would be hard to include as the filopodium shape and base position are not specifically measured. In cells with filopodia tips marked by overexpressed tip proteins such as Myosin X or DdMyo7 overexpression artefacts are likely.

Overall, this tool would be useful to a range of users, but has relatively narrow constraints on imaging (sparse cells with straight filopodia labelled by a tip marker), with some errors and relatively limited advance (users having to train their own model), we do not recommend in the current form.

Other comments

The paper in general is well written and the figures are clear, but some details are not explained:

Introduction

Though the authors point out the need for a deep learning-based tool for filopodia segmentation, existing deep learning tools (e.g. generalist cell segmentation models) that might be applied to this task are not discussed or benchmarked against.

Results

(pg 7 and elsewhere) do the authors mean mNeonGreen instead of mNeon?

(pg 9) Explain how the cortex is defined, since it is not included in the segmentation mask.

(pg 12) Explain the use of the OpenCV detectContours function to define cell bodies as this wasn't explained in the filoTips section.

(pg 12) More detail on assignment of tips to cells – "if the tips are adjacent to a filopodia stalk" – how close?

© 2024. Published by The Company of Biologists under the terms of the Creative Commons Attribution License (https://creativecommons.org/licenses/by/4.0/).

(pg 13) Explain what is meant by “manually enhance the dim filopodia tips in imageJ”.

Figures

Graphical abstract: green and red colour scheme should be made colourblind-friendly.

Fig3: Explain how the annotations for cortex and spacing are assigned. Nothing appears to be assigned to the blue regions in Fig 3B.

Fig4: Explain what statistical tests have been used, e.g. in three-way comparisons, is it pairwise?

Reviewer 3

*Advance summary and potential significance to field*

The authors present filoVision, a collection of deep learning-based image analysis platform for the automated detection and quantification of filopodia in two-dimensional fluorescence microscopy images. The authors provide an overview of the training and testing of the models underpinning their platform, along with a detailed breakdown of the outputs their platform provides. Results produced using their analysis platform, for live Ddisc cells expressing GFP-DdMyo7 and fixed U2-OS cells labeled with phalloidin and anti-Myo10, are compared to manually-annotated ground truths. The implementation of the platform as Google Colab notebooks makes sharing of code trivial. While such a tool would undoubtedly be useful to the broader cell biology community, I have a number of concerns with the manuscript that need addressing before it is suitable for publication.

*Comments for the author*

First of all, there is a lot of content in the manuscript that falls under the heading of "documentation" and could probably be moved to an online wiki (such as that on the Github repo) - the manuscript reads a little too much like a user manual in places. Specific examples are provided below.
There is also not enough information provided on the nature of the data used for the training and testing of the deep learning models, nor is much information provided with regard to how the models were trained, why a particular model architecture was used, etc. There's a general over-reliance on the ZeroCostDL4Mic platform to provide such details. For example, in the section "Training a filoTips model", "detailed training instructions here" provides a link to the ZeroCostDL4Mic landing page, which tells the reader absolutely nothing about how filoTips was trained. In the same section, "The default filoTips model was trained using the "U-Net (2D) multilabel" notebook on the ZeroCostDL4Mic wikipage" cites the same ZeroCostDL4Mic landing page, when it should cite the paper describing U-Net (https://doi.org/10.1038/s41592-018-0261-2).
Details of how ground truths are generated is also confusing in places, with references to ilastik and ImageJ made at different points in the text and insufficient detail is provided. Again, specific examples of omissions are provided below, but all of this information should be consolidated in the materials and methods section.
I'm also not entirely convinced that two separate notebooks, containing three different models, are entirely necessary? Could the code not be consolidated into a single notebook for ease of use? And while the use of Google Colab makes sharing of code easy, what if I have a very large amount of data I want to analyse, which is not practical to upload via Google Drive - can I export the models and run them elsewhere?
I also think the authors need to further test their platform on a wider variety of datasets to demonstrate the robustness of their platform. At present, the models are only tested on data partitioned from training data sets. For example, why was the filoTips model not tested on the fixed U2-OS cells? The training datasets are very small (only 460 images for filoTips, for example), which may mean the models do not generalise very well to new, previously unseen data.
Similarly, the filoVision platform is not compared to other published tools, several of which are mentioned in the introduction (FiloDetect, filoQuant and Filopodyan). I think this is especially important given that the false positive rate reported in Figure 4B and the false negative rate in Figure 8B are somewhat high - how do these compare to other software tools? Only a limited comparison with SEVEN is provided.

© 2024. Published by The Company of Biologists under the terms of the Creative Commons Attribution License (https://creativecommons.org/licenses/by/4.0/). 

Finally, 8 figures seems excessive - I think all results could be condensed into 2 or three figures at most.
Also, the absence of line numbers makes it difficult to refer to specific points in the text.

Specific Points:

* Introduction
The introduction ends with a rather strange statement about self-driving cars and consumers, when it really should end with a paragraph summarising the key findings of the paper. What is filoVision? How does it differ from other software? What results were achieved with it? Etc.

* Results And Discussion
The first paragraph is largely introductory and could be moved to the end of the previous section. Within the first paragraph, it is mentioned that "filoVision is packaged in a Google Colab cloud environment to make it more accessible and user-friendly, it also provides free high computational power...". While it is true that Google Colab is free to use, there are resource limits (https://research.google.com/colaboratory/faq.html#resource-limits) and getting around those limits requires the purchase of a pro plan (https://colab.research.google.com/signup)

** Training a filoTips model
It is stated that "Wild type and filopodia mutant amoeba (myo7 or vasp null) expressing wild type or non-functional mutant DdMyo7 tagged with a variety of fluorophores, including GFP, mCherry, and mNeon were used" - how many of each were used? A much more detailed breakdown of what data was used for training, validating and testing the model should be provided and ideally, the training data should be uploaded to an archive (like the BioImage Archive).
In the same section, it is mentioned that ilastik was used to generate ground truth data, but only very vague details are provided (along with some concerning statements about manual correction being required - what does this mean exactly?) and there is no mention of ilastik in the materials and methods section. The generation of ground truth segmentations should be detailed in a dedicated section under materials and methods.
"Default data augmentations ... were enabled" - what exactly does this mean? The authors should explicitly state exactly what data augmentation layers were used during training. This should appear in a dedicated section on model training under materials and methods.
"The rest of the training parameters were set to default..." - again, what does this mean? What training parameters are being referred to and what does "default" mean in this context? All training parameters should be detailed in a dedicated section in materials and methods.
In the next paragraph, there is an explanation of "Intersection over Union" - this should appear in materials and methods in a section detailing performance evaluation
Later in the same paragraph, it is stated that "The average IoU for the default filoTips model is 0.76 ± 0.10" - when tested on what? Training data? Unseen test data?
The paragraph finishes with the statement "The final evaluation of filoTips is filopodia tip detection accuracy ... however this is highly dependent on model performance" - I don't understand this statement? The performance of filoTips is highly dependent on the performance of filoTips?!?

** Detecting and analyzing filopodia with filoTips
The section starts with "A trained and deployed filoTips model should now be ready to generate accurate prediction segmentations of cell bodies and filopodia tips in user’s data". This is an odd statement with which to begin a results section. The authors should be trying to persuade us that their model works when trained on their data, not suggesting it might work on different data!
The following statement on spatial separation of cells should come later in a general discussion of the limitations of the platform, although I note that this criteria is not tested at any point - how separated do cells have to be in order for filoTips to work?
Following this, it is stated that "various parameters such as area, aspect ratio, and pixel intensity are extracted" - how? This should be explained in materials & Methods in a dedicated section on implementation.
The authors then state that filopodia tips are assigned to the nearest cell body, but again, no explanation of exactly how this is achieved is provided. This should be elaborated upon. It should also be explained how reduced cell separation will impact this.
Then "The remaining metrics such as the number of filopodia per cell, length, and tip intensity are extracted", but again, no explanation is provided as to how any of this is achieved. This is

© 2024. Published by The Company of Biologists under the terms of the Creative Commons Attribution License (https://creativecommons.org/licenses/by/4.0/).

particularly relevant given that we are later told that curved filopodia may not be measured accurately.

** Evaluating filoTips' performance
This section begins with a reference to manual measurements made in ImageJ - it's difficult to follow exactly how manual segmentations and manual measurements were made for the purposes of (a) training the model and (b) evaluating the model. This should all be detailed in a dedicated section under materials and methods.
"To be considered a reliable quantitation tool, filoTips measurements should be as similar to manual measurements as possible." - I'm not sure about this statement. First of all, manual measurements can be inaccurate. Secondly, if a machine learning model demonstrates 100% accuracy (or close to it) on a particular dataset, it is very likely that the model has been over-fit to its training data and will not generalise well to previously unseen data.
"Manual correction of the robust stalks was not performed prior to evaluation, but the false positive rate can be reduced by erasing them prior to filoTips analysis as needed" - isn't the whole point of automated image analysis that manual intervention is not required?!? Besides, advising people to selectively manipulate their images prior to analysis seems likely to be bad for reproducibility.
"It detected 4.1 ± 4.5 filopodia per cell, which is similar to a manual count of 3.6 ± 4.4" - what doe the errors here represent? They seem pretty big?
"SEVEN's significantly more conservative estimate results from its parameters being optimized for GFP-DdMyo7 signal and employing a low threshold cut-off (Petersen et al., 2016), making it unable to generalize to other, brighter fluorescent proteins unless specifically tuned each time" - but filoTips has been specifically tuned (trained) to this data, so it's not really a fair comparison? How well does filoTips generalise to other data?
"It shows that when trained on the user's data, filoTips is a reliable tool..." - what if it is not trained on the users data? How reliable is it then? Does the model need to be completely retrained in order for it to be in any way useful?

** Training a filoSkeleton model
"A well-trained model serves as the foundation for accurate filopodia detection, the true performance metric." - what is a "true performance metric"?!?
"The second model takes an image of filopodia tip foci and segments the pixels into two classes: 0-background and 1-filopodia tips" - I'm not sure I understand how this differs from the filoTips model?
"...default filoSkeleton models are provided, but training a custom model is recommended" - I don't understand why the authors keep making these kinds of statements. Why describe the process of generating the default models if it is recommended that they not be used?
Again, the description of the training data used in this section should be moved to materials and methods and expanded upon, along with details of ground truth generation (the ImageJ macro referred to do this should be provided).
"In some cases cells were treated with siRNA for Myo10 to reduce or eliminate filopodia." - the details of this procedure should be provided in materials and methods.
86 U2-OS cells were used to train the model for segmenting cell bodies, but 121 images of were used for training filopodial tip detection? Shouldn't the same multi-channel images be used to train both models?
"95%. The average IoU for the model predicting cell bodies and filopodia stalks was 0.81 ± 0.09 (Fig. 5C), and the average IoU for the model predicting filopodia tips was 0.84 ± 0.02 (Fig. 5D), meeting the set performance standards." - what data was tested here?

** Evaluating filoSkeleton's performance
"The accuracy of filoSkeleton in measuring filopodia was evaluated by comparing its quantitation metrics to manual measurements made using imageJ on a total of 17 images" - that is a very small number of images. I think far more testing is required to convince that this is a useful model.
"Unlike filoTips, filoSkeleton missed 113 out of 810 total filopodia" - so the model that's been trained on more information (actin staining and filopodial tip marker) actually performs worse than the first model (filopodial tip marker)? Why? And if this is the case, should people not just use filoTips?
"...it is possible to rapidly identify which cells are affected by this issue and manually enhance the dim filopodia tips in imageJ... " - again, encouraging people to selectively manipulate their images is a really bad idea.

© 2024. Published by The Company of Biologists under the terms of the Creative Commons Attribution License (https://creativecommons.org/licenses/by/4.0/).

* Conclusion
A large chunk of this text is actually discussion and should be moved to that section.

* Materials & Methods
** Hardware and software requirements
"Although not necessary, it is recommended to use Ilastik and ImageJ for manual annotation and image manipulation tasks" - if it's not necessary, then why is it mentioned in materials and methods?!? And what kind of manual annotation and image manipulation is being referred to here?

** Running filoVision
This doesn't belong in materials and methods - it's a user guide and should be on the github wiki

** Default filoTips model data acquisition
"Various fluorphores" are mentioned - this is far too vague. A detailed list should be provided.

** Default filoSkeleton model data acquisition
"Cells were imaged on a spinning disk microscope (SP3) with 60x and 100x objectives" - more details of the acquisition system are required here.

* Figures
I would suggest combining Figures 1 and 2 into a single figure. I'm not sure Figure 3 adds much and could potentially be removed. Figures 5 and 6 could probably also be combined and Figure 7 removed. Also, it is not made clear in the figures what statistical tests were used.

** Figure 4

*** A: I think enough illustrations have been provided already in Figures 1 - 3. I'm not sure this adds a great deal.

*** B: Error rates should be unambiguously defined in materials and methods. It is not clear to me whether the error rates are being calculated on the basis of the correct number of filopodia being detected in a dataset, or the correct number of filopodia per cell being detected.

** Figure 5

*** A: Again, I'm not sure this adds much, but regardless, isn't the source here phalloidin?

** Figure 8

*** B - G: It would appear the authors have pooled together the results of an analysis of WT U2-OS cells and siRNA-treated cells (and HeLa cells?) - the analysis of these two different populations should be displayed separately.

Dave Barry Francis Crick Institute

---

**First revision**

Author response to reviewers' comments

We would like to thank the reviewers for their though4ul comments which have resulted in a significantly improved manuscript. First, we would like to respond to comments shared by at least two reviewers. Then we will respond to more specific comments by each reviewer individually.

Note that the manuscript has been altered significantly in response to all the comments. See main changes below:

© 2024. Published by The Company of Biologists under the terms of the Creative Commons Attribution License (https://creativecommons.org/licenses/by/4.0/).

**Manuscript Structure:**

It was pointed out that the manuscript read like a user manual at times. We removed these instances from the manuscript. The results now focus on comparing filoTips measurements to manual measurements, tuning the filoTips model trained on amoeboid cells to U2-OS and COS-7 cells, demonstrating the types of biological questions that can be answered with filoVision, and finally comparing filoSkeleton to FiloQuant to demonstrate its reliability as an analysis tool. The discussion now has a “limitations and other considerations” section. Details originally in the results section regarding model training and how filoVision extracts information from images have been moved to the materials and methods where we agree it is more appropriate.

**New results sec2ons:**

1) transfer learning
2) types of biological questions that can be answered

It was recommended that we focus more on transfer learning. We now highlight that a lab member with no prior deep learning knowledge was able to generate ground truths and tune our filoTips model trained on amoeboid cells to U2-OS and COS-7 cells within 48 hours using the ZeroCostDL4Mic 2D mulC-label U-net notebook with minimal guidance. The amoeboid model was surprisingly able to reliably predict U2-OS and COS-7 filopodia Cps, but oXen underestimated the relatively dim U2- OS/COS-7 cell edges prior to finetuning so we used transfer learning to improve this aspect of the model for the mammalian cells.

A new section describing some of the biological questions that can be answered with filoVision has been added to the manuscript. In this section, we compared the relationship between Myo10 and DdMyo7 expression and filopodia formation, detailing key observational differences. We also highlight that we were able to analyze 1,008 filopodia over 20 U2-OS cells transiently expressing eGFP-Myo10 or mCherry-Myo10 and 280 filopodia over 153 Ddisc DdMyo7-null cells expressing GFP-DdMyo7 in less than an hour, which otherwise couldn’t be done using existing methods. We believe this helps demonstrate the advantage of using filoVision.

**Figures**

It was recommended that correlation plots be used when comparing filoVision measurements, and that the figures should be condensed and more concise. We have implemented these changes, and agree they are a significant improvement.

**---Comments shared by at least 2 reviewers---**

**When should one u2lize filoVision? It would be crucial to illustrate poten2al scenarios where filoVision proves beneficial.**

filoVision should be used by researchers studying filopodia and rely on the use of widely used tip markers such as Myo10, VASP and formin to identify these slender cellular protrusions. filoVision’s use of deep learning models enables high reproducibility even between different lab members, and it minimizes the need for human intervention which significantly reduces human biases involved in measuring filopodia. filoVision enables rapid and efficient analysis, eliminating the requirement to crop out individual cells and enabling multi-cell analysis in a single frame. Thus, investigators who have multiple datasets and conditions to compare requiring analysis of hundreds or even thousands of filopodia, filoVision will streamline their workflow and enables acquisition of a large amount of data in a relatively short period of time. This will be particularly true if the investigator uses tip markers without an actin or membrane label to visualize filopodia because as far as we know an automated workflow that analyzes tip markers alone without a separate cytoskeletal label isn’t yet widely available. It should also be used by investigators needing to quantify more than filopodia number, as it offers a comprehensive data extraction approach. See text and Rebuttal Table 1, below.

These points are now made explicitly in the text, notably in the final “Conclusions” paragraph

Lines 118-120:

*Thus, filoVision was developed to address the lack of an automated workflow for measuring tip-marked*
*filopodia in diverse cell types like amoeba.*

© 2024. Published by The Company of Biologists under the terms of the Creative Commons Attribution License (https://creativecommons.org/licenses/by/4.0/).

Lines 364-368:
*filoVision’s ability to analyze multiple cells simultaneously within the same image allows the user to save time spent cropping images or manually tuning parameters to each cell, which can cause filopodia analysis to become tedious. filoTips was shown to analyze hundreds of U2-OS or Ddisc filopodia in less than an hour, which wouldn’t be possible using existing methods.*

Lines 430-447:
***Conclusion***
*Filopodia tools have been primarily developed for the analysis of actin or membrane-labeled mammalian cells that are relatively large (30 - 100 µm diameter) and as such many aren’t optimized for smaller, less common cell types like amoeba (10 µm diameter). While labeling filopodia tips is common (see examples: Jacquemet et al., 2019b; Kerber and Cheney, 2011; Petersen et al., 2016), to our knowledge an automated tool that uses tip-labeling alone to quantify filopodia doesn’t currently exist. These were the core limitations that motivated the development of filoVision. To address the lack of flexibility for user data or cell type, filoVision takes advantage of the accessible ZeroCostDL4Mic pla]orm’s transfer learning capabilities. It also enables rapid live cell analysis with a lone tip marker when the cytosolic signal is sufficiently high, and this cytosolic fraction can be used to detect and measure the cell body (filoTips). Alternatively, the tip marker can be combined with actin staining to obtain similar measurements, with the benefit of extracting additional filopodia shaa information (filoSkeleton). Currently, filoVision is being expanded to include tracking filopodia tips over time in live- cell experiments and will include the ability to use more than one tip marker for extracting co- localization information about different tip marker proteins, providing the ability to gain even more insight into the role of different filopodia tip proteins and their collaboration during filopodia formation.*

**RebuGal Table 1.** Summary of all measurements extracted by filoVision.

| **All Body Measurements** | **All Tip Measurements** |
|---|---|
| Cell ID | Filopodia ID |
| Cell Body Aspect Ratio | Cell Assignment ID |
| Cell Body Circularity | Tip Intensity (Total Sum) |
| Body Intensity (Total Sum) | Filo Tip Intensity (Mean) |
| Body Intensity (Mean) | Assigned Cell Body Intensity |
| (Mean) | |
| Cortex Intensity (Mean) | Filo Tip Area (µm^2) |
| Leading Edge Intensity (Mean) | Filo/Body (Mean Intensity Ratio) |
| Side & Rear Intensity (Mean) | Filo Length (µm) |
| Cortex/Body (Mean Intensity Ratio) | |
| Leading Edge/Body (Mean Intensity Ratio) | |
| SideRear/Body (Mean Intensity Ratio) | |
| Leading Edge/SideRear (Mean Intensity Ratio) | |
| Filopodia Number | |
| Filopodia Density (Filos/10µm or Filos/1µm) | |
| Average Filopodia Length per Cell(µm) | |
| Cell Area (µm^2) | |
| Cell Perimeter (µm) | |

**Demonstrate usefulness with a biological ques2on**
We agree with the reviewers that it is important to show how filoVision can be used to address a biological question. Indeed, we considered doing so in the early stages of writing but in the end decided against this in favor of focusing on describing filoVision itself. The revised manuscript now includes a section describing some biologically useful measurements provided by filoVision and the types of questions that can be answered.

A new section entitled “***Rela-onship between Myo10 and DdMyo7 expression and filopodia forma-on***” is now included (starting on line 232). It presents an analysis on the relationship between filopodia formation and filopodial myosin expression level and distribution for both the amoeboid DdMyo7 and mammalian Myo10. Such an analysis was done to further probe for

© 2024. Published by The Company of Biologists under the terms of the Creative Commons Attribution License (https://creativecommons.org/licenses/by/4.0/).

mechanistic similarities or differences between the two filopodial myosins.

First we show that for mammalian cells there is a surprising negative correlation between filopodia number and cytosolic levels of Myo10 while no correlation at all is seen for Ddisc cells (new Fig 4A,B - shown below).

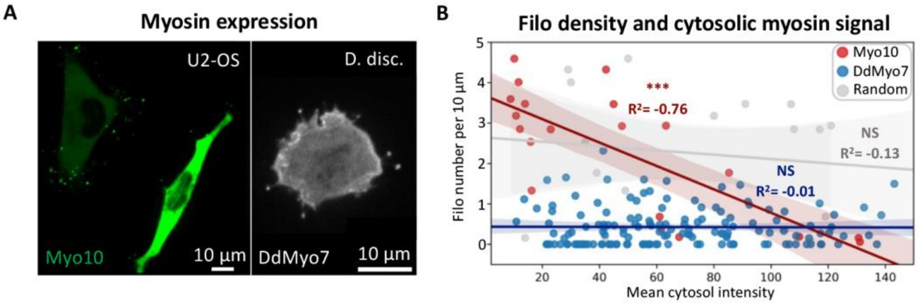


**Figure 4. Correlation of ectopic Myo10 and DdMyo7 expression levels with filopodia formation. (A)** Representative images of U2-OS cells transiently expressing eGFP- Myo10 and a Ddisc cell expressing eGFP-DdMyo7. **(B)** Correlation of filopodia density and cytosolic myosin signal (PCC) for Myo10 (N:2, n:20, $R^2$: -0.76, P-Val: 0.00011, red), DdMyo7 (N:3, n:153, $R^2$: -0.01, P-Val: 0.91, blue), and a random number control array (n:20, $R^2$: -0.13, P-Val: 0.58, gray). NS = not significant

We also compare the cortical enrichment of DdMyo7 and Myo10 expressed in Ddisc and U2OS cells and show a disCnct difference between the two, with the amoeboid myosin DdMyo7 showing distinctive cortical enrichment compared to that of the mammalian myosin Myo10 (new Fig 4C - shown below).

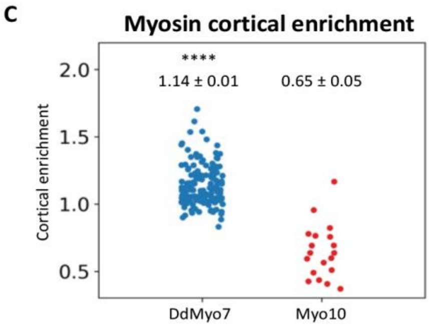


**Figure 4C.** Cortical enrichment measurements for DdMyo7 (N:3, n:153, mean: 1.14, standard error: 0.01, blue) and Myo10 (N:2, n:20, mean: 0.65, standard error: 0.05, red). Statistics: Two-sided student's T-test, P-Val: 0.

There have been indications in the literature that Myo10 tip accumulation may correlate with filopodia length so we asked if increasing filopodial myosin tip intensity is directly correlated with filopodia length in mammalian and amoeboid cells. The results indicate that there is a weak correlation for Myo10, consistent with recent work from the Tyska lab (Fitz et al, 2023, Dev Cell) but no such correlation is seen for DdMyo7 (new Fig 4D - shown below).

© 2024. Published by The Company of Biologists under the terms of the Creative Commons Attribution License (https://creativecommons.org/licenses/by/4.0/).

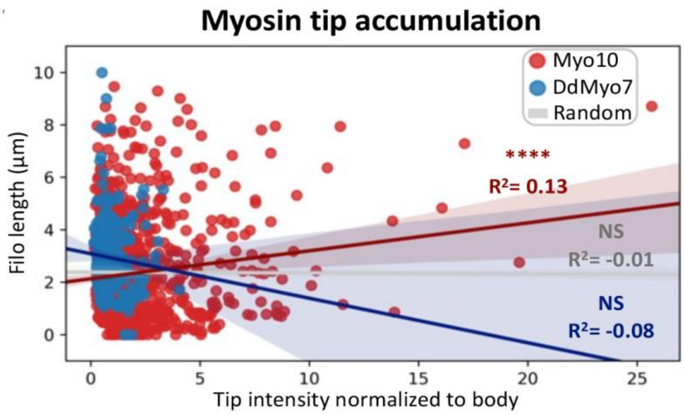


**Figure 4D.** Correlation between myosin tip signal normalized to body and filopodia length (PCC) for Myo10 (N:2, n:1008, $R^2$: 0.13, P-Val: 6.1e$^{-05}$, red), DdMyo7 (N:3, n:280, $R^2$: -0.08, P-Val: 0.21, blue), and a random number control array (n:1008, $R^2$: -0.01, P- Val: 0.71, gray).

Finally, since cell size can vary greatly this can have an impact on filopodia counts, something that many researchers may not account for. The relationship between cell size and filopodia number in mammalian and amoeboid cells was determined and it was found, as one might expect, that the number of filopodia per cell is increased as the size of a mammalian cell increases. The same is true for Ddisc cells but the impact of size on filopodia number is not a large (new Supp Fig 2A - shown below). Since information about the cell body is also extracted by filoVision, filopodia numbers can be normalized to cell perimeter providing an average filopodia density metric (filos / 10µm). Considering cell size can strongly impact filopodia number, measuring filopodia density could be quite useful depending on the research goal.

We further investigated the relationship between Myo10 expression level and filopodia number in U2-OS cells, this time taking into account the total sum of cytosolic and tip localized Myo10. Surprisingly, a negative correlation is still observed(new Supp Fig 2B - shown below), suggesting that a large population of Myo10 may be autoinhibited in these cells. Alternatively, excess Myo10 could saturate the filopodia machinery, as suggested by recent work from the Rock lab (hfps://doi.org/10.7554/eLife.90603.1) showing that increasing filopodia tip intensity results in a plateau of filopodia length.

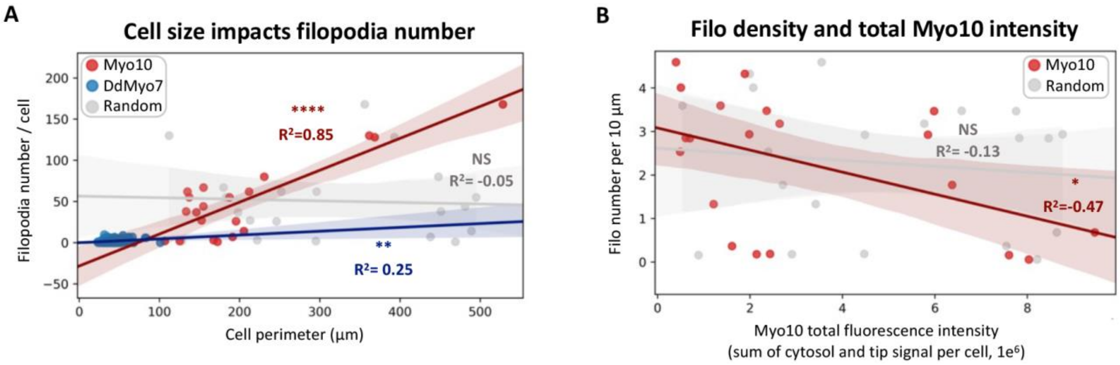


**Figure S2. (A)** Correlation of filopodia number / cell and cell perimeter (µm) (PCC) for eGFP- Myo10 expressing U2-OS cells (N:2, n:20, $R^2$: 0.85, P-Val: 1.97e$^{-06}$, red), eGFP-DdMyo7 expressing Ddisc cells (N:3, n:153, $R^2$: 0.25, P-Val: 0.0019, blue), and a random number control array (n:20, $R^2$: -0.05, P-Val: 0.82, gray). **(B)** Correlation of filopodia density and total (cytosolic and tip) Myo10 intensity (PCC) for eGFP-Myo10 expressing U2-OS cells (N:2, n:20, $R^2$: -0.47, P- Val: 0.03, red) and a random number control array (n:20, $R^2$: -0.13, P-Val: 0.59, gray).

**How generalizable are the models and how difficult is it to finetune filoVision to user data?**

© 2024. Published by The Company of Biologists under the terms of the Creative Commons Attribution License (https://creativecommons.org/licenses/by/4.0/).

**Could transfer learning be employed to finetune the models provided by the authors, reducing the number of images needed for training?**

The reviewers raise an important point here and making filoVision flexible enough for users working with a range of cells of differing morphology and size has been one of our long-term goals. This would require training a general model capable of analyzing all cell types meaning that the scale of the training dataset would need to be much larger than we are able to acquire on our own at this point (we did request data from other labs, but were unsuccessful in obtaining sufficient amounts to pursue a goal of a generalized model).

Therefore, we instead chose to further emphasize how accessible transfer learning is with the ZerotiostDL4Mic pla4orm and that it can be done in as lifle as 1-3 days. In fact, a lab member with no prior deep learning knowledge was able to generate ground truths and finetune the default filoTips model with minimal guidance within 48 hours. Additionally, we ask that all future users upload their own datasets and ground truths to work towards the goal of achieving a more generalized model in the future.

The ability for users to readily tune our models for their own datasets by transfer learning is now described in the text. A new section entitled "***Fine tuning filoTips to diverse cell types***" is now included (starting on line 181). It should also be noted that in our experience, the default filoTips model trained on amoeboid cells reliably predicted U2-OS and COS-7 filopodia tips (see new Table S6 and below, Response to Reviewer #3), needing only minor finetuning to correctly predict the relatively dim U2- OS/COS-7 cell edges.

**When should users train their own model?:**

Our models are publicly available (links on the filoVision GitHub repository) and users should start by analyzing a sample dataset with them. Considering the black-box nature of deep learning, it's difficult to know exactly how well our models will work for everyone, but it should be noted that we were pleasantly surprised with how well our default model was able to generalize to U2-OS/COS-7 cells. As mentioned above, our models also worked reliably for the small number of images we did receive from other labs. UlCmately, we still recommend users utilize accessible transfer learning via ZerotiostDL4Mic for analysis tuned to specific user data for the most accurate future measurements possible. We believe this is a good tradeoff for finetuned automation for users who frequently analyze hundreds or thousands of tip-marked filopodia.

Lines 182-188

*A primary objective of filoTips is to prioritize flexibility so that users can fine tune the tool to their own unique datasets and cell types. filoTips models are publicly available (see GitHub repository), in fact filoTips will ask the user if they want to use the default model, and if so, users will automatically have instant access to it without additional steps. They should feel free to test a small representative dataset and use the default model if they are happy with the results. However, we recommend users tune our models to their own data via transfer learning for the most accurate measurements possible.*

**Would it be more 2me-efficient to do a manual analysis or use another tool?**

A manual analysis might be more time-efficient if the user has a small dataset and doesn't routinely analyze filopodia. If the cells are labeled with phalloidin, a different tool like FiloQuant might be more time-efficient than filoSkeleton if the user has a small dataset or doesn't have frequent filopodia analysis plans. However, we would encourage potential users to spend less than an hour trying our models with their data before making these decisions. If frequent tip-marked filopodia analysis is planned, we believe the initial time investment finetuning our models is worth future automated analysis tuned to the user's data. We have seen firsthand the benefit of being able to analyze hundreds or even thousands of filopodia in less than an hour with filoVison.

Lines 416-427

*Many filopodia analysis tools have been developed with a specific purpose in mind, therefore it's crucial to consider the analysis goals of both the user and the tool. filoVision is an excellent choice if the potential user routinely analyzes hundreds or even thousands of tip-marked filopodia. Its advantage becomes even more apparent when additional cell or tip protein signal information is required, or if speed, reproducibility, and automation are top priorities. However, there are also scenarios when filoVision may not be the ideal choice. For those with smaller datasets or infrequent filopodia analysis needs, manual methods, or a tool like FiloQuant may be more efficient (Jacquemet et al., 2017). Also, if the user lacks tip-labeled data, FiloQuant or*

© 2024. Published by The Company of Biologists under the terms of the Creative Commons Attribution License (https://creativecommons.org/licenses/by/4.0/).

*Filopodyan (Urbančič et al., 2017) is a beier fit depending on the cell marker. If 3D image analysis is required, U-shape 3D (Driscoll et al., 2019) should be considered instead.*

**filoVision should be adapted for local runs, datasets and annota2ons should be made available.**
The reviewers make an excellent point, enabling local runs would be more convenient for many users. filoVision has now been adapted for local runs via python scripts.

It was always the plan to make models, datasets, and ground truths publicly available and Reviewer 1 suggested a great pla4orm, Bioimage.io for this so we have uploaded all necessary materials there. One model has been accepted by zenodo (data storage for Bioimage.io) and the others are currently under review and should be accepted soon. The datasets and ground truths consisting of Ddisc, U2-OS, and COS-7 cells have been uploaded to zenodo and are currently under review. The datasets and ground truths used for training our filoSkeleton models haven't been uploaded yet because this data belongs to our collaborators (filoSkeleton was initially conceived during a collaboration). Because they are actively engaged in the project this data is associated with, we will wait to upload the dataset and ground truths until they publish their findings. Until then, a small example test dataset for filoSkeleton can be found on the filoVision GitHub repo. One model has been accepted, but everything else is still under review. Because of that, we will provide links to everything on the filoVision GitHub as they become available.

Lines 716-725:
*Data and soaware availability*
*The filoVision GitHub repository (hips://github.com/eddin022/filoVision) contains links to the filoTips and filoSkeleton Google Colab notebooks, as well as local python scripts. Data for new users to do a test run is also available on the GitHub page. Updates will be provided directly to the Colab notebooks, and update to the local scripts will be included on the GitHub repository. User's may copy the notebooks, or use the local scripts if they want to make personalized edits. The ImageJ macros used for generating filoVision ground truths and the Python script used to calculate segmentation evaluation metrics can be found on the filoVision GitHub repository. Links to filoVision models, data, and ground truths can be found on the GitHub repository as well.*

**What is the benefit of having two separate notebooks and why not use one model for filoSkeleton instead of two?**
It is possible to combine the two notebooks. However, we thought it would be less confusing to users if they were separated based on visualization target and allowed us to include specific instructions unique to either filoTips or filoSkeleton. It's also possible to use one model for filoSkeleton instead of two. However, initial trials were performed using two models vs. one model with four classes and those initial trials suggested the two-model approach more accurately predicted filopodia. Therefore, we considered that the two-model approach was best for users.

**filoVision should be compared to more tools**
This is another excellent point by the reviewers. filoTips enables filopodia analysis using a tip marker alone without the need of a cytoskeletal label. As far as we know, all other existing tools require either a membrane marker or actin label to visualize filopodia. Because of this, filoTips was compared to manual measurements made in ImageJ as done in the initial submission. However, it is possible to compare filoSkeleton to FiloQuant because both involve using an actin label to measure filopodia and FiloQuant is currently a leading filopodia analysis tool. This was done (see "filoSkeleton: filopodia analysis using tip markers coupled with actin labeling" starting at line 324) and the text updated to include a paragraph in the introduction briefly comparing prominent workflows.

Lines 104 - 120:
*Many tools have been developed to measure filopodia, demonstrating that there is high demand for workflows that quantify filopodia production. These tools are typically targeted towards a certain cell type and/or filopodia visualization method, and each have their own strengths and limitations (Table S1; Barry et al., 2015; Driscoll et al., 2019; Jacquemet et al., 2017; Mousavi et al., 2020; Nilufar et al., 2013; Tsygankov et al., 2014; Urbančič et al., 2017). For example, FiloQuant and Filopodyan each have different visualization targets. (Jacquemet et al., 2017; Urbančič et al., 2017). FiloQuant uses an actin label to identify and measure filopodia stalks protruding from a cell body, whereas Filopodyan uses a membrane marker with the option of*

© 2024. Published by The Company of Biologists under the terms of the Creative Commons Attribution License (https://creativecommons.org/licenses/by/4.0/).

*including a tip marker to measure filopodia and their dynamics over time. To the best of our knowledge, a tool specifically designed to measure filopodia using a tip marker alone or in combination with an actin label doesn't exist, yet many use tip markers in their filopodia analysis workflow (Petersen et al., 2016; Jacquemet et al., 2019b). Furthermore, it can be difficult to find tools adaptable for diverse cell types like amoeba, likely due to most tools being designed for working with mammalian cells. Thus, filoVision was developed to address the lack of an automated workflow for measuring tip-marked filopodia in diverse cell types like amoeba.*

Additionally, we have added a supplementary table (Table S1, cited in the above text) which compares visualization targets, strengths, and weaknesses between filoVision and existing filopodia analysis tools.

Lines 867-870
***Table S1. Filopodia Tool Comparison****. Table listing several prominent filopodia analysis tools along with their analysis goals, strengths, and limitations for comparison with filoVision. Potential users should look at this table to determine the best analysis tool for their specific use case.*

**Structure of the manuscript and ci2ng original U-net papers**
We thank the reviewers for their comments on the structure of the manuscript. See rebuttal above for the main changes resulting from these comments. We feel the manuscript has improved because of them.
We also thank the reviewers for pointing out that the original U-net paper wasn't being cited. This error has been corrected.

Lines 128-132:
*The U-net convolutional neural network architecture was chosen because it enables successful model training on very few images compared to other architectures partially due to its training strategy involving data augmentation (Ronneberger et al., 2015). This has played a large role in the U-net architecture being widely adopted by the biological and medical communities for image segmentation applications.*

**---Reviewer-Specific Responses---**

**REVIEWER 1**

Thank you for your feedback! We appreciated your though4ul comments. The suggestions were quite helpful and have led to significant improvements and better ways to represent our data. Many of your comments are addressed above.

**I recommend the authors use the F1 and panop2c quality scores to assess their segmenta2on and not only the Intersec2on over union metric, especially for the filopodia 2p and filopodia shaW classes.**
Thank you for pointing this out. Additional important segmentation scores including F1 and panoptic quality scores have been calculated and added to the manuscript in supplementary Table S3. We included panoptic quality scores, but we also note that filoVision uses semantic segmentation not instance or panoptic segmentation, which to our knowledge is primarily when panoptic quality scores are used.

**I recommend the authors show the correla2on between their manual analyses and their automated analyses, not the overall measurements.**
Correlation plots were a great idea and indeed are a much better way to compare filoVision measurements. This was done for both filoTips and filoSkeleton and the plots added to the manuscript (see examples for new Figs 2C and 3B, below). A random number array with the same sample number was included with each correlation plot as a non-correlated control to further evaluate the robustness of the correlation.

© 2024. Published by The Company of Biologists under the terms of the Creative Commons Attribution License (https://creativecommons.org/licenses/by/4.0/).

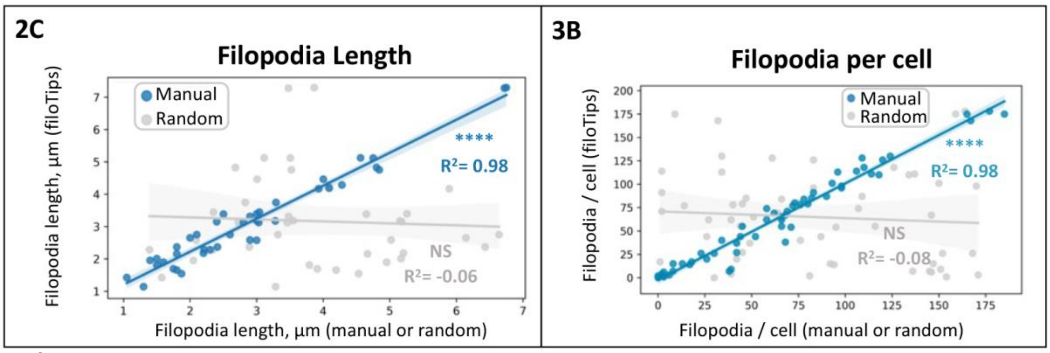


**(le$) Figure 2. filoTips automated measurements compared to manual measurements.** **(B)** Correla(on of filopodia per cell measurements between filoTips and either manual measurements (N:6, n:54, $R^2$: 0.99, P-Val: 1.37e$^{-41}$, blue) or a random number control array (n:54, $R^2$: -0.02, P-Val: 0.88, gray) using two-sided Pearson correla(on coefficient (PCC).

**(right) Figure 3. Tuning filoTips for U2-OS and COS-7 data (B)** Filopodia per cell measurement correla(on (PCC) between filoTips using the U2-OS and COS-7 tuned model and either manual measurements (N:6, n:56, $R^2$: 0.98, P-Val: 2.03e$^{-39}$, blue) or a random number control array (n:56, $R^2$: -0.08, P-Val: 0.56, gray).

**How does filoVision perform when filopodia are broken (fragmented ac2n stain) or crossed? Or when the filopodia density is very high?**
filoSkeleton now tracks the filopodia shaX from the tip to base of filopodia, enabling it to detect broken or disembodied filopodia and ignore them if they are at least 5 pixels away from the cortex.
Disembodied filopodia are not included in the final counts and are marked with a blue filopodia tip in the annotations.

Lines 612- 614:
*This provides filopodia shaa length information and allows filoSkeleton to detect broken or disembodied filopodia and ignore them if they are at least 5 pixels from the cortex (yellow stalk with a blue tip).*

Crossed filopodia are a bit more challenging. It should be noted that these are relatively rare in most cells, but it they are more prominent in cells making an unusual excess of filopodia. Below has now been added to the “Limitations and other considerations” section in the Discussion.

Lines 385-392:
*Occasionally, while tracking a filopodia shaa, it will confuse the correct shaa with one that temporarily overlapped and begin tracking that shaa back to the cortex. In our experience, the error associated with this was usually minor as the relatively rare crossing events were more likely to occur near the cortex and had similar remaining shaa lengths from the crossing event to the cortex. However, in some cases, it will cause incorrect length measurements. The annotations make it so users can see if an event like this occurred, and if so, all other measurements including filopodia number per cell remain valid, but the shaa length of that event is inaccurate.*

filoTips and filoSkeleton both handle high filopodia density quite easily in our experience. In the case of filoTips – the U2-OS cell test dataset had cells with filopodia numbers ranging from 1-168 filopodia/cell. At the highest densities in this example, cells are making 1 filopodia every 3.1 µm (cell perimeter), on average. For filoSkeleton – the test dataset had cells with filopodia numbers ranging from 11-89 /cell. In this case, cells with the highest densities of filopodia are making 1 filopodia every 2.9 µm (cell perimeter), on average (see Fig. 5A, below, for a representative cell with high filopodia density). It’s possible that some cells might have an even higher density than this, but in our opinion the filopodia density observed with the test datasets above are quite high indeed.

© 2024. Published by The Company of Biologists under the terms of the Creative Commons Attribution License (https://creativecommons.org/licenses/by/4.0/).

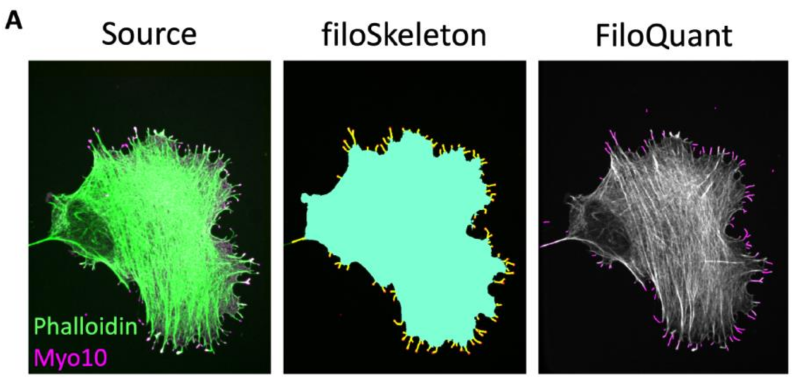


**Figure 5. Comparison of filoSkeleton measurements with FiloQuant analysis. (A)** Representa(ve HeLa cell stained with Alexa 488 phalloidin (green) to visualize the ac(n cytoskeleton and an(-Myo10 an(bodies (red) showing the filoSkeleton analysis annota(on. filoSkeleton annota(on - aqua: body, yellow: filopodia stalks, red: filopodia (ps. FiloQuant annota(on – purple: filopodia stalks.

## REVIEWER 2

Thank you for the colorblind-inclusive reminder and the recommendation to add a section that highlights the biological significance of filoVision (see above).

**The approach of exploi2ng filopodia 2p markers to iden2fy filopodia will be useful, especially as some cytoskeleton and cell membrane probes are known to not reliably mark the far 2p, but has its limita2ons. Because spots of 2p protein are segmented individually then assigned to the nearest cell as filopodia, this approach is vulnerable to errors when 2p proteins are dim, appear as spots in the filopodia shaW, or when cells are close together 2ps may be assigned to the wrong cell. The authors show that this approach gives accurate measurements for filopodia numbers and length, but more complex morphological parameters (e.g. sweeping mo2ons over 2me) would be hard to include as the filopodium shape and base posi2on are not specifically measured. In cells with filopodia 2ps marked by overexpressed 2p proteins such as Myosin X or DdMyo7 overexpression ar2facts are likely.**
[and] **filoVision isn't set up for tracking filopodia over 2me in live imaging datasets**

The reviewer rightly points out potential problems and limitations in using filopodia tips for analysis, including dim tips, shaX spots and artifacts, and close cells. Many of these limitations are associated with using filopodia tip markers instead of an actin stain, in general, yet many labs (including ours) use tip markers routinely for filopodia analysis throughout many publications. Here we now better highlight strengths and weaknesses of using tip markers and actin stains in the introduction (Lines 90-102). In our opinion, analysis of closely packed cells is a very minor issue because typically cells are plated at a very low density regardless of analysis method to avoid cell/filopodia neighbor overlap. If cells are close enough for filoTips to confuse filopodia tip assignment (which can happen), we would recommend using a lower cell density no matter the analysis method to avoid this potential overlap. If tracking is required, the potential user should use a tool developed for that purpose like Filopodyan or U-shape 3D, however it should be noted that we are in the process of implementing a filoTips feature that would allow filopodia tip tracking. It is not ready to be released yet so won't be included here, but we expect to provide this update via the GitHub repo in the near future. Measuring shaX sweeping motions in live cells and filopodia shapes is more complex than the core goal of filoVision. If users require this more sophisticated approach and have cells expressing LifeAct we recommend a tool with a goal suited for this type of analysis like U-shape 3D (Driscoll et al., 2019) and have added Table S1 which highlights goals, strengths, and weaknesses of various filopodia analysis workflows. A new section discussing these issues and filoVision limitations is now provided in the discussion.

see new section in Lines 380-427:
***Limita-ons and other considera-ons***

© 2024. Published by The Company of Biologists under the terms of the Creative Commons Attribution License (https://creativecommons.org/licenses/by/4.0/). 

**Though the authors point out the need for a deep learning-based tool for filopodia segmenta2on, exis2ng deep learning tools (e.g. generalist cell segmenta2on models) that might be applied to this task are not discussed or benchmarked against.**

We have added a comparison between filoSkeleton and FiloQuant. The deep learning tool U-shape3D can analyze 3D filopodia and has been added to Supplementary Table S1 which compares the analysis goals, strengths, and weaknesses of different filopodia tools. It should be noted that the goal of U- shape3D is to analyze 3D actin-labeled cell protrusions in detail. This is quite useful, however does not share the same goal of filoVision – automated, rapid analysis of 2D tip-marked filopodia made by a diverse array of cell types without requiring a cytoskeletal label (filoTips) or having to crop out cells individually. As for cell segmentation models: filoTips – we are unaware of an existing deep learning model that generates segmentation predictions for both cell bodies and filopodia tips. filoSkeleton – we are also unaware of an existing deep learning model that generates segmentation predictions for cell bodies, filopodia shaXs, and filopodia tips. We don't make claims that they don't exist, just that we don't know about them.

**Detailed comments on Results -**

**mNeonGreen instead of mNeon**
Thanks for pointing that out - Fixed throughout manuscript

**(pg 9) Explain how the cortex is defined, since it is not included in the segmenta2on mask.**
This information has been added to the methods.

Lines 504-510:
*The cortex is identified by increasing the thickness of the contour edge (cell edge) outline by 15 pixels and assigning the thin ~6 pixel wide, inner band that overlaps with the existing cell contour as the cortex, or cell body edge (marked as blue and orange in filoTips annotations). Another ~15 pixel gray band is introduced to separate the cortex (blue/orange) and cell body (yellow) for more accurate tip protein signal assignment (cortex or body) during extraction (Fig. 2A bottom).*

**(pg 12) Explain the use of the OpenCV detectContours func2on to define cell bodies as this wasn't explained in the filoTips sec2on.**
An explanation has now been provided in methods.

Lines 495-502
*filoTips utilizes OpenCV (Bradski, 2000) contours to convert model segmentation predictions into individual cell body and filopodia tip objects (Fig. 1). Pixels belonging to the body class in the segmentations are extracted. Contour detection then searches for connecting body pixels and provides the contour, or outline, of the connected pixels. This allows cell body object assignment with a numerical identifier and provides body object coordinate information. If multiple cell contours are detected, the cell body contours are measured iteratively using OpenCV image moments like contourArea and arcLength to extract pixel measurements that are converted to micron measurements.*

**(pg 12) More detail on assignment of 2ps to cells - "if the 2ps are adjacent to a filopodia stalk" - how close?**
More detail has now been provided in the methods.

Lines 600-618
*filoSkeleton utilizes OpenCV contours (Bradski, 2000) to convert model segmentation predictions into cell body, filopodia stalk, and filopodia tip objects (Fig. 4). Like filoTips, filoSkeleton uses contour detection to first detect the edges of all cell bodies in an image and assign them as cell body objects (aqua, Fig. 6A). For each cell body object, related measurements are extracted iteratively as for filoTips (see filoTips default model training and filopodia detection). Aaer all cell bodies have been detected, contour detection is performed on the stalk segmentation to detect filopodia stalks and record their coordinates. Finally, contour detection is used to locate filopodia tip foci. If a tip foci is within 3 pixels of a filopodia stalk, it is assigned as a filopodium. For each detected tip contour, the tip protein signal is extracted and a tip/body signal ratio is*

© 2024. Published by The Company of Biologists under the terms of the Creative Commons Attribution License (https://creativecommons.org/licenses/by/4.0/).

*calculated. Starting at the tip, it finds the direction of the nearest cortex and in that direction, moves 5 pixels at a time along the stalk until it reaches the cortex/filopodia base interface (yellow stalk with a red tip). This provides filopodia shaa length information and allows filoSkeleton to detect broken or disembodied filopodia and ignore them if they are at least 5 pixels from the cortex (yellow stalk with a blue tip). A record is kept of the number of filopodia tips assigned to the different cell body objects and aaer all filopodia have been assigned, the filopodia number per cell metric is calculated. Lastly, annotations (Fig. 6A) are exported along with clear summary tables (see Table S5 for examples).*

**(pg 13) Explain what is meant by "manually enhance the dim filopodia 2ps in imageJ".**
We appreciate that this was confusing and should not be included. It has been removed from the manuscript.

**detailed comments on Figures -**

**Graphical abstract: green and red colour scheme should be made colourblind-friendly.**
Thank you for prompting us to correct this oversight. The color scheme has been adjusted throughout by changing red to magenta where appropriate. See graphical abstract and Fig. 5A below

Graphical abstract

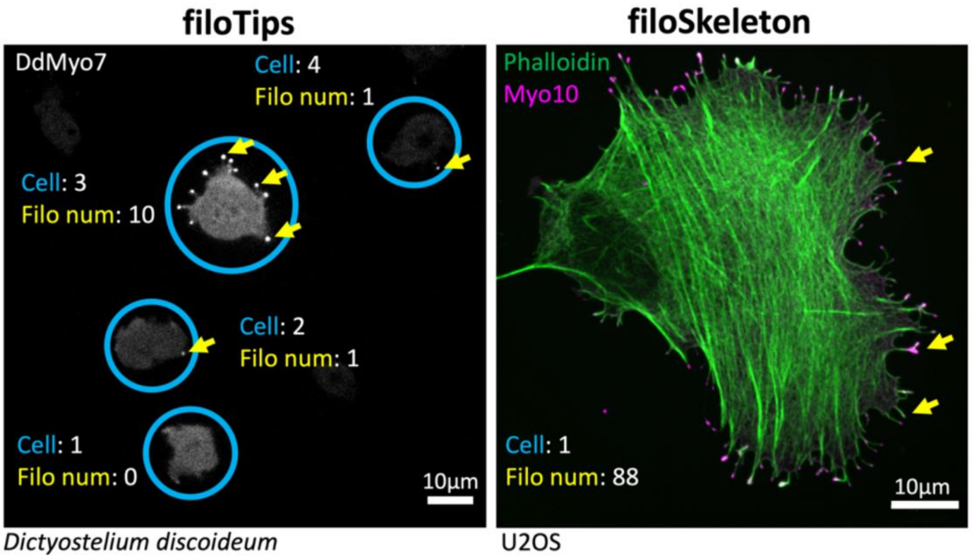


**Fig3: Explain how the annota2ons for cortex and spacing are assigned. Nothing appears to be assigned to the blue regions in Fig 3B.**

The original Fig. 3 has been removed due to reviewer comments about excessive figures and instead we describe the annotation colors in new Fig. 2A. Explanations for defining cortex and spacing are provided above. Thank you for pointing out that the blue region wasn't described in the figure.
DdMyo7 becomes enriched at the cortex, even more so at the leading edge or the front part of the cell during migration. Because of this, we (and potentially others) have an interest in quantifying the most intense region (~50 pixels in length) of the cortex labeled blue in the annotations. The blue region has now been described in the text and figure legends.

Lines 512-515
*Due to our interest in measuring asymmetrical enrichment of DdMyo7 at the cortex, a metric was included which scans for the strongest signal within the cortex, extracts signal from the surrounding ~50 cortex pixels, and labels it the "leading edge" (the blue section of the cortex in filoTips annotations).*

© 2024. Published by The Company of Biologists under the terms of the Creative Commons Attribution License (https://creativecommons.org/licenses/by/4.0/). 

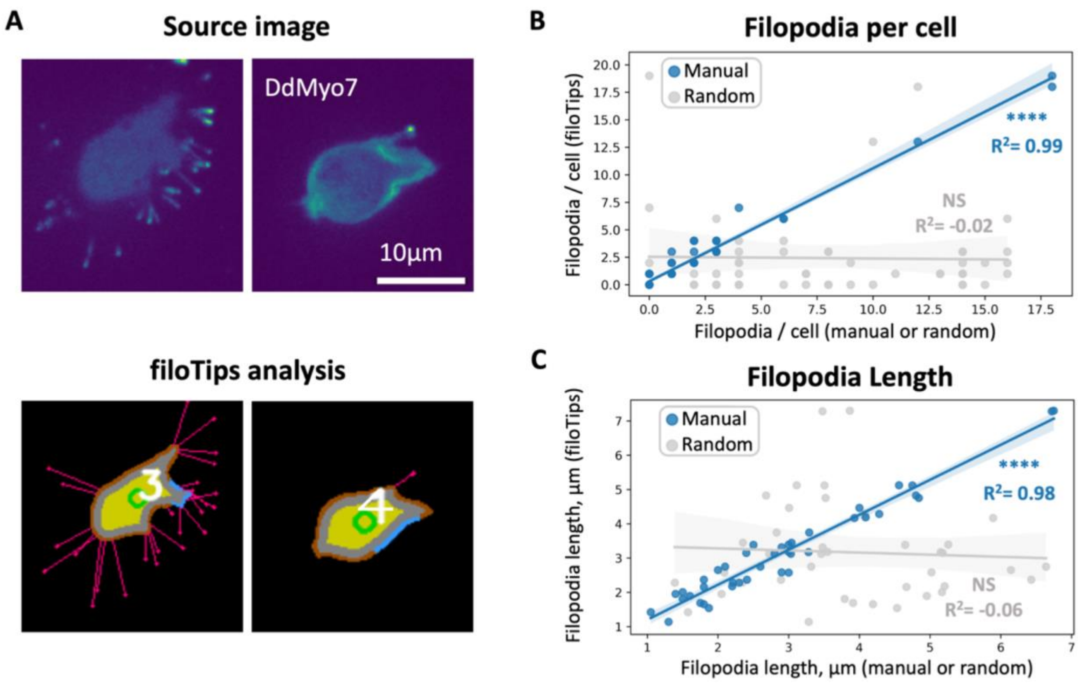


***Figure 2. filoTips automated measurements compared to manual measurements.*** *(A) Representative Ddisc cells making either a high or low number of filopodia and the filoTips analysis annotation for each. Annotation colors: yellow: body, orange: cortex, pink: filopodia, blue: area of highest signal intensity in cortex, gray: separation between cortex and body, green: centroid, and white: cell number in image. (B) Correlation of filopodia per cell measurements between filoTips and either manual measurements (N:6, n:54, R2: 0.99, P-Val: 1.37e-41, blue) or a random number control array (n:54, R2: - 0.02, P-Val: 0.88, gray) using two-sided Pearson correlation coefficient (PCC). (C) Correlation of filopodia length measurements (PCC) between filoTips and either manual measurements for 40 random filopodia from 20 random cells (n:40, R2: 0.98, P-Val: 1.87e-26, blue) or a random number control array (n:40, R2: -0.06, P-Val: 0.73, gray).*

**Fig4: Explain what sta2s2cal tests have been used, e.g. in three-way comparisons, is it pairwise?**

One-way ANOVA with pairwise comparisons were used for three-way comparisons in the original Fig. 4. However, that figure has been replaced with revised Fig. 2 which no longer has a three-way comparison. We appreciate the note that statistical tests need to be more explicit, so we have made each test used more explicit in the figure legends (example legend below).

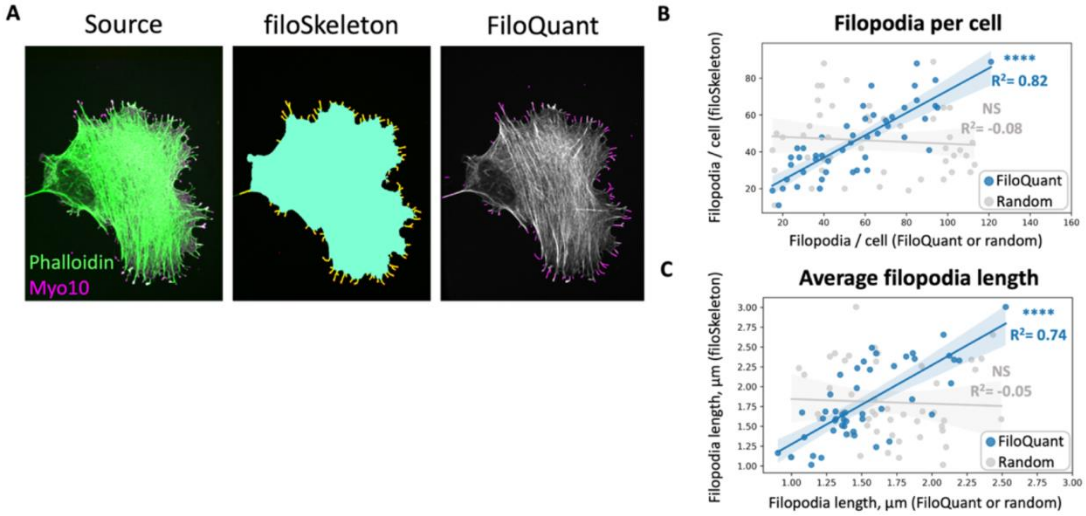


© 2024. Published by The Company of Biologists under the terms of the Creative Commons Attribution License (https://creativecommons.org/licenses/by/4.0/).

**Figure 5. Comparison of filoSkeleton measurements with FiloQuant.** **(A)** Representa(ve HeLa cell labeled with Alexa 488 phalloidin (green) and an(-Myo10 an(bodies (magenta) to visualize the ac(n cytoskeleton and filopodia (ps. filoSkeleton annota(on - aqua: body, yellow: filopodia stalks, red: filopodia (ps. FiloQuant annota(on – purple: filopodia stalks. **(B)** Correla(on of filopodia per cell measurements (PCC) between filoSkeleton and either FiloQuant (N:5, n:47, $R^2$: 0.82, P-Val: 3.07e$^{-12}$, blue) or a random control array (n:47, $R^2$: - 0.08, P-Val: 0.60, gray). **(C)** Correla(on of average filopodia length measurement (PCC) between filoSkeleton and either FiloQuant (N:5, n:47, $R^2$: 0.74, P-Val: 3.0e$^{-09}$, blue) or a random control array (n:47, $R^2$: -0.05, P-Val: 0.76, gray). **(C)** Correla(on of average filopodia length measurement (PCC) between filoSkeleton and either FiloQuant (N:5, n:47, $R^2$: 0.74, P-Val: 3.0e$^{-09}$, blue) or a random control array (n:47, $R^2$: -0.05, P-Val: 0.76, gray).

*Explained in previous figure legend – PCC: Two-sided Pearson correlation coefficient.

## REVIEWER 3

We greatly appreciate your though4ul and detailed comments, which undoubtably helped to significantly improve the manuscript. You raise many important points regarding the potential usefulness of filoVision to the field. The reviewer is right to challenge us on these points, but we believe that filoVision will provide researchers in the field with a user-friendly, reliable, and efficient analysis workflow that is unique because it enables analysis of tip-marked filopodia without requiring an additional cytoskeletal label unless desired. As a lab that uses an amoeboid model to study filopodia, we found that many existing tools don't work well with our cells. Thus, we believe the flexibility filoVision offers through transfer learning accessible via ZerotiostDL4Mic will further extend its utility and appeal for those working with more diverse cell types as well.

**There is not enough informa2on provided on the nature of the data used for the training and tes2ng of the deep learning models, nor is much informa2on provided about how the models were trained, why a par2cular model architecture was used, etc. There's a general over-reliance on the ZeroCostDL4Mic plaiorm to provide such details.**

More information about the training and test datasets is now provided in new Supplementary Table S2 and in the text. It details the train/validation dataset and test dataset for each model separated by individual sheets. We now refer to Table S2 when discussing training and test data - see examples below.

Lines 458-463 (for Ddisc):
*The training dataset consisted of 385 images of live vegetative Ddisc cells. Wild type and filopodia mutant amoeba (myo7 or vasp null; Tuxworth et al., 2001 and Han et al., 2002) expressing DdMyo7 tagged with GFP or mCherry or mNeonGreen were included in the training dataset (Table S2; Arthur et al., 2019; Arthur et al., 2021; Petersen et al., 2016; and unpublished).*

Lines 587-589 (for mammalian cells):
*The model for segmenting filopodia tips was trained on a dataset of 121 images of U2-OS cells ectopically expressing and immunostained for Myo10 and/or FMNL3 (Table S2).*

We acknowledge an overreliance on ZeroCostDL4Mic to describe model training parameters. More detail has now been added which hopefully addresses this problem. See example below.

Lines 478-483
*Data augmentations shia, zoom, shearing, flip, and rotation were used to artificially increase the size of the training data (Ronneberger et al., 2015). The following training parameters were used: epoch number: 300, image patches: 279, patch size: (512,512), batch_size: 4, number of steps: 0, pooling steps: 2, percent validation: 20, initial learning rate: 0.0003, patch dimensions: 512x512, min fraction: 2%, loss function: acategorical_crossentrop.*

© 2024. Published by The Company of Biologists under the terms of the Creative Commons Attribution License (https://creativecommons.org/licenses/by/4.0/).

We have added text to describe some of the advantages of using the U-net architecture and why it was chosen.

Lines 128-132
*The U-net convolutional neural network architecture was chosen because it enables successful model training on very few images compared to other architectures partially due to its training strategy involving data augmentation (Ronneberger et al., 2015). This has played a large role in the U-net architecture being widely adopted by the biological and medical communities for image segmentation applications.*

**Details about how ground truths are generated is confusing at 2mes.**
We agree and have now provided more detail about generating ground truths. See two examples below.

Lines 465-477
*Generating ground truths for training can be a tedious task, thus tools like Amazon SageMaker, V7 labs, Labelbox, and Ilastik can be used to expedite the image labeling process for deep learning applications. Ilastik is free and is designed for the biomedical community (Berg et al., 2019), thus its pixel classification mode was chosen to generate ground truths for the default filoTips model. Ilastik was provided with batch sizes of 20 images, all features were used and set to the highest seungs, and 3 labels were selected (0- background, 1- cell body, and 2- filopodia tips). Using each label's "paintbrush", Ilastik is shown the correct assignment by the user and begins to auto-assign pixels. This process was repeated until all pixels in the 20-image batch have been assigned the correct label. The ground truths were exported as simple segmentation .tiff files and a new project was started for the next batch of images until ground truths are generated for all data.*

Lines 575-581
*Ground truths for cell bodies and filopodia stalks were generated using the "filoSkeleton Body_Stalk Ground Truth Generator" ImageJ macro (GitHub filoVision repository) which masks the cell, shaves off the filopodia stalks through 5 rounds of erosion and dilution to isolate the cell body pixels like workflows such as ADAPT (Barry et al., 2015), and then generates a ground truth segmentation consisting of 3 labels (0- background, 1- cell body, 2- filopodia stalks; Fig. S5A).*

**I also think the authors need to further test their plaiorm on a wider variety of datasets to demonstrate the robustness of their plaiorm. At present, the models are only tested on data par22oned from training data sets. For example, why was the filoTips model not tested on the fixed U2-OS cells? The training datasets are very small (only 460 images for filoTips, for example), which may mean the models do not generalise very well to new, previously unseen data.**
The reviewer raised an important point and we considered ways to test how well the model would work for other's data and because we did not have easy access to additional data, we decided to see how well transfer learning would work.
The pla4orm was tested on model unseen test data similar to the training dataset. Data used for training and validation were not used for testing the pla4orm (this has been made more explicit in the document, see above response).
A primary reason for choosing the U-net architecture and data augmentation was that it allows for successful model training on a small sample size. It would be quite challenging to acquire thousands of diverse images for training, thus we chose to now highlight how easy transfer learning is with ZeroCostDL4Mic.
We briefly test the default filoTips model trained on amoeboid cells on U2-OS and COS-7 cells, which are quite different from each other. This was mostly done to compare model performance pre and post- transfer learning (see Table S6). We note that filoTips filopodia counts using the default model (prior to finetuning) strongly correlate with manual counts in ImageJ ($R^2$ = 0.94). Already, this is quite a strong correlation and was enhanced further via finetuning ($R^2$ = 0.98; Table S6). In our experience, the default model did well at predicting filopodia tips, however we noticed it would oXen underestimate the relatively dim U2OS or Cos7 cell edges which would impact the measurement of filopodia lengths, which was ultimately fixed by tuning (see Lines 202-212).

© 2024. Published by The Company of Biologists under the terms of the Creative Commons Attribution License (https://creativecommons.org/licenses/by/4.0/).

To quantify the performance, we compared F1-scores of the models pre and post finetuning using body segmentation predictions from both models and ground truths. The F1-scores improved from
0.89 with default model to 0.96 with the finetuned model. Ultimately, we believe this demonstrates good generalization from Ddisc to mammalian cells, with performance improved even further with transfer learning. Therefore, users should first try our models on their data and they can then , but we tune these models to their own data for the most accurate measurements possible.

The filoTips model wasn't tested on the fixed U2-OS cells because the Myo10 immunostaining process was still being worked out, thus while the tip signal is strong, the Myo10 signal in the cell body is quite weak, making it unsuitable for filoTips but the analysis did work with filoSkeleton.

**filoVision isn't compared to other published tools.**
This was an oversight and we now directly compare filoSkeleton measurements to FiloQuant (single image analysis mode) measurements to demonstrate filoSkeleton as a reliable filopodia analysis tool (See lines starting at 340 and Fig. 5 above). FiloQuant was chosen because it is an analysis tool that has been used by a few labs now and, like filoSkeleton, it too uses an actin label in its workflow. Unlike filoSkeleton, filoTips wasn't compared to another published tool because we aren't aware of an existing tool that measures filopodia using tip markers without requiring a second cytoskeletal label. We also now briefly discuss FiloQuant and Filopodyan in the introduction highlighting that different tools have different visualization targets and goals. We also now refer potential users to Table S1 which compares existing filopodia analysis tools, demonstrating their goals, strengths, and weakness for potential users to examine and determine the tool that best suits their analysis goals.

**8 figures seems excessive.**
We appreciate this comment and have made an effort to include fewer figures. The figures have been condensed down to 5, more concise figures.

**Absence of line numbers makes it difficult to reference specific points.**
Our apologies for the omission - line numbers have been added to the manuscript.

**INTRODUCTION:**

**Should end with a paragraph summarizing the key findings of the paper. What is filoVision? How does it differ from other soWware? What results were achieved with it? Etc.**
We agree with the big picture statement that the transition into the results needed improvement. We have edited the manuscript to end the introduction with a brief summary of available tools and highlight the lack of an automated workflow for analyzing 2D filopodia made by diverse cell types using a tip marker without requiring a cytoskeletal label or cropping out cells individually. This then leads naturally into the brief intro to filoVision at the beginning of the Results.
Note that we choose not to use the popular "second abstract" style for the introduction ending, but instead make a statement of the purpose of the work that sets up the basis for the experiments and results that follow.

Lines 104-120:
*Many tools have been developed to measure filopodia, demonstrating that there is high demand for workflows that quantify filopodia production. These tools are typically targeted towards a certain cell type and/or filopodia visualization method, and each have their own strengths and limitations (Table S1; Barry et al., 2015; Driscoll et al., 2019; Jacquemet et al., 2017; Mousavi et al., 2020; Nilufar et al., 2013; Tsygankov et al., 2014; Urbančič et al., 2017). For example, FiloQuant and Filopodyan each have different visualization targets. (Jacquemet et al., 2017; Urbančič et al., 2017) - FiloQuant uses an actin label to identify and measure filopodia stalks protruding from a cell body, whereas Filopodyan uses a membrane marker with the option of including a tip marker to measure filopodia and their dynamics over time. To the best of our knowledge, a tool specifically designed to measure filopodia using a tip marker alone or in combination with an actin label doesn't exist, yet many use tip markers in their filopodia analysis*

© 2024. Published by The Company of Biologists under the terms of the Creative Commons Attribution License (https://creativecommons.org/licenses/by/4.0/).

*workflow (Petersen et al., 2016; Jacquemet et al., 2019b). Furthermore, it can be difficult to find tools adaptable for diverse cell types like amoeba, likely due to most tools being designed for working with mammalian cells. Thus, filoVision was developed to address the lack of an automated workflow for measuring tip-marked filopodia in diverse cell types like amoeba.*

**RESULTS:**

**First paragraph is largely introductory and could be moved to end of introduc2on.**
We agree with the reviewer that the first results paragraph needed improvement. We have edited this paragraph to provide more of an overview of filoVision while still being able to introduce the pla4orm briefly. We chose to end the introduction by discussing why filoVision was initially developed - to address the lack of an automated workflow for measuring tip-marked filopodia in diverse cell types like amoeba (see lines starting at Line 123).

****Training a filoTips model:**

**It is stated that "Wild type and filopodia mutant amoeba (myo7 or vasp null) expressing wild type or non-func2onal mutant DdMyo7 tagged with a variety of fluorophores, including GFP, mCherry, and mNeon were used" - how many of each were used? A much more detailed breakdown of what data was used for training, valida2ng and tes2ng the model should be provided**
Thank you for pointing this out. First, we mistakenly stated that some cells expressed a non-functional DdMyo7, this phrase has been removed (line 459). Information about the cell lines (genetic background and DdMyo7-fluorescent protein fusion) is now provided in a new supplementary table, Table S2 where a detailed listing of the training/validation data and test data for each model is provided and separated by sheets.

Lines 459-463
*Wild type and filopodia mutant amoeba (myo7 or vasp null; Tuxworth et al., 2001 and Han et al., 2002) expressing DdMyo7 tagged with GFP or mCherry or mNeonGreen were included in the training dataset (Table S2; Arthur et al., 2019; Arthur et al., 2021; Petersen et al., 2016; and unpublished).*

**It is men2oned that ilas2k was used to generate ground truth data, but only very vague details are provided (along with some concerning statements about manual correc2on being required - what does this mean exactly?) and there is no men2on of ilas2k in the materials and methods sec2on. The genera2on of ground truth segmenta2ons should be detailed in a dedicated sec2on under materials and methods.**
We have moved this information to the materials and methods section and agree it's more appropriate. We have briefly provided more details regarding Ilastik.

Lines 465-477
*Generating ground truths for training can be a tedious task, thus tools like Amazon SageMaker, V7 labs, Labelbox, and Ilastik can be used to expedite the image labeling process for deep learning applications. Ilastik is free and is designed for the biomedical community (Berg et al., 2019), thus its pixel classification mode was chosen to generate ground truths for the default filoTips model. Ilastik was provided with batch sizes of 20 images, all features were used and set to the highest seungs, and 3 labels were selected (0- background, 1- cell body, and 2- filopodia tips). Using each label's "paintbrush", Ilastik is shown the correct assignment by the user and begins to auto-assign pixels. This process was repeated until all pixels in the 20-image batch have been assigned the correct label. The ground truths were exported as simple segmentation .tiff files and a new project was started for the next batch of images until ground truths are generated for all data.*

The "manual corrections" statement is indeed very confusing. The intent behind this statement was to indicate that Ilastik learns from manual annotations to assign pixel class and improves overtime as you "correct" its predictions with the Ilastik paintbrush tool. To avoid confusion, this statement was removed entirely and replaced with above.

© 2024. Published by The Company of Biologists under the terms of the Creative Commons Attribution License (https://creativecommons.org/licenses/by/4.0/).

**Default data augmenta2ons and default training parameters… what does this mean?**
We acknowledge this isn't very informative. These statements were referring to default parameters described in the ZeroCostDL4Mic 2D U-net notebook. We have now added these details to the methods section of the manuscript instead of relying completely on ZeroCostDL4Mic to describe them.

Lines 478-483
*Data augmentations shia, zoom, shearing, flip, and rotation were used to artificially increase the size of the training data (Ronneberger et al., 2015). The following training parameters were used: epoch number: 300, image patches: 279, patch size: (512,512), batch_size: 4, number of steps: 0, pooling steps: 2, percent validation: 20, initial learning rate: 0.0003, patch dimensions: 512x512, min fraction: 2%, loss function: acategorical_crossentrop.*

**"Intersec2on over Union" explana2on in the results, it should be moved to methods**
We again thank the reviewer for feedback regarding the structure of the manuscript. The IoU explanation has been reduced and moved to methods.

Lines 483-493
*Segmentation predictions for 76 out-of-sample test images were compared to ground truths and scored using metrics like intersection-over-union (IoU), F1, and panoptic scores (Table S3, Fig. S4B). IoU measures the overlap between predicted and ground truth segmentation masks using the ratio of their intersection to their union and is provided by the ZeroCostDL4Mic notebook aaer model evaluation. F1- score combines precision and recall into a single value to assess the accuracy of the segmentation. filoVision uses semantic not instance segmentation (U-net, Ronneberger et al., 2015), but panoptic quality, which is typically used to evaluate predictions that involve the combination of semantic and instance segmentation for categorizing objects, was also calculated as part of the comprehensive evaluation process.*

**Later in the same paragraph, it is stated that "The average IoU for the default filoTips model is 0.76 ± 0.10" - when tested on what? Training data? Unseen test data?**
Unseen test data or out-of-sample data - this was clarified further in the revised manuscript. For any evaluation, the test data is of model unseen data and we have now tried to state that explicitly in the manuscript.

Lines 483-485
*Segmentation predictions for 76 out-of-sample test images were compared to ground truths and scored using metrics like intersection-over-union (IoU), F1, and panoptic scores (TableS 3, Fig. S4B).*

Lines 151-154
*To establish filoTips as a reliable analysis tool, its output was compared to manual analyses in ImageJ (Schneider et al., 2012) using 54 out-of-sample (independent test dataset not yet seen by the model) Ddisc cells expressing mNeonGreen-DdMyo7 (Fig. 2A, Methods, Table 2).*

**The paragraph finishes with the statement "The final evalua2on of filoTips is filopodia 2p detec2on accuracy ... however this is highly dependent on model performance" - I don't understand this statement? The performance of filoTips is highly dependent on the performance of filoTips?!?**
We see how this could be a confusing statement. The intention was to state that scoring filopodia measurements are critical in addition to scoring model segmentation predictions (IoU, F1-scores, etc.). See text on lines 151-154 (above) and Figures 2, 3, and 5 that show measurements for scoring filopodia.

**** Detec2ng and analyzing filopodia with filoTips**

**The sec2on starts with "A trained and deployed filoTips model should now be ready to generate accurate predic2on segmenta2ons of cell bodies and filopodia 2ps in user's data". This is an odd statement with which to begin a results sec2on. The authors should be trying to**

© 2024. Published by The Company of Biologists under the terms of the Creative Commons Attribution License (https://creativecommons.org/licenses/by/4.0/). 

**persuade us that their model works when trained on their data, not sugges2ng it might work on different data!**

This was another poorly phrased statement and was not our intended suggestion. As the reviewer pointed out, the original manuscript sometimes read like a user manual and our initial intent was simply to transition the user from training a model to using it. This statement has been removed from the manuscript.

**The following statement on spa2al separa2on of cells should come later in a general discussion of the limita2ons of the pla▯orm, although I note that this criteria is not tested at any point - how separated do cells have to be in order for filoTips to work?**

We now have included a new section at the end of the Discussion "Limitations and other considerations" section in the discussion (lines 380-427) that includes a discussion of this issue.

When imaging filopodia, sparse cell density is required, regardless of the analysis method, it is necessary to avoid densely plated samples to prevent neighbor body/filopodia overlaps, which will result in unseen filopodia and an underrepresentation of filopodia number. It is possible for filoTips to assign a filopodium to the wrong cell at normal cell densities appropriate for typical filopodia analysis, but this is rare, again because of the inherent need for spatial separation when imaging filopodia. For example, we commonly observe an average filopodia length of ~2.8 µm for Ddisc cells, therefore we try to plate cells at a density that would provide a separation > 10 µm between cells to avoid overlap regardless of analysis method. The exact density will, of course, depend on the cell type.

We are happy to quantify this if necessary, but we decided to prioritize the many other though4ul comments provided by the reviewers.

**How are parameters like area, aspect ra2o, pixel intensity extracted? How are filopodia 2ps assigned to the nearest cell body? How are metrics like filopodia per cell, length, and 2p intensity extracted?**

These details have been added to the methods section.

Lines 495-537:

*filoTips utilizes OpenCV (Bradski, 2000) contours to convert model segmentation predictions into individual cell body and filopodia tip objects (Fig. 1). Pixels belonging to the body class in the segmentations are extracted. Contour detection then searches for connecting body pixels and provides the contour, or outline, of the connected pixels. This allows cell body object assignment with a numerical identifier and provides body object coordinate information. If multiple cell contours are detected, the cell body contours are measured iteratively using OpenCV image moments like contourArea and arcLength to extract pixel measurements that are converted to micron measurements. Image moments within the OpenCV library like those above enable easy calculations of metrics like area, perimeter, aspect ratio, centroid, and circularity. The cortex is identified by increasing the thickness of the contour edge (cell edge) outline by 15 pixels and assigning the thin ~6 pixel wide, inner band that overlaps with the existing cell contour as the cortex, or cell body edge (marked as blue and orange in filoTips annotations). Another ~15 pixel gray band is introduced to separate the cortex (blue/orange) and cell body (yellow) for more accurate tip protein signal assignment (cortex or body) during extraction (Fig. 2A bottom). Tip protein signal in the body and cortex is then extracted from the image via pixel assignment (body or cortex object) and recorded coordinates. Due to our interest in measuring asymmetrical enrichment of DdMyo7 at the cortex, a metric was included which scans for the strongest signal within the cortex, extracts signal from the surrounding ~50 cortex pixels, and labels it the "leading edge" (the blue section of the cortex in filoTips annotations). Tip marker signal ratios (cortex/body, tip/body, etc.) for each section are calculated and included in the final summary table. Aaer all cell bodies have been detected, contour detection is again performed to detect filopodia tips. During cell body analysis, cell outline coordinates are saved and referred to when assigning filopodia tips to cells. Iteratively for each detected tip contour, the tip protein signal is extracted from the contour via recorded pixel coordinates and a tip/body signal ratio is calculated. The Euclidean distance from the tip to all outline coordinates are calculated and the tip is assigned to the closest cell contour. If a cell outline isn't within 10µm of the tip, it is considered an artifact and not recorded, otherwise this linear distance is recorded as the filopodium's length (pink, Fig. 2A bottom). Again, for many cell types including amoeba this is quite effective for geung accurate lengths. However, if a potential user requires shaa length of long, curled filopodia, filoSkeleton or FiloQuant would be more appropriate. All filopodia analysis*

© 2024. Published by The Company of Biologists under the terms of the Creative Commons Attribution License (https://creativecommons.org/licenses/by/4.0/).

*methods require separation of cell bodies to avoid body and filopodia overlaps and thus loss of filopodia signal. Because of this spatial separation, it is effective to use Euclidean distance to assign filopodia tips to the nearest cell cortex. Sometimes this results in a filopodia being assigned to the wrong cell if cells are nearly touching, however evaluations of filoTips suggest this isn't common and doesn't have a strong impact on the analysis. A record is kept of the number of filopodia tips assigned to the different cell body objects and aaer all filopodia have been detected the filopodia number per cell metric is calculated for each cell. Lastly, the annotations (Fig. 2A) are exported along with summary tables (see Table S4 examples) for cell body and filopodia information extraction.*

**** Evalua2ng filoTips' performance**

**This sec2on begins with a reference to manual measurements made in ImageJ - it's difficult to follow exactly how manual segmenta2ons and manual measurements were made for the purposes of (a) training the model and (b) evalua2ng the model. This should all be detailed in a dedicated sec2on under materials and methods.**

Thank you for pointing this out. Ground truth generation is discussed above. Manual ImageJ measurements for comparing to filoTips measurements is described in materials and methods.

Lines 621-628

*filoTips was compared to manual measurements in ImageJ using base tools like line, oval, and polygon (Schneider et al., 2012). Cell bodies were outlined manually in ImageJ using the polygon tool and metrics like cell area and aspect ratio were calculated using the measurements tool. Fluorescent protein intensities were measured by outlining the body, cortex, and filopodia tips using the polygon and oval tools to obtain the mean fluorescent intensities of each. filoTips: filopodia number per cell was counted manually and filopodia lengths were measured using the line tool along the filopodia shaa in ImageJ.*

**"To be considered a reliable quan2ta2on tool, filoTips measurements should be as similar to manual measurements as possible." - I'm not sure about this statement.**

We agree that this is not quite right, the sentence has been removed from the text.

**"Manual correction of the robust stalks was not performed prior to evalua2on, but the false posi2ve rate can be reduced by erasing them prior to filoTips analysis as needed" - isn't the whole point of automated image analysis that manual interven2on is not required?!? Besides, advising people to selec2vely manipulate their images prior to analysis seems likely to be bad for reproducibility.**

We agree that this was an unfortunate description of how to handle false positives. This statement has been removed.

**"It detected 4.1 ± 4.5 filopodia per cell, which is similar to a manual count of 3.6 ± 4.4" - what do the errors here represent? They seem preGy big?**

The errors represented standard deviation, but this panel was replaced with correlation plots per reviewer 1 recommendations, which indeed is an improvement. We oXen see a large variation in Ddisc filopodia number per cell, however it is notable that the observed variation above is higher than normal and we stated the values weren't typical in the original document. Filopodia number is impacted by many variables including buffers, time in starvation media, and even slight variations in room temperature, to name a few. Because of this sensitivity, we perform experiments side by side to ensure consistency with external factors like room temperature. The even larger than normal deviation was likely due to taking random sample images from different datasets across many dates and differing external factors. When using experimental data we don't expect to have a standard deviation this large.

**"SEVEN's significantly more conserva2ve es2mate results from its parameters being op2mized for GFP-DdMyo7 signal and employing a low threshold cut-off (Petersen et al., 2016), making it unable to generalize to other, brighter fluorescent proteins unless specifically tuned each 2me" - but filoTips has been specifically tuned (trained) to this data, so it's not really a fair comparison? How well does filoTips generalise to other data?**

Mentions of SEVEN have been removed from the manuscript to streamline the document. "How

© 2024. Published by The Company of Biologists under the terms of the Creative Commons Attribution License (https://creativecommons.org/licenses/by/4.0/).

well does filoTips generalize to other data?” – please see next response.

**"It shows that when trained on the user’s data, filoTips is a reliable tool..." - what if it is not trained on the users data? How reliable is it then? Does the model need to be completely retrained in order for it to be in any way useful?**

Thanks for pointing out the confusing statement here. The original manuscript didn’t include transfer learning but encouraged by your comments and those of the other reviewers, we decided to demonstrate how one could use transfer learning to tune our models to their data. We took steps to see how effective our default filoTips model, trained on amoeboid cells, was at predicting filopodia made by U2-OS and COS-7 cells which are quite different from amoeboid cells. Much to our surprise, the amoeboid model was found to do an excellent job predicting U2-OS and COS-7 filopodia tips, needing minor finetuning to better detect the relatively dim U2-OS and COS-7 cell edge (Fig. 3A below is an example). Model tuning (including ground truth generation and training via ZeroCostDL4Mic) was accomplished by a lab member with no prior deep learning knowledge and minimal guidance in less than 48 hours, demonstrating that the transfer learning barrier is minimal (Fig. 3, below). We believe this initial time investment is a good tradeoff for future filopodia analysis tuned specifically for the user’s data, however our models are publicly available, and users are free to use them on their data as well. The transfer learning process is now described in the revised manuscript.

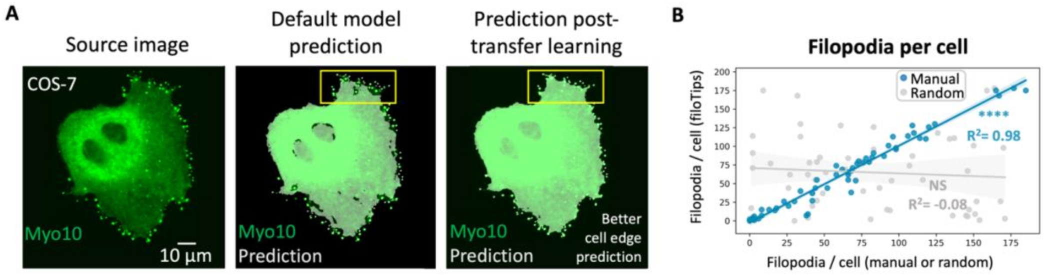


**Figure 3. Tuning filoTips for U2-OS and COS-7 data**
**(A)** Segmenta(on predic(ons before and a^er transfer learning. Le^: Source image of a representa(ve COS7 cell ectopically expressing eGFP-Myo10. Middle: Predic(on made by the default filoTips model. Right: Predic(on made a^er performing transfer learning on U2-OS and COS-7 data. Yellow rectangle highlights an example of improved cell edge detec(on a^er transfer learning. **(B)** Filopodia per cell measurement correla(on (PCC) between filoTips using the U2-OS and COS- 7 tuned model and either manual measurements (N:6, n:56, $R^2$: 0.98, P-Val: $2.03e^{-39}$, blue) or a random number control array (n:56, $R^2$: -0.08, P-Val: 0.56, gray).

**** Training a filoSkeleton model**

**"A well-trained model serves as the founda2on for accurate filopodia detec2on, the true performance metric." - what is a "true performance metric"?!?**

Thank you for pointing out the ambiguity here, this phrase does not accurately convey the intended meaning. We intended to simply say that scoring filopodia detection accuracy is important for a filopodia analysis tool.

Example: Lines 151-154
*To establish filoTips as a reliable analysis tool, its output was compared to manual analyses in ImageJ (Schneider et al., 2012) using 54 out-of-sample (independent test dataset not yet seen by the model) Ddisc cells expressing mNeonGreen-DdMyo7 (Fig. 2A, Methods, Table S2).*

**"The second model takes an image of filopodia 2p foci and segments the pixels into two classes: 0- background and 1-filopodia 2ps" - I'm not sure I understand how this differs from the filoTips model?**

The second filoSkeleton model has two classes: background and tips. filoTips has three: background, cell body, and filopodia tips.
filoTips model description. Lines xxx.
filoSkeleton model description. Lines xxx.

© 2024. Published by The Company of Biologists under the terms of the Creative Commons Attribution License (https://creativecommons.org/licenses/by/4.0/).

**"...default filoSkeleton models are provided, but training a custom model is recommended" - I don't understand why the authors keep making these kinds of statements. Why describe the process of genera2ng the default models if it is recommended that they not be used?**

The reviewer is correct to point out this confusing statement (and others). This sentence has been removed and text detailing the process of generating the default models has been reduced and moved to materials and methods. We further emphasize that the models we provide have worked well for different cell types and that users can employ transfer learning on their own data if finetuning is desired.

See lines 182-189

*A primary objective of filoTips is to prioritize flexibility so that users can fine tune the tool to their own unique datasets and cell types. filoTips models are publicly available (see GitHub repository), in fact filoTips will ask the user if they want to use the default model, and if so, users will automatically have instant access to it without additional steps. They should feel free to test a small representative dataset and use the default model if they are happy with the results. However, we recommend users tune our models to their own data via transfer learning for the most accurate measurements possible.*

**"In some cases cells were treated with siRNA for Myo10 to reduce or eliminate filopodia." - the details of this procedure should be provided in materials and methods.**

Apologies for the omission - the appropriate details have been added.

Lines 681-693

*Cells either underwent siRNA silencing followed by DNA transfection with a mCherry-Myo10 expression plasmid, or were directly transfected with a mCherry-Myo10 expression plasmid. The following siRNAs were used for MYO10 Human siRNA Oligo Duplex (OriGene; Locus ID 4651; ref: CAT#: SR303060 Origene): siRNA_A: 5'-GGTCAGGTATTCACTTACAAGCAGA-3'. siRNA_C: 5'-GGAAAAATACGCTCTCTTCACTTAC-3'). For U2-OS or HeLa cells that underwent silencing, cells were seeded and transfected with siRNAs (10nM) using lullaby reagent (OzBioscience) via a reverse transfection protocol on day 1. This was followed by transfection with a mCherry-Myo10 plasmid using the FuGENE® HD Transfection Reagent (Promega) on day 3, then fixation and image analysis on day 4. For cells that did not undergo silencing, U2-OS or HeLa cells were seeded on day 1, transfected with a mCherry-Myo10 plasmid using the FuGENE® HD Transfection Reagent (Promega) on day 2, then fixed and imaged on day 3 or 4.*

**86 U2-OS cells were used to train the model for segmen2ng cell bodies, but 121 images of were used for training filopodial 2p detec2on? Shouldn't the same mul2-channel images be used to train both models?**

Thanks for pointing out the confusing description. We failed to clarify that some of the cells were immunostained for both FMNL3 and Myo10 tip-marked filopodia. In other words, some images contained 1 actin channel, 1 Myo10 tip-marked channel, and 1 FMNL3 tip-marked channel. Also, some data is of just Myo10-marked filopodia without a phalloidin channel. So, for each cell stained with Myo10 and FMNL3 images from the two channels were included separately in the analysis for the filopodia tip training dataset. Thus, the number of images used in the training set aren't equal to the number of cells. See new Supplementary Table S2 for a more detailed description of train/test data.

Line 587-589

*The model for segmenting filopodia tips was trained on a dataset of 121 images of U2-OS cells ectopically expressing and immunostained for Myo10 or FMNL3 (Table S2).*

**"95%. The average IoU for the model predic2ng cell bodies and filopodia stalks was 0.81 ± 0.09 (Fig. 5C), and the average IoU for the model predic2ng filopodia 2ps was 0.84 ± 0.02 (Fig. 5D), mee2ng the set performance standards." - what data was tested here?**

All test data is out-of-sample. This has been made more explicit throughout the text.

see lines 151-154, for example

© 2024. Published by The Company of Biologists under the terms of the Creative Commons Attribution License (https://creativecommons.org/licenses/by/4.0/).

*To establish filoTips as a reliable analysis tool, its output was compared to manual analyses in ImageJ (Schneider et al., 2012) using 54 out-of-sample (independent test dataset not yet seen by the model) Ddisc cells expressing mNeonGreen-DdMyo7 (Fig. 2A, Methods, Table S2)*

**** Evalua2ng filoSkeleton's performance**

**"The accuracy of filoSkeleton in measuring filopodia was evaluated by comparing its quan2ta2on metrics to manual measurements made using imageJ on a total of 17 images" - that is a very small number of images.**

Yes, we acknowledge that 17 images isn't ideal. We have since acquired more testing data and it has been expanded to a total of 47 images.

**The model that's been trained on more informa2on (ac2n staining and filopodial 2p marker) actually performs worse than the first model (filopodial 2p marker)? Why? And if this is the case, should people not just use filoTips?**

filoTips relies solely on a single tip marker signal. It's relative simplicity and the strong signal to noise ratio seen for this single marker seems to result in more accurate filopodia number measurments (note: filoSkeleton still strongly correlates with FiloQuant measurements suggesting its performance is on par with a lead analysis tool). If users have a robust tip signal, then filoTips is a good option.

However, sometimes users want or need to use an actin stain to confirm the identity of filopodia or shaX lengths. In this case, filoSkeleton can offer enhanced accuracy in shaX length determination because it tracks along the filopodia shaXs.

MATERIALS AND METHODS

**** Hardware and soWware requirements**

**"Although not necessary, it is recommended to use Ilas2k and ImageJ for manual annota2on and image manipula2on tasks" - if it's not necessary, then why is it men2oned in materials and methods?!? And what kind of manual annota2on and image manipula2on is being referred to here?**

We agree and this has been removed from manuscript.

**** Running filoVision**

**This doesn't belong in materials and methods - it's a user guide and should be on the github wiki**

We agree, this has been removed from manuscript and posted in the GitHub repository readme.

**** Default filoTips model data acquisi2on**

**"Various fluorophores" are men2oned - this is far too vague. A detailed list should be provided.**

The fluorescent fusion proteins, Alexa phalloidin and labeled secondary antibodies are beFer described and are all listed in new Supplementary Table S2.

**** Default filoSkeleton model data acquisi2on**

**"Cells were imaged on a spinning disk microscope (SP3) with 60x and 100x objec2ves" - more details of the acquisi2on system are required here.**

The details are now provided in Methods.

Line 649-654 (Ddisc)

*Cells were then imaged with 63x or 100x Plan Apo oil-immersion objectives (NA 1.4) on a Marianas Spinning Disk Confocal imaging system based on a Zeiss Axiovert microscope equipped with a Yokogawa CSU-X1, a Photometrics Evolve 512 electron multiplying (EM) CCD camera, a Photometrics HQ2 CCD camera, an ASI MS-2000 stage controller, and laser lines at 488 and 561nm in a SlideBook 6.0 soaware environment (Intelligent Imaging Innovations).*

© 2024. Published by The Company of Biologists under the terms of the Creative Commons Attribution License (https://creativecommons.org/licenses/by/4.0/).

Line 667-673 (COS7/U2OS)
*Live cell images were acquired on a Nikon Ti2-E microscope equipped with a Crest Optics X-Light V3 spinning disk, 60 x Plan Apo oil-immersion objective (NA 1.4), and captured with a Hamamatsu ORCA- Fusion BT CMOS camera. Samples were illuminated with [800 mW] lasers (488 or 561 nm) with XX filters for 488 or 561-nm excitation (final 0.1099 µm pixel size). 5 Z-stacks of 0.3µm steps were taken with 50-100 ms exposure and 30-60% laser power, and max projections generated for analysis.*

and Lines 695-704
*The actin cytoskeleton was visualized by staining with either Alexa 488-phalloidin or rhodamine phalloidin (Cytoskeleton PHDG1-A; PHDR1 - 1:200 dilution). Images were acquired on a spinning disk system (Gataca Systems) based on an inverted microscope (Ti-E; Nikon) equipped with a sCMOS camera (Prime 95B; Photometrics), a confocal spinning head (X1; Yokogawa), a 100× 1.4 NA Plan-Apo objective lens, and a superresolution module (Live-SR; Gataca systems) based on structured illumination with optical reassignment technique and online processing leading to a two-time resolution improvement (Roth and Heintzmann, 2016). Nine Z-stacks (0.2µm each) were acquired and max-projected prior to model training and analysis.*

**FIGURES**

**I would suggest combining Figures 1 and 2 into a single figure. I'm not sure Figure 3 adds much and could poten2ally be removed. Figures 5 and 6 could probably also be combined and Figure 7 removed. Also, it is not made clear in the figures what sta2s2cal tests were used.**
We agree with the reviewer that figure number should be reduced, in some cases by either removing or combining. Based on these suggestions, the figures were condensed, reordered, or added to the supplement resulting in 5 primary figures. Statistics have been added to figure legends to provide clarity.

**** Figure 4**
***** A: I think enough illustra2ons have been provided already in Figures 1 - 3. I'm not sure this adds a great deal.**
The annotation illustrations were condensed. They now only appear in Figures 2 (filoTips) and 5 (filoSkeleton).

***** B: Error rates should be unambiguously defined in materials and methods. It is not clear to me whether the error rates are being calculated on the basis of the correct number of filopodia being detected in a dataset, or the correct number of filopodia per cell being detected.**
As part of condensing the figures, the confusion matrix in Figure 4 was removed and Figure 4 itself was altered heavily to incorporate correlation plots as recommended by reviewer 1 (see Fig. 5 above). However, the comment was noted and we tried to ensure that unambiguous definitions like this were either removed from the manuscript or elaborated on.

**** Figure 5**
***** A: Again, I'm not sure this adds much, but regardless, isn't the source here phalloidin?**
This was made a supplemental figure instead of primary. It might be useful for some who aren't familiar with what ground truths look like. Phalloidin was made explicit. See Fig. S5 and lines 575-576.

**** Figure 8**
***** B - G: It would appear the authors have pooled together the results of an analysis of WT U2-OS cells and siRNA-treated cells (and HeLa cells?) - the analysis of these two different popula2ons should be displayed separately.**
We thank the reviewer for this comment and see how pooling together different cell types could be problematic under different scenarios. However, the goal is to compare filoSkeleton and FiloQuant filopodia measurements in general (prior version compared filoSkeleton and manual counts in ImageJ), not analyze the different populations for a biological question, thus we decided to prioritize the many other well thought out comments and concerns the reviewer had. Figure 8 was replaced with a correlation plot comparing filoSkeleton and FiloQuant per reviewer recommendations and we agree it is an improvement (see Fig. 5 above).

© 2024. Published by The Company of Biologists under the terms of the Creative Commons Attribution License (https://creativecommons.org/licenses/by/4.0/).

Second decision letter

MS ID#: JOCES/2023/261274

MS TITLE: filoVision: using deep learning and tip markers to automate filopodia analysis

AUTHORS: Casey Eddington, Jessica K Schwartz, and Margaret A. Titus
ARTICLE TYPE: Tools and Resources

We have now reached a decision on the above manuscript.

To see the reviewers' reports and a copy of this decision letter, please go to: https://submit-jcs.biologists.organd click on the 'Manuscripts with Decisions' queue in the Author Area. (Corresponding author only has access to reviews.)

As you will see, two reviewers gave favourable reports but reviewer 3 still has some issues concerning some clarifications and text. He has made a number of suggestions that will require some text amendments to your manuscript. I hope that you will be able to carry these out because I would like to be able to accept your paper. However, if you do not agree with any of these please explain why in your response letter.

Please ensure that you clearly highlight all changes made in the revised manuscript. Please avoid using 'Tracked changes' in Word files as these are lost in PDF conversion.

I should be grateful if you would also provide a point-by-point response detailing how you have dealt with the points raised by the reviewers in the 'Response to Reviewers' box. Please attend to all of the reviewers' comments. If you do not agree with any of their criticisms or suggestions please explain clearly why this is so.

Reviewer 1

*Advance summary and potential significance to field*

*Comments for the author*

The authors have addressed all my comments.
Very nice work.

Reviewer 2

*Advance summary and potential significance to field*

The authors have addressed the concerns. The revised manuscript is much improved, explaining the purpose, use and advantages of filoVision more clearly and this looks a useful tool for filopodia quantifications.

*Comments for the author*

The additional demonstration of transfer learning strongly supports the flexibility and usefulness of the tool for other users where the default model may not be accurate enough.

Some very minor comments:

Line 145 – Ddisc needs definition.

© 2024. Published by The Company of Biologists under the terms of the Creative Commons Attribution License (https://creativecommons.org/licenses/by/4.0/). 

Line 178 – capitalisation of filoTips.

Lines 211-218 - very similar to the methods

Line 670 – missing description of filters?

Reviewer 3

*Advance summary and potential significance to field*

The authors present a revised version of their manuscript describing filoVision, a collection of deep learning-based platform for the automated detection and quantification of filopodia in two-dimensional fluorescence microscopy images. The authors provide an overview of the training and testing of the models underpinning their platform, along with a detailed breakdown of the outputs their platform provides. Results produced using their analysis platform, for live Ddisc cells expressing GFP-DdMyo7 and fixed U2-OS cells labeled with phalloidin and anti-Myo10, are compared to manually-annotated ground truths and other popular software. The implementation of the platform as Google Colab notebooks makes sharing of code trivial. While such a tool would undoubtedly be useful to the broader cell biology community, I still have a number of concerns with the manuscript that need addressing before it is suitable for publication.

*Comments for the author*

I appreciate the authors' efforts to address reviewers' comments and some additions, such as transfer learning, strengthen the authors' arguments.
But I still believe that significant further revision is required before this manuscript is suitable for publication. In particular:

* There are important details surrounding the generation of ground truth data that require clarification

* I'm also still confused by the precise nature of the training data. It seems that for training filoSkeleton, two different cell populations (one stained for actin, one for tip markers) is used? Why? I think a schematic depicting the training process (including ground truth generation) for filoTips, filoSkeleton and the transfer learning would greatly aid the interpretation of these details.
I have also found several examples of language that seems very out of place in a scientific manuscript:

* "We recommend users first try this method, or use Ilastik to generate ground truths as described in filoTips default model training and filopodia detection section"

* "For this reason, we recommend that users tune the existing filoVision models to their datasets via transfer learning, accessible for all with the 2D U-net multilabel ZeroCostDL4Mic notebook."

* "They should feel free to test a small representative dataset and use the default model if they are happy with the results."

* "We encourage future filoVision users to upload their source data and training annotations as well to eventually achieve the goal of having a more generalized model for future users."

Language of this form would be all very well in a tutorial explaining how the software should be used (might I suggest the authors consider writing a post for FocalPlane? - https://focalplane.biologists.com/), but it has no place here.

But overall, I'm afraid I am still finding the manuscript rather difficult to read and comprehend - it is in need of further significant restructuring. The results section is particularly difficult to follow - the section headings as they are don't really aid the reader in interpreting the results. I would suggest an outline as follows - this is obviously based on my own personal opinion and not intended

© 2024. Published by The Company of Biologists under the terms of the Creative Commons Attribution License (https://creativecommons.org/licenses/by/4.0/). 

to be prescriptive, but is mainly intended to illustrate how more liberal use of meaningful section and subsection headings can aid the flow of the manuscript:

* Results
    1. Development of filoVision Platform
        - Describe the development process of filoVision.
    - Explain the components of the platform (filoTips and filoSkeleton).
        - Present the results of model training
  2. Testing filoVision's Performance
    - Present results from tests conducted using filoVision, comparing them with traditional methods.
    - Discuss the accuracy and efficiency of the platform.
  3. Flexibility and Adaptability of filoVision
    - Elaborate on how filoVision adapts to different cell types and datasets.
    - Explain the impact of transfer learning on the platform's performance.
  4. Exploring the Roles of Myo10 and DdMyo7 in Filopodia Formation
    - Present findings on the roles of these proteins based on filoVision analysis.
* Materials and Methods
  1. Image Data Acquisition
    - Detail how the image data for testing filoVision was obtained.
  2. Platform Development
    - Describe the technical aspects of developing filoVision, including the deep learning models used
        - Explain clearly how ground truths were generated
        - Described the process of training filoVision
        - Describe how transfer learning was performed
  3. Data Analysis
    - Explain the methods used to analyze data obtained from filoVision.

This would be my principal recommendation on my attempt to review this version of the manuscript. I address some specific points in the authors' response to my initial review below, but given my difficulty in interpreting the manuscript due to the current layout, it is possible that I have misinterpreted some things.

First paragraph is largely introductory and could be moved to end of introduction.
We agree with the reviewer that the first results paragraph needed improvement. We have edited this paragraph to provide more of an overview of filoVision while still being able to introduce the pla4orm briefly. We chose to end the introduction by discussing why filoVision was initially developed - to address the lack of an automated workflow for measuring tip-marked filopodia in diverse cell types like amoeba (see lines starting at Line 123).
* I'm afraid this still reads like an introductory paragraph to me - see above for a suggested restructuring of the results and methods sections of the manuscript

**Training a filoTips model:
It is stated that "Wild type and filopodia mutant amoeba (myo7 or vasp null) expressing wild type or non-functional mutant DdMyo7 tagged with a variety of fluorophores, including GFP, mCherry, and mNeon were used" - how many of each were used? A much more detailed breakdown of what data was used for training, validating and testing the model should be provided Thank you for pointing this out. First, we mistakenly stated that some cells expressed a nonfunctional DdMyo7, this phrase has been removed (line 459). Information about the cell lines (geneticbackground and DdMyo7-fluorescent protein fusion) is now provided in a new supplementary table, Table S2 where a detailed listing of the training/validation data and test data for each model is provided and separated by sheets. (Lines 459-463)
* While more information about the cell lines used is welcome, what is still lacking is a clear breakdown of exactly what images were manually annotated in ilastik, what images were used for training filoVision, what images were used for validation and what images were used for testing. See Table 1 in this paper for an example: https://dx.doi.org/10.26508/lsa.202302351

It is mentioned that ilastik was used to generate ground truth data, but only very vague details are provided (along with some concerning statements about manual correction being required - what

© 2024. Published by The Company of Biologists under the terms of the Creative Commons Attribution License (https://creativecommons.org/licenses/by/4.0/).

does this mean exactly?) and there is no mention of ilastik in the materials and methods section. The generation of ground truth segmentations should be detailed in a dedicated section under materials and methods.
We have moved this information to the materials and methods section and agree it's more appropriate. We have briefly provided more details regarding Ilastik. (Lines 465-477)
* I think far more detail needs to be provided on the use of ilastik to generate ground truths, as this is absolutely critical to the performance of the trained model. At the moment, this information is buried in a long paragraph under the general heading of "default model training and filopodia detection" when it should be described clearly and unambiguously in a dedicated (sub)section entitled "Generation of ground truths" or something similar. I think it would also be helpful to produce some sort of schematic (it doesn't necessarily have to be terribly complicated) to summarise the training process. I also notice that the ilastik project files are not included in the Github repository - they really should be.

Default data augmentations and default training parameters… what does this mean?
We acknowledge this isn't very informative. These statements were referring to default parameters described in the ZeroCostDL4Mic 2D U-net notebook. We have now added these details to the methods section of the manuscript instead of relying completely on ZeroCostDL4Mic to describe them. (Lines 478-483)
* Again, this is buried in a long paragraph describing a range of different things, when it should be in a dedicated section describing how the model was trained. Also, no information is provided on the training and validation loss after training?

"Intersection over Union" explanation in the results, it should be moved to methods We again thank the reviewer for feedback regarding the structure of the manuscript. The IoU explanation has been reduced and moved to methods. (Lines 483-493)
* At the risk of repeating myself, this is buried in the same paragraph referred to above - there should be a separate (sub)section describing metrics used to evaluate the outputs from the model

Later in the same paragraph, it is stated that "The average IoU for the default filoTips model is 0.76 ± 0.10" - when tested on what? Training data? Unseen test data?
Unseen test data or out-of-sample data – this was clarified further in the revised manuscript. For any evaluation, the test data is of model unseen data and we have now tried to state that explicitly in the manuscript. (Lines 483-485)
* But it is still not clear what "out of sample" means exactly. A table listing precisely what data was used for training and what distinct data was used for testing would be helpful.

The following statement on spatial separation of cells should come later in a general discussion of the limitations of the platform, although I note that this criteria is not tested at any point - how separated do cells have to be in order for filoTips to work?
We now have included a new section at the end of the Discussion "Limitations and other considerations" section in the discussion (lines 380-427) that includes a discussion of this issue. When imaging filopodia, sparse cell density is required, regardless of the analysis method, it is necessary to avoid densely plated samples to prevent neighbor body/filopodia overlaps, which will result in unseen filopodia and an underrepresentation of filopodia number. It is possible for filoTips to assign a filopodium to the wrong cell at normal cell densities appropriate for typical filopodia analysis, but this is rare, again because of the inherent need for spatial separation when imaging filopodia. For example, we commonly observe an average filopodia length of ~2.8 μm for Ddisc cells, therefore we try to plate cells at a density that would provide a separation > 10 μm between cells to avoid overlap regardless of analysis method. The exact density will, of course, depend on the cell type. We are happy to quantify this if necessary, but we decided to prioritize the many other though4ul comments provided by the reviewers.
* I'm not sure it's strictly necessary to quantify this, but a statement similar to the above should certainly be in the manuscript.

How are parameters like area, aspect ratio, pixel intensity extracted? How are filopodia tips assigned to the nearest cell body? How are metrics like filopodia per cell, length, and tip intensity extracted?
These details have been added to the methods section.

© 2024. Published by The Company of Biologists under the terms of the Creative Commons Attribution License (https://creativecommons.org/licenses/by/4.0/).

* Yes, but again, it's a big long paragraph that (a) should be in a distinct subsection and (b) could do with being broken up into smaller paragraphs. A simple schematic to illustrate some of the key concepts would also not be a bad idea.

This section begins with a reference to manual measurements made in ImageJ - it's difficult to follow exactly how manual segmentations and manual measurements were made for the purposes of (a) training the model and (b) evaluating the model. This should all be detailed in a dedicated section under materials and methods.
Thank you for pointing this out. Ground truth generation is discussed above. Manual ImageJ measurements for comparing to filoTips measurements is described in materials and methods. (Lines 621-628)
* Ok, this has its own section, but the section heading could be more informative, like "Manual quantification of filopodia"

"It shows that when trained on the user's data, filoTips is a reliable tool..." - what if it is not trained on the users data? How reliable is it then? Does the model need to be completely retrained in order for it to be in any way useful?
Thanks for pointing out the confusing statement here. The original manuscript didn't include transfer learning but encouraged by your comments and those of the other reviewers, we decided to demonstrate how one could use transfer learning to tune our models to their data. We took steps to see how effective our default filoTips model, trained on amoeboid cells, was at predicting filopodia made by U2-OS and COS-7 cells which are quite different from amoeboid cells. Much to our surprise, the amoeboid model was found to do an excellent job predicting U2-OS and COS-7 filopodia tips, needing minor finetuning to better detect the relatively dim U2-OS and COS-7 cell edge (Fig. 3A below is an example). Model tuning (including ground truth generation and training via ZeroCostDL4Mic) was accomplished by a lab member with no prior deep learning knowledge and minimal guidance in less than 48 hours, demonstrating that the transfer learning barrier is minimal (Fig. 3, below). We believe this initial time investment is a good tradeoff for future filopodia analysis tuned specifically for the user's data, however our models are publicly available, and users are free to use them on their data as well. The transfer learning process is now described in the revised manuscript.
* Indeed, but the procedure used to generate ground truths for transfer learning is unclear. It appears from lines 543 - 554 that some combination of an ImageJ macro (is this one of the macros in the GitHub repo - if so, which one?) and the "default filoTips model" is used, but then ilastik is also mentioned? This is supposed to be the materials and methods section of the manuscript - it should describe accurately and unambiguously exactly what the authors did to generate their data. Recommendations of what "users" should "try" have no place here. Also, I find Figure 3 to be not terribly informative - it doesn't really convey why transfer learning was necessary. For example, what would Figure 3B look like in the absence of transfer learning?

Other points:
- An awful lot of the text in the section "Relationship between Myo10 and DdMyo7 expression and filopodia formation" should probably be moved to the discussion section.
- Why was transfer learning only performed on filoTips, but not filoSkeleton? This seems odd given that only U2-OS cells were used to train filoSkeletion, but it was tested on HeLa cells. It's also difficult to interpret the results in Figure 5 given that that a mixture of two different cell types was used to test. How does filoSkeleton perform when tested on U2-OS cells alone? Or on HeLa cells alone?
- "Additional libraries include: pandas, numpy, glob, shutil, OpenCV, math, scipy, researchpy, matplotlib and seaborn.": Please cite the appropriate literature for these libraries
- There are a number of points in the manuscript where Pearson's correlation coefficient (which is on more than one occasion misspelled as "Person's") seems to be conflated with the Coefficient of Determination. For example, lines 155-157 refer to Pearson's in referencing Figure 2B, but Figure 2B appears to be showing a coefficient of determination (R^2) of 0.99?
- I think more information is also required on the limitations of the author's approach. For example, filoTips relies on the presence of a strong filopodial tip marker to detect both cells and filopodia and uses euclidean distance to assign tips to cells - doesn't this suggest that if distances between cells is approximately equal to or less than twice the average length of a filopodium, then filopodial tips will frequently be assigned to the wrong cell? Given that cells such as HeLa, for

© 2024. Published by The Company of Biologists under the terms of the Creative Commons Attribution License (https://creativecommons.org/licenses/by/4.0/).

example, often grow in "clumps" in close proximity to one another, this relying on distance to assign tips to cells will not be reliable.

Dave Barry,
Francis Crick Institute

---

**Second revision**

Author response to reviewers' comments

We thank the reviewers for their overall positive evaluation of the revised manuscript. While Reviewers #1 and #2 are satisfied with the current manuscript, Reviewer #3 (Dr. Barry) had a number of additional comments. His attention to detail and comments intended to further improve the overall readability and usefulness of the paper are very much appreciated. We have worked to address the comments, however, it must be said that some fall into the category of personal preference for the way one would put together a manuscript. Thus, after carefully consideration, we have chosen not to implement some of this type of suggestion.

**Reviewer 1 Comments for the Author:**
The authors have addressed all my comments.

Very nice work.

*****
**Reviewer 2 Advance Summary and Potential Significance to Field:**
The authors have addressed the concerns. The revised manuscript is much improved, explaining the purpose, use and advantages of filoVision more clearly and this looks a useful tool for filopodia quantifications.

**Reviewer 2 Comments for the Author:**
The additional demonstration of transfer learning strongly supports the flexibility and usefulness of the tool for other users where the default model may not be accurate enough.

Some very minor comments:

Line 145 – Ddisc needs definition.
Added (*Dictyostelium*) after Ddisc (now line 140)

Line 178 – capitalisation of filoTips.
This typo has been fixed (now line 173).

Lines 211-218 - very similar to the methods
The reviewer is correct here - it is nearly identical to text in the Methods. This likely happened during reorganization of the manuscript where we missed the duplication. The text is now deleted.

Line 670 – missing description of filters?
The information is now provided in the Methods (lines 695 - 697).

**Reviewer 3 Advance Summary and Potential Significance to Field:**
The authors present a revised version of their manuscript describing filoVision, a collection of deep learning-based platform for the automated detection and quantification of filopodia in two-dimensional fluorescence microscopy images. The authors provide an overview of the training and testing of the models underpinning their platform, along with a detailed breakdown of the outputs their platform provides. Results produced using their analysis platform, for live Ddisc cells expressing GFP-DdMyo7 and fixed U2-OS cells labeled with phalloidin and anti-Myo10, are compared

© 2024. Published by The Company of Biologists under the terms of the Creative Commons Attribution License (https://creativecommons.org/licenses/by/4.0/).

to manually-annotated ground truths and other popular software. The implementation of the platform as Google Colab notebooks makes sharing of code trivial. While such a tool would undoubtedly be useful to the broader cell biology community, I still have a number of concerns with the manuscript that need addressing before it is suitable for publication.

**Reviewer 3 Comments for the Author:**

I appreciate the authors' efforts to address reviewers' comments and some additions, such as transfer learning, strengthen the authors' arguments.

But I still believe that significant further revision is required before this manuscript is suitable for publication. In particular:

* There are important details surrounding the generation of ground truth data that require clarification
* I'm also still confused by the precise nature of the training data. It seems that for training filoSkeleton, two different cell populations (one stained for actin, one for tip markers) is used? Why? I think a schematic depicting the training process (including ground truth generation) for filoTips, filoSkeleton and the transfer learning would greatly aid the interpretation of these details.

The reviewer raises a critically important lingering issue and we have worked to clarify how ground truths were generated. A schematic for generating ground truths is a great idea and new supplemental figure 5 is now provided that illustrates these steps.

Detailed information about the training data itself was added to Table 2 in the previous revision cycle. We believe this to be a sufficient description of the data used for training.

As explained previously and in the text, filoSkeleton uses two models - one to predict cell bodies and filopodia stalks from an actin-labeled image - one to predict filopodia tips from a tip-labeled image. We had access to a combination of images where cells were labeled for 1) both actin and filopodia tips, 2) only actin was labeled, and 3) only tips were labeled. Therefore, the actin channels from all images were used for training the model predicting bodies and stalks, while the tip channels from all images were used for training the model predicting tips. We could have taken a different approach and used one model, but initial trials suggested the two-model approach performed better, and we fail to see why using two models in this manner could be problematic.

© 2024. Published by The Company of Biologists under the terms of the Creative Commons Attribution License (https://creativecommons.org/licenses/by/4.0/).

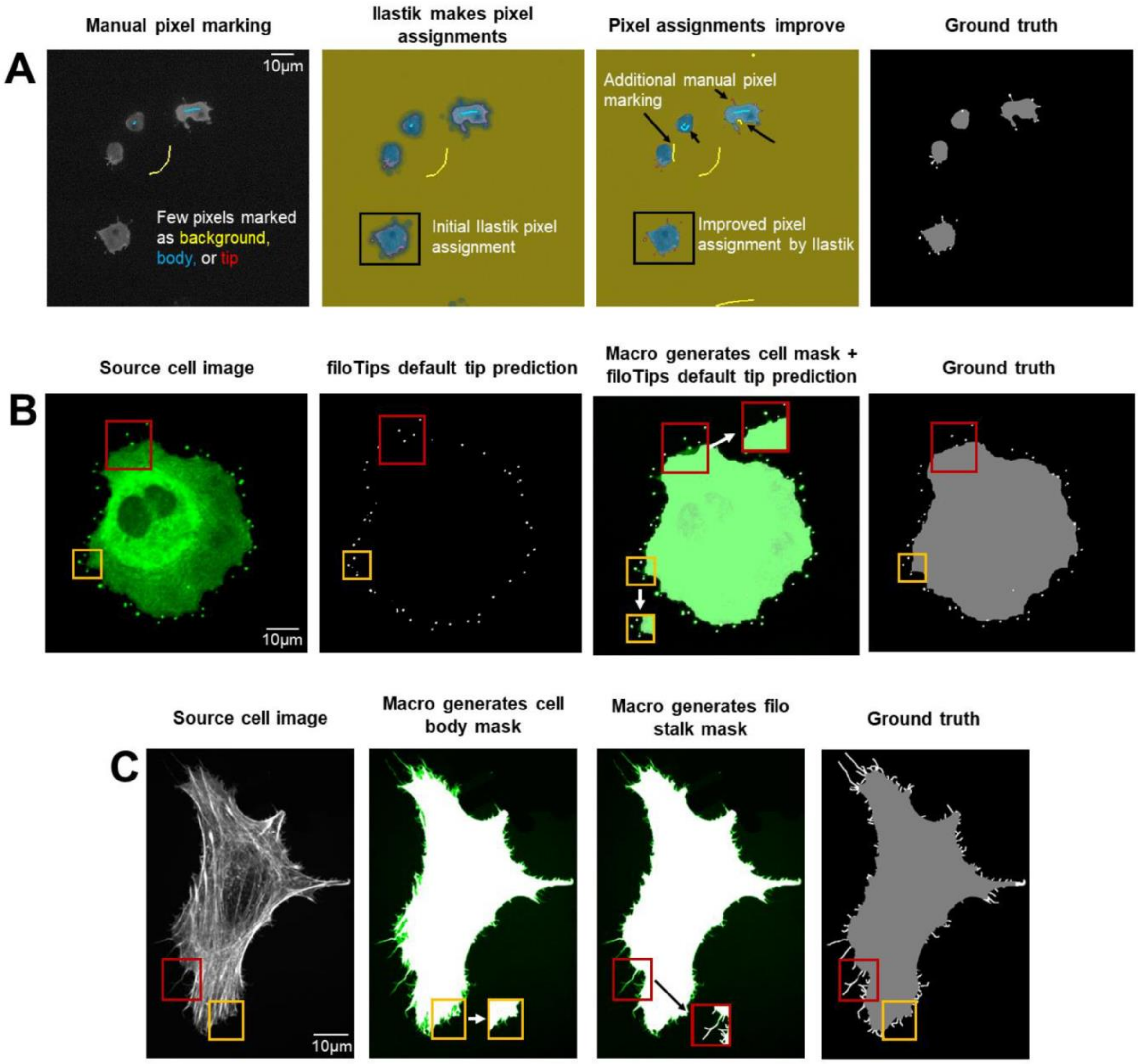


**Figure S5. Ground truth generation methods for filoVision models. (A)** Workflow for generation of ground truths using Ilastik. Representative source cells (Ddisc cells expressing DdMyo7) are annotated in Ilastik until pixels are correctly assigned by Ilastik. **(B)** The ImageJ macro "filoTips Ground Truth Generator" generates a mask for the cell body of a representative COS-7 cell expressing Myo10, which is combined with the filoTips default model prediction for filopodia tips to generate ground truths. **(C)** The ImageJ macro "filoSkeleton Body_Stalk Ground Truth generator" generates a mask for the cell body of a representative HeLa cell stained with phalloidin, first for the cell body, then for filopodia stalks. All ground truths generated by these methods were used to train filoVision models. The boxed regions in B and C illustrate examples of regions found to be incompletely assigned following manually inspected and then improved after further assignment by Ilastik.

I have also found several examples of language that seems very out of place in a scientific manuscript:

* "We recommend users first try this method, or use Ilastik to generate ground truths as described in filoTips default model training and filopodia detection section"

* "For this reason, we recommend that users tune the existing filoVision models to their datasets via transfer learning, accessible for all with the 2D U-net multilabel ZeroCostDL4Mic notebook."

* "They should feel free to test a small representative dataset and use the default model if they are happy with the results."

© 2024. Published by The Company of Biologists under the terms of the Creative Commons Attribution License (https://creativecommons.org/licenses/by/4.0/). 

* “We encourage future filoVision users to upload their source data and training annotations as well to eventually achieve the goal of having a more generalized model for future users.”

Language of this form would be all very well in a tutorial explaining how the software should be used (might I suggest the authors consider writing a post for FocalPlane? – https://focalplane.biologists.com/), but it has no place here.

Thank you for pointing these sentences out. They were included originally because we expect the primary readers to be cell biologists who might not have deep learning knowledge and we wanted to emphasize that our current models should not be considered universal models. However, as we have had a lot of success in using our default models on different cell types we do wish to convey the fact that the model can be easily fine-tuned if needed. All of the sentences listed by the reviewer have been removed and a simple sentence that makes our point has been added to the “Limitations” section on lines 396 - 400:

“…..model performance would be at its highest by tuning the existing filoVision models by performing transfer learning with a user’s images of the cell type and typical imaging conditions. This can easily be accomplished with the 2D U-net ZeroCostDL4Mic notebooks..”

We appreciate the fantastic suggestion to post a tutorial for FocalPlane! This would allow us to convey some of the considerations of how to train one’s own data that do not really belong in the manuscript. We are working on the post now and will submit it once the manuscript is (hopefully) online in the coming weeks.

But overall, I'm afraid I am still finding the manuscript rather difficult to read and comprehend - it is in need of further significant restructuring. The results section is particularly difficult to follow - the section headings as they are don't really aid the reader in interpreting the results. I would suggest an outline as follows - this is obviously based on my own personal opinion and not intended to be prescriptive, but is mainly intended to illustrate how more liberal use of meaningful section and subsection headings can aid the flow of the manuscript:

* Results
1. Development of filoVision Platform
- Describe the development process of filoVision.
- Explain the components of the platform (filoTips and filoSkeleton).
- Present the results of model training
2. Testing filoVision's Performance
- Present results from tests conducted using filoVision, comparing them with traditional methods.
- Discuss the accuracy and efficiency of the platform.
3. Flexibility and Adaptability of filoVision
- Elaborate on how filoVision adapts to different cell types and datasets.
- Explain the impact of transfer learning on the platform's performance.
4. Exploring the Roles of Myo10 and DdMyo7 in Filopodia Formation
- Present findings on the roles of these proteins based on filoVision analysis.
* Materials and Methods
1. Image Data Acquisition
- Detail how the image data for testing filoVision was obtained.
2. Platform Development
- Describe the technical aspects of developing filoVision, including the deep learning models used
- Explain clearly how ground truths were generated
- Described the process of training filoVision
- Describe how transfer learning was performed
3. Data Analysis
- Explain the methods used to analyze data obtained from filoVision.

This would be my principal recommendation on my attempt to review this version of the manuscript. I address some specific points in the authors' response to my initial review below, but given my difficulty in interpreting the manuscript due to the current layout, it is possible that I have misinterpreted some things.

© 2024. Published by The Company of Biologists under the terms of the Creative Commons Attribution License (https://creativecommons.org/licenses/by/4.0/). 

It is dismaying to hear that the reviewer is still finding the manuscript difficult to follow/comprehend. It has been our goal all along to have the work be as accessible as possible to all readers and understand that it our responsibility to present the work in this way.

We have carefully considered each suggestion here and tried make adjustments where we agreed and/or could but, as noted above and acknowledged by the reviewer, some suggestions could be seen as a matter of personal preference. Also, some of the proposed reorganization would require a fair bit of reworking of the manuscript in some spots so we did not always make the suggested changes. The approach we chose was to add subtitles in sections that the reviewer found difficult to get through in order to break up long stretches of text. These also serve as guideposts for the information being presented in a particular section.

First paragraph is largely introductory and could be moved to end of introduction.
We agree with the reviewer that the first results paragraph needed improvement. We have edited this paragraph to provide more of an overview of filoVision while still being able to introduce the pla4orm briefly. We chose to end the introduction by discussing why filoVision was initially developed - to address the lack of an automated workflow for measuring tip-marked filopodia in diverse cell types like amoeba (see lines starting at Line 123).
* I'm afraid this still reads like an introductory paragraph to me - see above for a suggested restructuring of the results and methods sections of the manuscript

The goal of this first paragraph is to give the reader a general overview of filovision and what they would be reading about in the Results. The title for the subsection (line 118) has been slightly modified to convey its purpose"

Overview of filoVision: a flexible automated filopodia analysis platform

**Training a filoTips model:
It is stated that "Wild type and filopodia mutant amoeba (myo7 or vasp null) expressing wild type or non-functional mutant DdMyo7 tagged with a variety of fluorophores, including GFP, mCherry, and mNeon were used" - how many of each were used? A much more detailed breakdown of what data was used for training, validating and testing the model should be provided
Thank you for pointing this out. First, we mistakenly stated that some cells expressed a nonfunctional DdMyo7, this phrase has been removed (line 459). Information about the cell lines (geneticbackground and DdMyo7-fluorescent protein fusion) is now provided in a new supplementary table, Table S2 where a detailed listing of the training/validation data and test data for each model is provided and separated by sheets. (Lines 459-463)
* While more information about the cell lines used is welcome, what is still lacking is a clear breakdown of exactly what images were manually annotated in ilastik, what images were used for training filoVision, what images were used for validation and what images were used for testing. See Table 1 in this paper for an example: https://dx.doi.org/10.26508/lsa.202302351

The methods used to generate ground truths for each dataset was included in the Methods (and now we have added a supp figure for further clarification and added subheadings as recommended). Information on training and validation data (training data described in Table 2 and train/validation split is described in the text) and test data has already been provided in Table 2. We have also added a column to Table 2 which further clarifies the method used to generate ground truths. The title of each sheet makes it clear which batch of images were used for testing or training, and which model they are associated with. Readers can then refer to the new supp. figure 5 also if desired.

It is mentioned that ilastik was used to generate ground truth data, but only very vague details are provided (along with some concerning statements about manual correction being required – what does this mean exactly?) and there is no mention of ilastik in the materials and methods section. The generation of ground truth segmentations should be detailed in a dedicated section under materials and methods.
We have moved this information to the materials and methods section and agree it's more appropriate. We have briefly provided more details regarding Ilastik. (Lines 465-477)

© 2024. Published by The Company of Biologists under the terms of the Creative Commons Attribution License (https://creativecommons.org/licenses/by/4.0/).

* I think far more detail needs to be provided on the use of ilastik to generate ground truths, as this is absolutely critical to the performance of the trained model. At the moment, this information is buried in a long paragraph under the general heading of “default model training and filopodia detection” when it should be described clearly and unambiguously in a dedicated (sub)section entitled “Generation of ground truths” or something similar. I think it would also be helpful to produce some sort of schematic (it doesn’t necessarily have to be terribly complicated) to summarise the training process. I also notice that the ilastik project files are not included in the Github repository – they really should be.

Thank you for the schematic recommendation. A new supplemental figure that illustrates how Ilastik is used to generate ground truths is now provided (Supp Fig 5). We have uploaded the ground truths obtained from the Ilastik files. We did not upload the project files because we fail to see how they could be broadly useful as they are quite specific to the small image batches used for each Ilastik project file. Instead, we would encourage users to start their own project files, this point will be made in our tutorial.

We have broken things up and rearranged them further in efforts to find some middle ground with the reviewer’s suggested organization and have included subtitles so the reader can quickly see which details are presented in the Methods (starting on line 438).

Default data augmentations and default training parameters… what does this mean?
We acknowledge this isn’t very informative. These statements were referring to default parameters described in the ZeroCostDL4Mic 2D U-net notebook. We have now added these details to the methods section of the manuscript instead of relying completely on ZeroCostDL4Mic to describe them. (Lines 478-483)
* Again, this is buried in a long paragraph describing a range of different things, when it should be in a dedicated section describing how the model was trained. Also, no information is provided on the training and validation loss after training?

We would also like to thank the reviewer for the recommending putting this information in a dedicated section. Thus, we now include subtitles in the methods section, as mentioned above.
Training and validation loss plots were included in the original manuscript, but were removed from the manuscript itself due to comments about the excessive amounts of figures. To avoid excessive supplemental figures, some existing supp. figures and the added training and validation loss plots have been condensed into new Figure S4. Please see below.

© 2024. Published by The Company of Biologists under the terms of the Creative Commons Attribution License (https://creativecommons.org/licenses/by/4.0/).

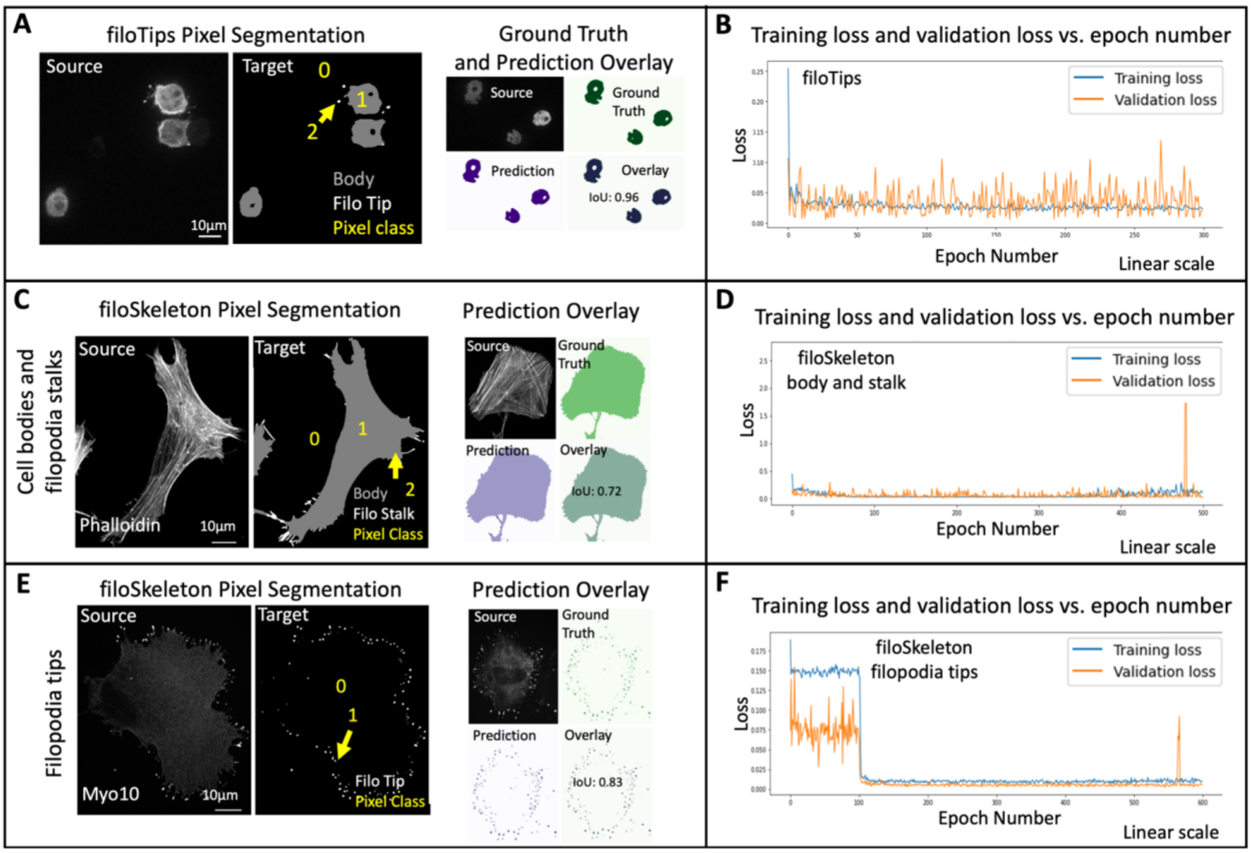


**Figure S4. filoVision model training overview. (A)** Representative images of a source and target pair. Source: Live cell images of Ddisc cells expressing GFP-DdMyo7. Target: Segmentation mask of the source image where all pixels have been classified into 3 groups: background-0 (black), body-1 (gray), and filopodia tips-2 (white) assigned pixel class (yellow numbers). The arrow indicates a representative filopodia tip. Representative ground truth and default filoTips model prediction overlay representing a source (white) and ground truth (green) pair along with the trained model prediction (light purple) and an overlay of the model prediction and ground truth (dark blue). In the representative example, the overlay Intersection-over-union (IoU) score was 0.96. **(B)** Plot of training and validation loss by epoch number during training of the filoTips default model. **(C)** Representative images of a labeled cytoskeleton source and target pair. Source: Fixed imaging of a U2-OS cell ectopically expressing eGFP-Myo10 stained with anti-Myo10 antibodies. Target: Segmentation mask of the source image where all pixels have been classified into 3 groups: background-0 (black), body-1 (gray), and filopodia stalks-2 (white) assigned pixel class (yellow numbers). The arrow indicates a representative filopodia stalk. Representative ground truth and filoSkeleton body and stalk model prediction overlays. **(D)** Plot of training and validation loss by epoch number during training of the filoSkeleton body and stalk model. **(E)** Representative images of a labeled filopodia tips source and target pair. Source: Image of a U2-OS cell ectopically expressing eGFP-Myo10 fixed and stained with anti-Myo10 showing labeled filopodia tips. Target: Binary segmentation of the source image where all pixels have been classified into 2 groups: background-0 (black) and filopodia tips-1 (white) and pixel class (yellow numbers). The arrow indicates a representative filopodia tip. Representative ground truth and filoSkeleton filopodia tips model prediction overlays. **(F)** Plot of training and validation loss by epoch number during training of the filoSkeleton filopodia tips model.

“Intersection over Union” explanation in the results, it should be moved to methods
We again thank the reviewer for feedback regarding the structure of the manuscript. The IoU explanation has been reduced and moved to methods. (Lines 483-493)
* At the risk of repeating myself, this is buried in the same paragraph referred to above - there should be a separate (sub)section describing metrics used to evaluate the outputs from the model

© 2024. Published by The Company of Biologists under the terms of the Creative Commons Attribution License (https://creativecommons.org/licenses/by/4.0/).

This is now included in a separate subsection in the Methods - *Model Evaluation* (starting on line 483).

Later in the same paragraph, it is stated that “The average IoU for the default filoTips model is 0.76 ± 0.10” – when tested on what? Training data? Unseen test data?
Unseen test data or out-of-sample data – this was clarified further in the revised manuscript. For any evaluation, the test data is of model unseen data and we have now tried to state that explicitly in the manuscript. (Lines 483-485)
* But it is still not clear what “out of sample” means exactly. A table listing precisely what data was used for training and what distinct data was used for testing would be helpful.

This information was included in the first revision in the form of Table 2. One can find the training and test data here in separate sheets. We have now further defined “out-of-sample” on line 148: “out-of-sample (independent test dataset previously unseen by model).”

The following statement on spatial separation of cells should come later in a general discussion of the limitations of the platform, although I note that this criteria is not tested at any point – how separated do cells have to be in order for filoTips to work?
We have included a section at the end of the Discussion “Limitations and other considerations” section in the discussion (lines 380-427) that includes a discussion of this issue. When imaging filopodia, sparse cell density is required, regardless of the analysis method, it is necessary to avoid densely plated samples to prevent neighbor body/filopodia overlaps, which will result in unseen filopodia and an underrepresentation of filopodia number. It is possible for filoTips to assign a filopodium to the wrong cell at normal cell densities appropriate for typical filopodia analysis, but this is rare, again because of the inherent need for spatial separation when imaging filopodia. For example, we commonly observe an average filopodia length of ~2.8 µm for Ddisc cells, therefore we try to plate cells at a density that would provide a separation > 10 µm between cells to avoid overlap regardless of analysis method. The exact density will, of course, depend on the cell type. We are happy to quantify this if necessary, but we decided to prioritize the many other though4ul comments provided by the reviewers.
* I’m not sure it’s strictly necessary to quantify this, but a statement similar to the above should certainly be in the manuscript.

We have now expanded on existing text in the manuscript. It fits in well with the section on how poor image quality may lead to poor analysis, in the limitations section of the conclusion.
The following has been added (lines 369 - 378):

“As with other filopodia analysis workflows, it is necessary to avoid densely plated samples to prevent overlap between filopodia and neighboring cells. This can result in obscured filopodia and an underrepresentation of filopodia number, or assignment of filopodia to the incorrect cell. filoTips uses Euclidean distance to assign filopodia tips to cell bodies. Therefore, cells should be plated at a density where filopodia tips are closer to the bodies they belong to as opposed to neighboring cell bodies. The exact density will depend on the cell type and their average filopodia length. This limitation is minor considering most will plate cells at a low density regardless of analysis method to prevent occlusion of filopodia by neighboring cells and their filopodia.”

How are parameters like area, aspect ratio, pixel intensity extracted? How are filopodia tips assigned to the nearest cell body? How are metrics like filopodia per cell, length, and tip intensity extracted?
These details have been added to the methods section.
* Yes, but again, it’s a big long paragraph that (a) should be in a distinct subsection and (b) could do with being broken up into smaller paragraphs. A simple schematic to illustrate some of the key concepts would also not be a bad idea.

The suggestion to put this information in a distinct subsection and break it up into smaller paragraphs are good ones and we have done so (starting with line 497 - *Detection and measurement of cell bodies*).

This section begins with a reference to manual measurements made in ImageJ – it’s difficult to follow exactly how manual segmentations and manual measurements were made for the purposes

© 2024. Published by The Company of Biologists under the terms of the Creative Commons Attribution License (https://creativecommons.org/licenses/by/4.0/).

of (a) training the model and (b) evaluating the model. This should all be detailed in a dedicated section under materials and methods.
Thank you for pointing this out. Ground truth generation is discussed above. Manual ImageJ measurements for comparing to filoTips measurements is described in materials and methods. (Lines 621-628)
* Ok, this has its own section, but the section heading could be more informative, like “Manual quantification of filopodia”

Agreed – the section heading (line 645) has been changed to:

Manual quantification of filopodia – analysis comparison and statistics

“It shows that when trained on the user’s data, filoTips is a reliable tool...” – what if it is not trained on the users data? How reliable is it then? Does the model need to be completely retrained in order for it to be in any way useful?
Thanks for pointing out the confusing statement here. The original manuscript didn’t include transfer learning but encouraged by your comments and those of the other reviewers, we decided to demonstrate how one could use transfer learning to tune our models to their data. We took steps to see how effective our default filoTips model, trained on amoeboid cells, was at predicting filopodia made by U2-OS and COS-7 cells which are quite different from amoeboid cells. Much to our surprise, the amoeboid model was found to do an excellent job predicting U2-OS and COS-7 filopodia tips, needing minor finetuning to better detect the relatively dim U2-OS and COS-7 cell edge (Fig. 3A below is an example). Model tuning (including ground truth generation and training via ZeroCostDL4Mic) was accomplished by a lab member with no prior deep learning knowledge and minimal guidance in less than 48 hours, demonstrating that the transfer learning barrier is minimal (Fig. 3, below). We believe this initial time investment is a good tradeoff for future filopodia analysis tuned specifically for the user’s data, however our models are publicly available, and users are free to use them on their data as well. The transfer learning process is now described in the revised manuscript.
* Indeed, but the procedure used to generate ground truths for transfer learning is unclear. It appears from lines 543 – 554 that some combination of an ImageJ macro (is this one of the macros in the GitHub repo – if so, which one?) and the “default filoTips model” is used, but then ilastik is also mentioned? This is supposed to be the materials and methods section of the manuscript – it should describe accurately and unambiguously exactly what the authors did to generate their data. Recommendations of what “users” should “try” have no place here.

Clarification on how ground truths were generated has now been added as a supplemental figure (Sup Fig 5). The language has also been changed to more accurately reflect what the authors did, rather than suggestions for potential filoTips users (see above).

Also, I find Figure 3 to be not terribly informative – it doesn’t really convey why transfer learning was necessary. For example, what would Figure 3B look like in the absence of transfer learning?

The purpose of the transfer learning section and of Figure 3 is to convey that model finetuning can easily be done if needed. We think this is fairly clear in the text of the manuscript (section starting on line176 in the Results). To better illustrate the benefit of transfer learning in this figure the correlation between manual counts and pre-transfer learning as well as the correlation between manual counts and post-transfer learning has now been added (see plot for results of the default model versus a fine-tuned model from transfer learning. As one can see there is an increase in performance, but it is notable that the model performed fairly well prior to transfer learning. An argument could be made that it wasn’t necessary, but again our purpose was to convey that it could easily be done.

If anything, the result actually highlights the universal capabilities of the default filoTips model, as the mammalian data used in this panel is quite different from the amoeboid data used to train the default model.

© 2024. Published by The Company of Biologists under the terms of the Creative Commons Attribution License (https://creativecommons.org/licenses/by/4.0/).

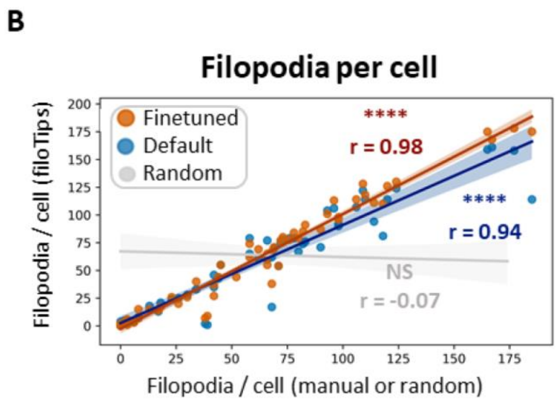


Other points:
- An awful lot of the text in the section "Relationship between Myo10 and DdMyo7 expression and filopodia formation" should probably be moved to the discussion section.

This is a good point. We acknowledge some of the text found here is well suited for a discussion section. After you pointed this out, we thought a lot about it. Our current discussion is really focusing in on the pros and cons of filoVision and looking at the big picture. By adding in this very specific text, we believe it would distract from the main purpose of the discussion. Therefore, we decided to keep this text in the results section, but we do agree it is also suited for a discussion section.

- Why was transfer learning only performed on filoTips, but not filoSkeleton? This seems odd given that only U2-OS cells were used to train filoSkeletion, but it was tested on HeLa cells. It's also difficult to interpret the results in Figure 5 given that that a mixture of two different cell types was used to test. How does filoSkeleton perform when tested on U2-OS cells alone? Or on HeLa cells alone?

Transfer learning was performed on filoTips to provide a 'proof of principle' example of how easily this could be done if needed. Our purpose was to provide an example, not to provide a number of different finetuned models. A mixture of the two cell types (which are very similar in appearance) was used because the focus was on filopodia counting, not a biological question. If anything, this adds a little diversity to the testing which we fail to see as problematic. Although we kept the combined dataset for the figure, we calculated Pearson's correlation coefficients for the individual cell types (U2-OS r = 0.81; HeLa r = 0.83) to demonstrate that the model performed similarly for both cell types.

Lines 333 - 335

"…A strong correlation between filoSkeleton and FiloQuant filopodia number measurements was observed with a correlation coefficient of 0.82 (combined U2-OS and HeLa r = 0.82; U2-OS alone r = 0.81; HeLa alone r = 0.83; Fig. 5B)…"

- "Additional libraries include: pandas, numpy, glob, shutil, OpenCV, math, scipy, researchpy, matplotlib and seaborn.": Please cite the appropriate literature for these libraries

Thank you for pointing out this oversight. These citations have been added (lines 739 - 741).

- There are a number of points in the manuscript where Pearson's correlation coefficient (which is on more than one occasion misspelled as "Person's") seems to be conflated with the Coefficient of Determination. For example, lines 155-157 refer to Pearson's in referencing Figure 2B, but Figure 2B appears to be showing a coefficient of determination (R^2) of 0.99?

Thank you for pointing out the spelling errors (we blame autocorrect!). The spelling for Pearson's has been corrected in every instance. The reviewer is also absolutely right to point out R^2 was the incorrect label and should be "r". We thank the reviewer for pointing this out and it has been edited in the manuscript and figures.

© 2024. Published by The Company of Biologists under the terms of the Creative Commons Attribution License (https://creativecommons.org/licenses/by/4.0/).

- I think more information is also required on the limitations of the author's approach. For example, filoTips relies on the presence of a strong filopodial tip marker to detect both cells and filopodia and uses euclidean distance to assign tips to cells – doesn't this suggest that if distances between cells is approximately equal to or less than twice the average length of a filopodium, then filopodial tips will frequently be assigned to the wrong cell? Given that cells such as HeLa, for example, often grow in "clumps" in close proximity to one another, this relying on distance to assign tips to cells will not be reliable.

A strong, reliable tip marker is indeed needed for filoTips and is discussed in the "limitations" section. We believe a signal strength requirement to be a minor limitation common among many available workflows. As for Euclidean distance, the reviewer is correct that spatial separation of cells is required, is a limitation, and should be discussed. This limitation was originally discussed under the "limitations" section in the discussion, however it has been expanded. Note that we argue it is a very minor limitation. As stated above in another response, filopodia analysis workflows should require spatial separation of cells! If you have cells growing in "clumps" it is very challenging to get an accurate measurement of filopodia regardless of filopodia analysis workflow, as many filopodia will likely be obscured by neighboring cells or overlapping filopodia. Because of this, we argue that cells growing in "clumps" should be ignored regardless of the analysis method. We would also like to note, that it is very easy to plate cells at a low enough density to where spatial separation isn't a problem (spacing far more than the distance of the length of a typical filopodium) and is routinely performed by our lab and others. We have shown this indirectly multiple times by comparing filopodia number counts by filoVision to manual counts – showing they are correctly being assigned. If spatial separation is problematic for a potential user, perhaps they should consider lowering the density at which they are plating the cells (even if not using filoVision). Same for the response above, we have added the following to the manuscript, expanding on what was there:

Lines 369 - 378

"As with other filopodia analysis workflows, it is necessary to avoid densely plated samples to prevent overlap between filopodia and neighboring cells. This can result in obscured filopodia and an underrepresentation of filopodia number, or assignment of filopodia to the incorrect cell. filoTips uses Euclidean distance to assign filopodia tips to cell bodies. Therefore, cells should be plated at a density where filopodia tips are closer to the bodies they belong to as opposed to neighboring cell bodies. The exact density will depend on the cell type and their average filopodia length. This limitation is minor considering most will plate cells at a low density regardless of analysis method to prevent occlusion of filopodia by neighboring cells and their filopodia."

---

Third decision letter

MS ID#: JOCES/2023/261274

MS TITLE: filoVision: using deep learning and tip markers to automate filopodia analysis

AUTHORS: Casey Eddington, Jessica K Schwartz, and Margaret A. Titus
ARTICLE TYPE: Tools and Resources

Happy new Year and I am pleased to tell you that your manuscript has been accepted for publication in Journal of Cell Science, pending standard ethics checks.

© 2024. Published by The Company of Biologists under the terms of the Creative Commons Attribution License (https://creativecommons.org/licenses/by/4.0/).
